# Risk factors for modified vaccine effectiveness of the live attenuated zoster vaccine among the elderly in England^☆^

**DOI:** 10.1016/j.jvacx.2019.100007

**Published:** 2019-01-29

**Authors:** Kaatje Bollaerts, Maria Alexandridou, Thomas Verstraeten

**Affiliations:** P95 Epidemiology and Pharmacovigilance, Koning Leopold III Laan 1, 3001 Leuven, Belgium

**Keywords:** Herpes zoster, Shingles, Vaccine, Vaccine failure, Effectiveness

## Abstract

**Background:**

The United Kingdom introduced routine vaccination with the live-attenuated zoster vaccine for 70 year-olds in 2013, with the vaccine also offered to 79 year-olds as part of a catch-up campaign. In the subsequent years, the catch-up campaign was extended to also include adults aged 78 years. We investigated 14 pre-identified potential risk factors for potential modified vaccine effectiveness.

**Methods:**

This retrospective cohort study in England included subjects born in 1943–1946 (the routine cohort) and in 1934–1937 (the catch-up cohort). We used the Clinical Practice Research Datalink (CPRD) to identify herpes zoster (HZ) cases and the risk factors: age, gender, ethnicity, socio-economic status, asthma, type 2 diabetes, chronic obstructive pulmonary disease, smoking, body mass index, immunosuppression, history of HZ, co-administration with influenza or pneumococcal vaccine. We derived HZ incidence by risk groups, overall vaccine effectiveness (VE) and modified VE expressed as relative differences in VE from Poisson regression models.

**Results:**

Overall VE was 66.8% [95% CI: 62.2; 71.0]. Two out of the 14 investigated risk factors modified the HZ VE. Notably, lower VE was observed in diabetics and in persons with a history of HZ with relative differences in VE of –22·2%, [95% CI: −39·6, −4·5] and –22·5%, [95% CI: −44·9, −0·1].

**Conclusions:**

Live-attenuated zoster vaccine protection against HZ was lower in type 2 diabetics and in subjects with a history of HZ. Contrary to clinical trial results, age did not affect the observed VE. Further study is required to gain insights into why certain risk groups are less protected. Identifying and understanding the effect modifiers of VE is important for future vaccine development as well as vaccine recommendations.

## Highlights

• Diabetes or prior herpes zoster result in 22–23% lower vaccine effectiveness (VE).
• These groups represent 28.5% of the population for whom the vaccine is recommended.
• Key to study VE by risk groups for vaccine recommendations and development.

## 1 Introduction

Herpes zoster (HZ), or shingles, results from reactivation of latent varicella-zoster virus (VZV) that reside in nerve cells following a primary infection manifesting as chickenpox, typically acquired during childhood. HZ is characterized by a unilateral dermatomal rash and pain, which usually lasts between two weeks and one month [1]. The incidence and severity of HZ increases with age, peaking at 75–85 years of age [2]. The most common complication is persistent chronic pain or post-herpetic neuralgia (PHN), lasting months after the rash has healed and significantly impairing quality of life [3]. In the United Kingdom, the estimated incidence among those 50 years and older is 5.23/1000 person-years, with about 20% of patients developing PHN at least one month after HZ diagnosis [2].

Since 2013, the UK has offered the zoster vaccine Zostavax® to adults from 70 years of age. Zostavax®, a single-dose live-attenuated herpes zoster vaccine, was approved by the European Medicines Agency in 2012 for the prevention of HZ and PHN in adults aged 50 years and older [4]. The vaccine is contra-indicated for persons following immunosuppressive therapy or otherwise with a weakened immune system, as well as for pregnant women and those with active tuberculosis [4]. In a clinical trial setting Zostavax® demonstrated a vaccine efficacy against HZ of 51.3% (95% CI: 44.2–57.6%) in adults aged 60–69 years, and 37.6% in those aged 70 years or older [5]. Following its introduction into the UK on 1st September 2013, the vaccine has been routinely offered to adults at 70 years of age, and to adults aged 79 years as part of a catch-up campaign [6]. In the subsequent years, the catch-up campaign was extended to also include adults aged 78 years. Vaccination coverage one year after vaccine introduction was 61.8% for the routine cohort and 59.6% in the catch-up cohort [6]. Several observational studies of the vaccine effectiveness (VE) of Zostavax® have been conducted in the United States, where it has been licensed for adults aged ≥ 60 years since 2006. These studies reported overall VE estimates from 33% to 55% against HZ [7], [8], [9], [10]. In line with their earlier vaccination impact estimations [11], a recent study in the UK with a median follow-up time of 1.42 person-years post-vaccination found a slightly higher VE against HZ (64%; 95% CI: 60, 68%) [12], likely explained by the shorter follow-up period after vaccination in this study compared to the other studies.

As VE might be influenced by many factors, including host factors, logistical issues and epidemiological factors [13], we performed this observational cohort study to add to the existing knowledge by investigating host factors for Zostavax® vaccine failure against HZ in elderly over 70 years of age in England. Based on our review of the literature, we investigated the following potential risk factors: age, gender, ethnicity, socio-economic status, asthma, type 2 diabetes, chronic obstructive pulmonary disease (COPD), smoking, body mass index (BMI), immunocompromised conditions or immunosuppressive or immuno-modulating therapy, a history of HZ prior to vaccination and co-administration with influenza and with pneumococcal vaccine.

## 2 Methods

The study protocol was approved by the Independent Scientific Advisory Committee for MHRA database research [ISAC protocol. 17_081].

### 2.1 Data sources

Data were extracted from the Clinical Practice Research Datalink General Practice Database (CRPD-GOLD) linked with the Hospital Episode Statistics Admitted Patient Care data (HES APC) and the Small area level deprivation data (IMD – 2015). The CPRD-GOLD is a primary care database of anonymized patient electronic medical records (EMRs) and is representative of the UK population with regards to age, sex and ethnicity [14]. The CPRD-GOLD currently covers quality data on 6.9% of the UK population [14] and has been validated for use in pharmacoepidemiologic research [15]. Patient information is mostly entered using Read codes, being standard clinical terminology used in UK general practice. Prescriptions are entered using Gemscript codes based on the NHS dictionary of medicines and devices.

The HES APC includes data on all admissions and visits to National Health Service (NHS) hospitals in England. It is estimated that 98–99% of hospital activity in England is funded by the NHS [16]. The information in HES APC is recorded using the International Classification of Diseases-10 codes (ICD-10). Approximately 75% of the CPRD-GOLD practices in England are linked to the HES APC.

The IMD-2015 database contains a range of socio-economic measures at small area level in England, including the composite measure ‘Index of Multiple Deprivation’ (IMD).

### 2.2 Study cohort and follow-up

We retrospectively analysed data from subjects born in 1943–46 (routine cohorts) and in 1934–37 (catch-up cohorts). Zoster events were identified starting from the 1st January of the year the majority of the birth cohort (i.e. those with their birthday prior to September 1st) became eligible for vaccination. For all routine cohorts, this is the year of the 70th birthday. For the 1934–35 and the 1936–37 catch-up cohorts, this is year of the 79th and the 78th birthdays, respectively. Subjects were included if they had at least 12 months of pre-study quality data to ensure ascertainment of new cases of HZ, had quality data for at least 12 months after inclusion, to ensure ascertainment of HZ complications (not reported here), and were eligible for linkage with HES APC and with IMD to ascertain the risk factors. Subjects were excluded if their date of vaccination was missing or they had indeterminate vaccination information, if they had received zoster vaccine prior to the year of vaccine introduction (i.e. January 1st, 2013), had a missing date of HZ, a PHN diagnosis without HZ diagnosis, or had the HZ diagnosis at the same date as the vaccination date. Subjects were followed continuously from study inclusion until first occurrence of HZ (as such, excluding recurrent events), death, patient transfer out of practice date, GP last data collection date or the end of the study (i.e. 31st December 2016).

### 2.3 Exposure- and outcome ascertainment

Vaccination status with Zostavax® was ascertained based on Read codes, positive immunization status in the CPRD immunization dataset or products codes (code list in **Supplementary Appendix A**). Subjects were considered vaccinated at the date of vaccination. HZ was defined as a first HZ event during the study period with the date of onset being the date of earliest evidence of HZ. Community-based HZ and PHN (exclusion criteria) cases were identified by Read codes and hospitalized cases by ICD-10 codes (code list in **Supplementary Appendix A**).

### 2.4 Factors affecting vaccine effectiveness

Lopalco and DeStefano have proposed that four types of host factors can affect VE, namely age, presence of conditions or co-morbidities that may either affect immune response or influence individual disease susceptibility, previous exposure to the antigen and interference due to co-administered vaccines or other drugs [13]. Conditions or co-morbidities that may influence susceptibility to HZ were identified based on a review of prior studies. The investigated risk factors for modified VE are summarized in Table 1.

**Table 1:** Overview of risk factors.

Risk factor				Description	Source
**(i) Age**					
		Age group (routine vs catch-up cohort)			[17], [18]

**(ii) Factors that may either affect immune response or influence individual disease susceptibility**					
	**Demographics**				
		Gender			[17], [18]
		Ethnicity (Caucasian vs non-Caucasian)		*Code list in Appendix A*	[17], [18]
		Socio-economic status (Index of Multiple deprivation (IMD); IMD < 5th quintile vs 5th quintile)			[17], [18]
	**Chronic conditions**				
		Asthma			[17], [19]
		Type 2 diabetes		*Code list in Appendix A*	[20]
		Chronic obstructive pulmonary disease (COPD)			[17], [21]
	**Life style factors**				
		Smoking (smoker, ex-smoker, non-smoker)		*Status was ascertained using the most recent information up to five years prior to study inclusion. Code list in Appendix A*	[22]
		Body Mass Index (BMI) (underweight, normal, overweight/obese)			[22]
	**Immunosuppression**				
		Immunocompromised conditions			
			-Acute and chronic leukaemias and lymphomas	*Treated as time-varying. Subjects considered immunocompromised from their first record of their immunocompromised condition onwards if they have any record of acute/chronic leukaemia and lymphomas, HIV/AIDS, cellular immune deficiencies or haematological malignancies. Patients are considered immunocompromised for 2 years after each record of haematopoietic stem cell transplant. Code list and details in Appendix A*	[23]
			-HIV/AIDS		[22]
			-Cellular immune deficiencies		[23]
			-Haematological malignancies		
			-Allogenic or autologous haematopoietic stem cell transplants		[23]
		Immunosuppressive or immunomodulating therapies			
			-Immunosuppressive chemotherapy or radiotherapy for malignant disease or non-malignant disorders	*Treated as time-varying. Patients are considered immunosuppressed when an immunosuppressive or immunomodulating therapy record is found until 3 months after each record. Code list and details in Appendix A*	[23]
			-Immunosuppressive therapy for a solid organ transplant		[23]
			-Biological therapy (e.g. anti-TNF therapy such as alemtuzumab, ofatumumab and rituximab)		[23]
			- Therapy with short term high-dose corticosteroids or long term lower dose corticosteroids or non-biological oral immune modulating drugs		

**(iii) Previous exposure to the antigen**					
		History of herpes zoster prior to study inclusion			[11]

**(iv) Interference due to co-administered vaccines or other drugs**					
		Co-administration with influenza vaccine		*Co-administration at the same day. Code list in Appendix A.*	[23]
		Co-administration with pneumococcal vaccine			[23]

### 2.5 Statistical analyses

Risk factors for modified VE were investigated using Poisson regression models. We first built the main effects model by simplifying the full model containing zoster vaccination status and all risk factors (Table 1) as main effects. Model simplification was done using the Chi-square likelihood ratio test at 5% significance level, dropping the least significant terms first until only significant terms remained, resulting in the simplified main effects model. Routine vs catch-up cohort (proxy for age), sex and exposure status were not considered for model exclusion. Then, to estimate differential VE in each risk group while accounting for differences in HZ baseline risk, interaction terms between exposure status and the risk factors were added one at a time to the simplified main effects model. In case the risk factor of interest was not included in the simplified main effects model, it was added to allow proper interpretation of the interaction term related to that risk factor. The HZ incidences (per 1000 person year) by HZ baseline risk factors and the overall VE were derived from the simplified main effects model. The relative increases/decreases in VE (in %) by risk group were derived from the simplified model with added interaction terms and the delta method was used to obtain 95% confidence intervals (CIs).

## 3 Results

**Fig. 1:**
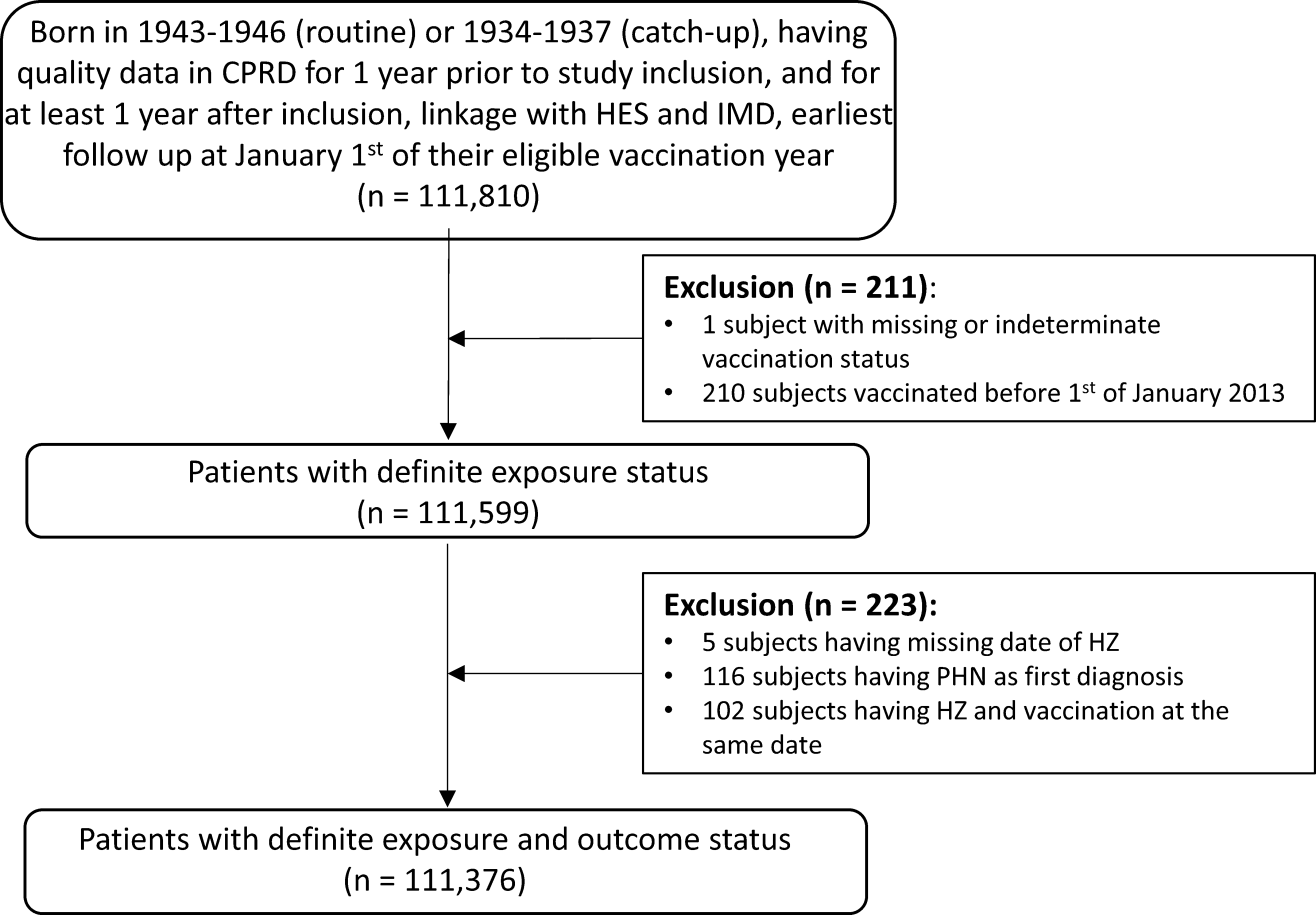
Attrition diagram.

In the linked dataset we identified quality-records of 111,810 subjects belonging to the routine or catch-up cohorts, of which 434 (0.39%) were excluded – initially 211 (0.19%) for indeterminate vaccination status or having been vaccinated before January 1st 2013, and a further 223 (0.20%) due to missing HZ data, diagnosis of PHN rather than HZ, or having HZ and vaccination on the same date (Fig. 1). This yielded a total of 111,376 subjects for analysis. The median follow-up time was 2.0 person-years, with an median follow-up time of 1.3 person-years post-vaccination.

**Fig. 2:**
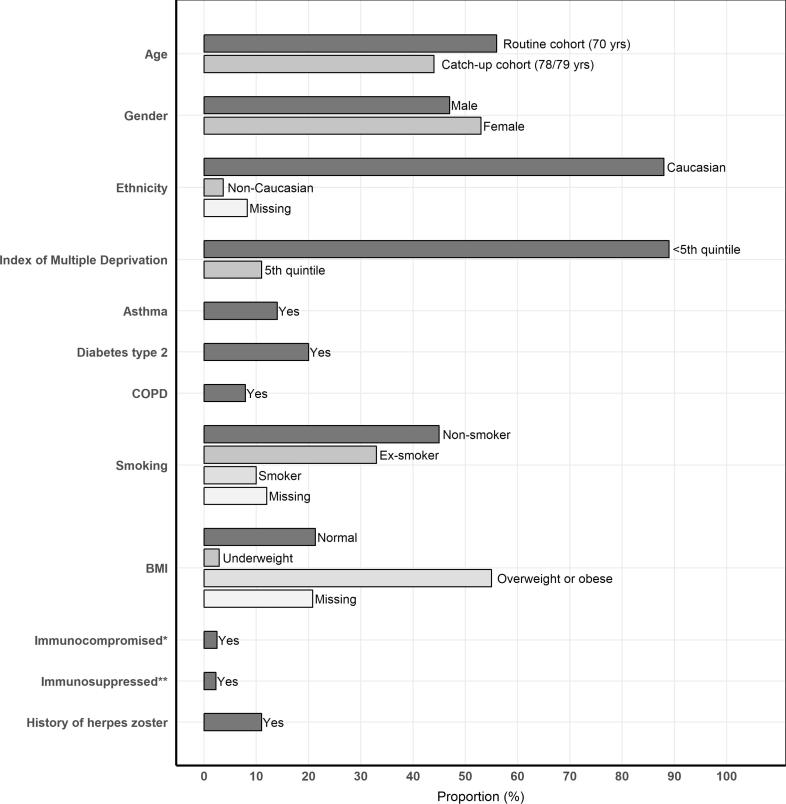
Proportions (%), by risk factor. *Immunocompromised conditions were evaluated at the end of subject’s follow-up. **Subjects were considered immunosuppressed when having at least one period of immunosuppression during their follow-up time.

The routine and catch-up cohorts combined accounted for a total of 143,122 and 112,597 person years, respectively (**Supplementary Appendix B**). Overall, the population was 88% Caucasian, 53% female, 45% non-smoking and 55% overweight or obese (i.e. having a BMI > 25 to < 30 or > 30, respectively). Approximately 14%, 20% and 7.9% of the population had asthma, type 2 diabetes and COPD, respectively (Fig. 2).

**Fig. 3:**
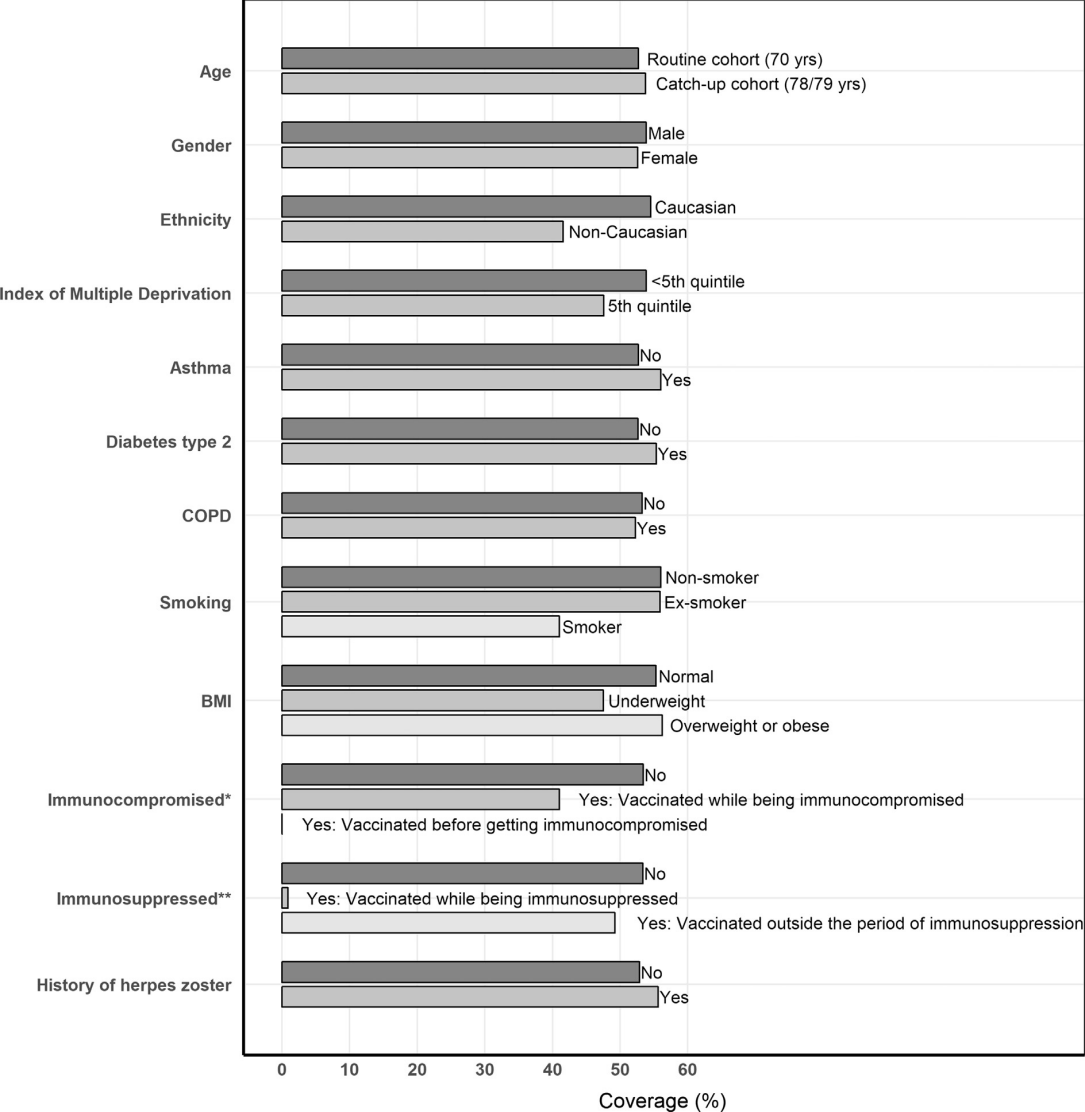
Herpes zoster coverage (%), evaluated 2 years after the cohort became eligible for vaccination, by risk factor. *Immunocompromised conditions were evaluated 2 years after the cohort became eligible for vaccination. **Subjects were considered immunosuppressed when having at least one period of immunosuppression during start of vaccination eligibility till 2 years later.

For each risk factor, we evaluated percentage vaccination coverage two years after the respective age cohort became eligible for vaccination as vaccine uptake increases rapidly the first year after vaccination eligibility and plateaus afterwards [11]. Vaccination coverage was slightly higher in the catch-up cohorts (53.7%) compared with the routine cohorts (52.7%) (Fig. 3). Non-Caucasians (41.5%), smokers (41.0%), subjects who were underweight (47.5%) and subjects with a low socio-economic status (47.6%) were vaccinated less. Vaccination was also less frequent in the immunocompromised (41.0%) or while subjects were immunosuppressed (0.88%) (Fig. 3). The zoster vaccine was often given concomitantly with influenza (35.8%), but rarely with the pneumococcal vaccine (1.3%).

The HZ incidence (per 1000 person years) in the general unvaccinated population was 9.34 (95% CI: 8.78, 9.90), assuming no other HZ risk factors were present. The main effects model showed a higher HZ incidence among females compared to males, Caucasians compared to non-Caucasians and among asthma patients, patients with a history of HZ, patients with immunocompromising conditions or who are immunosuppressed compared to the overall population (Table 2). The estimated overall VE against HZ was 66.8% (95% CI: 62.2, 71.0).

**Table 2:** Herpes zoster incidence in unvaccinated populations (per 1000 person-years).

Risk factor		Herpes zoster incidence (/1000 person-years) [95% CI]^a^
**General population**		
	Overall	9·34 [8·78; 9·90]
	Routine cohort	9·45 [8·72; 10·18]
	Catch-up cohort	9·20 [8·55; 9·85]
	Female	10·55 [9·80; 11·3]
	Male	8·14 [7·49; 8·79]
	Caucasian	9·53 [8·95; 10·10]
	Non-Caucasian	5·79 [4·15; 7·43]
	Non-smoker	9·66 [8·94; 10·39]
	Ex-smoker	9·36 [8·54; 10·17]
	Smoker	8·01 [6·83; 9·20]

**Patient populations**		
	Asthma	11·20 [9·94; 12·45]
	Immunocompromised	15·48 [12·17; 18·79]
	Immunosuppressed	14·23 [10·92; 17·55]
	History of herpes zoster	10·78 [9·39; 12·17]

a Derived from simplified main effects model, assuming age, gender and ethnicity distribution as well as smoking behaviour reflective of the UK population. Assuming no other herpes zoster risk factors are present.

Type 2 diabetes and history of HZ differentially affected the VE with a relative difference in VE of −22.2% for diabetics compared to non-diabetics (95% CI: −39.6, −4.5) and a relative difference of −22.5% for subjects with compared to subjects without a history of HZ (95% CI: −44.9, −0.1) (Table 3).

**Table 3:** Risk factors for modified vaccine effectiveness.

Risk factor		Number of vaccinated cases	Person-years of vaccinated subjects	Number of unvaccinated cases	Person-years of unvaccinated subjects	Vaccine effectiveness (95% CI)	Relative difference, % (95% CI)
**Age**							
	Routine cohort (70 yrs)	130	42,121	952	101,001	0·68 [0·62; 0·74]	Ref^a^
	Catch-up cohort (78–79 yrs)	123	35,369	773	77,228	0·65 [0·59; 0·72]	−3·6 [−16·4; 9·1]

**Gender**							
	Male	100	36,733	698	83,494	0·68 [0·61; 0·75]	Ref
	Female	153	40,757	1027	94,734	0·66 [0·60; 0·72]	−3·3 [−16·0; 9·5]

**Ethnicity**							
	Caucasian	234	70,452	1590	155,707	0·67 [0·63; 0·72]	Ref
	Non-Caucasian	10	2474	39	6993	0·27 [−0·23; 0·78]	−59.3 [−134·0; 15·5]

**Index of Multiple Deprivation**							
	< 5th quintile	229	69,798	1553	157,422	0·67 [0·63; 0·72]	Ref
	5th quintile	24	7674	171	20,744	0·63 [0·47; 0·79]	−6·0 [−30·3; 18·2]

**Asthma**							
	No	209	65,970	1427	153,096	0·67 [0·62; 0·71]	Ref
	Yes	44	11,520	298	25,132	0·68 [0·58; 0·78]	1.8 [−15·2; 18·8]

**Type 2 diabetes**							
	No	183	61,470	1395	143,769	0·70 [0·65; 0·75]	Ref
	Yes	70	16,020	330	34,460	0·55 [0·43; 0·66]	−**22·0 [**−**39·6;** −**4·5]**

**Chronic obstructive pulmonary disease (COPD)**							
	No	232	71,632	1569	163,994	0·67 [0·62; 0·71]	Ref
	Yes	21	5858	156	14,234	0·67 [0·52; 0·82]	0.8 [−22·6; 24·2]

**Smoking**							
	Non-smoker	133	36,757	800	77,434	0·65 [0·59; 0·71]	Ref
	Ex-smoker	83	26,604	567	57,061	0·68 [0·61; 0·76]	5·0 [−10·2; 20·3]
	Smoker	20	5984	167	20,368	0·60 [0·41; 0·78]	−8.4 [−38·5; 21·8]

**Body mass index (BMI)**							
	Normal weight (18·5– 24.9)	56	17,309	362	37,062	0·67 [0·58; 0·76]	Ref
	Underweight (<18·5)	4	1973	59	5476	0·82 [0·63; 1·00]	21·7 [−10·9; 54·4]
	Overweight (25–29.9) or obese (>30)	159	45,083	962	95,361	0·65 [0·59; 0·71]	−2·8 [−18·8; 13·3]

**Immunocompromised**							
	No	241	75,992	1649	173,438	0·67 [0·63; 0·72]	Ref
	Yes	12	1498	76	4791	0·49 [0·19; 0·80]	−26·8 [−72·8; 19·3]

**Immunosuppressed**							
	No	245	75,948	1659	173,949	0·67 [0·62; 0·71]	Ref
	Yes	8	1542	66	4280	0·66 [0·41; 0·91]	−1·1 [−38·9; 36·7]

**History of herpes zoster**							
	No	208	68,635	1512	158,845	0·69 [0·64; 0·73]	Ref
	Yes	45	8855	213	19,384	0·53 [0·38; 0·68]	−**22·5 [**−**44·9;** −**0·1]**

**Co-administration with influenza vaccine**							
	No	164	48,708	1725	178,229	0·66 [0·60; 0·71]	Ref
	Yes	89	28,782	–	–	0·68 [0·62; 0·75]	4·0 [−8·9; 17·0]

**Co-administration with pneumococcal vaccine**							
	No	251	76,567	1725	178,229	0·67 [0·62; 0·71]	Ref
	Yes	2	923	–	–	0·77 [0·45; 1·00]	15·2 [−33·5; 63·9]

a Ref = reference group.

## 4 Discussion

In this retrospective database study, we found that in adults ≥ 70 years of age the live-attenuated HZ vaccine had an overall VE against herpes zoster of 66.8% (95% CI: 62.2, 71.0). However, we found that this VE was not equal in all population groups. More specifically, we found that diabetics and persons who previously experienced HZ are less well protected by the vaccine compared with persons who do not have such risk factors. These two population groups combined represent 28.5% of the entire population for whom the vaccine is recommended. The relative difference in VE was −22.2% (95% CI: −39.6, −4.5) for diabetics compared to non-diabetics and −22.5% (95% CI: −44.9, −0.1) for subjects with compared to subjects without a history of HZ.

The effect of diabetes on vaccination has been studied for some vaccines such as Hepatitis B and influenza vaccines. Several studies have shown a decreased seroconversion rate following hepatitis B vaccination in diabetics [24], [25]. Relatively few studies have assessed the effect of diabetes on VE in the real-world setting. One recent study found a significantly increased effectiveness of the conjugated pneumococcal vaccine against vaccine-type community-acquired pneumonia when administered to elderly diabetics in a large randomized trial in the Netherlands [26]. However, in studies of other vaccinations among diabetics, mostly influenza vaccines, no clear impact was seen on immunogenicity [27], or on VE [28], [29]. Surprisingly we could not replicate the expected increased risk of HZ among the diabetics in our study [20]. Further analyses may be needed to analyse whether disease severity or use of medication may be associated with the decreased VE observed in our study.

The lower VE we found in subjects with a history of HZ was also found in the recent study on the first three years of the UK programme; VE was 47% (95% CI; 31, 58%) in those with prior zoster versus 64% (95% CI; 60, 68%) in those without previous zoster [12]. The authors postulated that this finding might be explained by a differential uptake among those with a relatively recent zoster episode, whereby persons with a recent zoster (temporarily protected against a new episode) would be less likely to be vaccinated compared with subjects with a less recent zoster (less protected as a result of waning immunity) [12]. We could not confirm this hypothesis as we found that the vaccination uptake was comparable in those with a recent (≤5 years) and less recent (>5 years) HZ episode prior to vaccination eligibility (32.4% and 30.7%).

Our estimated overall VE against HZ of 66.8% (95% CI: 62.2, 71.0) is higher than the estimates found in a clinical trial (56%; 95% CI: 40, 67) [5], but is comparable with values found in other observational studies [8]. This is probably explained by differences in length of follow-up after vaccination and differences in captured cases (mild cases are more likely to be captured by the active surveillance in clinical trials compared with healthcare database studies) and the finding that the vaccine offers higher protection against more severe zoster [5], [8]. Contrary to the clinical trial results, our results did not show differences in overall VE by age. Absence of an age effect was also observed in previous observational studies on zoster VE [8], [12], [30].

The VE against zoster was also not affected by co-administration with the influenza vaccine. In the UK, General Practitioners were encouraged to administer zoster concomitantly with inactivated influenza vaccine [23]. The zoster vaccine can be co-administered with the 23-valent pneumococcal polysaccharide vaccine although a single study had shown an inferior VZV antibody response in those receiving both vaccines concomitantly [31]. We found that 35.8% of the zoster vaccines was given on the same day as the influenza vaccine but only 1.3% was given on the same day as the pneumococcal vaccine.

Although at increased HZ risk (Table 2), patients with immunocompromised conditions or those on immunosuppressive or immuno-modulating therapy are contra-indicated for zoster vaccination, or at least the vaccine should be used cautiously [23]. We found that the recommendation not to vaccinate subjects on immunosuppressive therapy was well followed by medical practitioners, with the low number of vaccinations (0.9%) in these groups of patients possibly explained by misclassification. We did not find any evidence that immunosuppressed subjects were less protected by the vaccine. Of the 2.5% of the population that was classified as immunocompromised, 41.0% was still vaccinated. Although as expected, the HZ baseline incidence was higher in the immunocompromised (Table 2) compared with the immunocompetent free of HZ risk factors, the high vaccination coverage in immunocompromised subjects is unexpected. This finding might be explained by coding inconsistencies [6]. Such a high vaccination coverage in immunocompromised subjects was also recently found in another study [32].

While we also found the zoster VE to be unaffected by age, gender, ethnicity, socio-economic status, asthma, COPD, BMI or smoking, the absence of any significant differences, especially when the risk factor is less frequent, should be treated cautiously as it might be explained by a lack of statistical power. Additional limitations of our study that apply to both the significant and non-significant findings include the potential misclassification of exposure, outcome and risk factors. As the zoster vaccine is administered at General Practices, the exposure misclassification is expected to be limited. The same holds for outcome misclassification with the misclassification of the medically attended HZ being unlikely as HZ is a largely clinical diagnosis. Misclassification seems more likely for some of the risk factors. A substantial amount of missing information was observed for ethnicity (8.3%), BMI (21.1%) and smoking (12.0%). The index of multiple deprivation is a variable at small area level, not at the individual level, possibly introducing ecological bias. Despite our attempts to minimize misclassification of chronic (asthma, COPD, type 2 diabetes) and immunocompromised conditions by linking to hospital data, misclassification can still arise as a result of incomplete medical histories or coding inconsistencies.

In spite these limitations, we believe that healthcare databases are a good source for the study of risk factors for modified VE due to their large size. In our database study, we found that a large proportion of the population, specifically those with diabetes and prior history of HZ, benefit less from the live-attenuated HZ vaccine compared with the rest of the population. Age, gender, ethnicity, socio-economic status, asthma, COPD, smoking, BMI, immunosuppression and co-administration with influenza and with pneumococcal vaccine were not found to modify the VE. Our study illustrates the importance of assessing VE by subpopulations. Identifying and understanding the effect modifiers of VE is important for future vaccine development as well as vaccine recommendations. Our findings might be particularly relevant in the light of the likely licensure of the new zoster adjuvanted recombinant vaccine Shingrix® in the UK. Unlike the live-attenuated zoster vaccine Zostavax®, the recombinant zoster vaccine is also indicated for use among immunocompromised patients. Given in a 2-dose series, the new vaccine showed an efficacy at preventing zoster of at least 90% in clinical trials with efficacy above 85% for up to four years after vaccination [33], [34]. The real-world effectiveness of the new recombinant vaccine still needs to be demonstrated and it is recommended to also evaluate potential VE effect modifiers of the new vaccine in order to formulate optimal vaccine recommendations.

## 5 Author’s contributions

KB and TV designed the study. MA did the data pre-processing and presentation of results. KB performed the statistical analyses and wrote the first draft and subsequent revisions. All authors reviewed and approved the final version before submission.
