# Supplementary material for: Risk factors for modified vaccine effectiveness of the live attenuated zoster vaccine among the elderly in England
Source: Vaccine X. 2019 Jan 29;1:100007. doi: 10.1016/j.jvacx.2019.100007 (PMC6668231; doi:10.1016/j.jvacx.2019.100007)
Supplement: Supplementary data 1 [file mmc1.docx]

# Appendix A: Code lists

# Exposure

## HZ Vaccination status

### HZ Vaccination Medcodes

| **Medcode** | **Description** |
| --- | --- |
| 106904 | Herpes zoster vaccination |
| 106593 | Shingles vaccination |
| 107067 | Herpes zoster vaccination given by other health care provide |
| 106948 | No consent for herpes zoster vaccination |
| 106946 | Herpes zoster vaccination contraindicated |
| 106947 | Herpes zoster vaccination declined |
| 107061 | Did not attend herpes zoster vaccination |
| 108895 | [X]Herpes zoster vacc caus adverse effects therapeutic use |

### HZ Vaccination Product codes

| **Prodcode** | **Description** |
| --- | --- |
| 47327 | Zostavax vaccine powder and solvent for suspension for injection 0.65ml pre-filled syringes (sanofi pasteur MSD Ltd); (Varicella Zoster Virus, Live (oka/merck Strain)) |
| 48314 | Shingles (Herpes Zoster) vaccine (live) powder and solvent for suspension for injection 0.65ml pre-filled syringes; () |

### HZ Vaccination Immunisation types

| **Immunisation Type** | **Description** |
| --- | --- |
| 88 | SHINGLES |
| 91 | SHINGLESOHP |

## Flu vaccination status

### Flu Vaccination Medcodes

| **Medcode** | **Description** |
| --- | --- |
| 6 | Influenza vaccination |
| 94301 | First pandemic influenza vaccination |
| 107315 | Administration of first intranasal pandemic influenza vacc |
| 95092 | Second pandemic influenza vaccination |
| 107646 | Administration of second intranasal pandemic influenza vacc |
| 97941 | Influenza vaccination given by other healthcare provider |
| 105077 | Seasonal influenza vaccin given by other healthcare provider |
| 107413 | First intranasal seasonal flu vacc gvn by othr hlthcare prov |
| 107730 | Secnd intranasal seasonal flu vacc gvn by othr hlthcare prov |
| 98217 | 1st pandemic influenza vacc give by other healthcare providr |
| 108772 | First intranasal pndmc influenza vcc gvn othr hlthcare prvdr |
| 98306 | 2nd pandemic influenza vacc give by other healthcare providr |
| 98234 | CELVAPAN - first influenza A (H1N1v) 2009 vaccination given |
| 98302 | CELVAPAN - second influenza A (H1N1v) 2009 vaccination given |
| 98449 | CELVAPAN - 1st flu A (H1N1v) 2009 vacc by othr hlth provider |
| 98303 | CELVAPAN - 2nd flu A (H1N1v) 2009 vacc by othr hlth provider |
| 98183 | PANDEMRIX - first influenza A (H1N1v) 2009 vaccination given |
| 98184 | PANDEMRIX - second influenza A (H1N1v) 2009 vaccination give |
| 98203 | PANDEMRIX - 1st flu A (H1N1v) 2009 vac by othr hlth provider |
| 98304 | PANDEMRIX - 2nd flu A (H1N1v) 2009 vac by othr hlth provider |
| 104688 | Seasonal influenza vaccination |
| 105195 | Seasonal influenza vaccination given by pharmacist |
| 107297 | Administration of first intranasal seasonal influenza vacc |
| 107573 | Seasonal influenza vaccination given while hospital inpt |
| 107352 | Administration of second intranasal seasonal influenza vacc |
| 107156 | Administration of intranasal influenza vaccination |
| 106994 | Administration of first intranasal influenza vaccination |
| 106995 | Administration of second intranasal influenza vaccination |
| 9039 | Influenza vacc. administratn. |
| 12104 | Flu vaccination administration |
| 18330 | Has 'flu vaccination at home |
| 44555 | Has'flu vaccination at surgery |
| 18684 | Has 'flu vaccination at hosp. |
| 32942 | Has influenza vaccination at work |
| 35655 | Influenza vacc.administrat.NOS |
| 12336 | [V]Influenza vaccination |
| 21123 | [V]Flu - influenza vaccination |

### Flu Vaccination Product codes

| **Prodcode** | **Description** |
| --- | --- |
| 12565 | Vaccine administered |
| 30156 | Vaccine administered |
| 7951 | Vaccine administered |
| 61580 | Vaccine administered |
| 17795 | Vaccine administered |
| 19684 | Vaccine administered |
| 47932 | Vaccine administered |
| 51289 | Vaccine administered |
| 1641 | Vaccine administered |
| 18612 | Vaccine administered |
| 54677 | Vaccine administered |
| 43827 | Vaccine administered |
| 2601 | Vaccine administered |
| 13595 | Vaccine administered |
| 398 | Vaccine administered |
| 1329 | Vaccine administered |
| 38421 | Vaccine administered |
| 40876 | Vaccine administered |
| 2139 | Vaccine administered |
| 24779 | Vaccine administered |
| 57917 | Vaccine administered |
| 2552 | Vaccine administered |
| 639 | Vaccine administered |
| 61898 | Vaccine administered |
| 41925 | Vaccine administered |
| 45661 | Vaccine administered |
| 922 | Vaccine administered |
| 9710 | Vaccine administered |
| 57678 | Vaccine administered |
| 48658 | Vaccine administered |
| 43825 | Vaccine administered |
| 11824 | Vaccine administered |
| 16585 | Vaccine administered |
| 48740 | Vaccine administered |
| 51087 | Vaccine administered |
| 10030 | Vaccine administered |
| 41168 | Vaccine administered |
| 32391 | Vaccine administered |
| 30198 | Vaccine administered |
| 61792 | Vaccine administered |
| 48085 | Vaccine administered |
| 57401 | Vaccine administered |
| 57140 | Vaccine administered |
| 41240 | Vaccine administered |
| 834 | Vaccine administered |
| 41150 | Vaccine administered |
| 27407 | Vaccine administered |
| 40760 | Vaccine administered |
| 49716 | Vaccine administered |

### Flu Vaccination Immunisation type

| **Immunisation type** | **Description** |
| --- | --- |
| 4 | FLU |
| 71 | PFLUGEN |
| 72 | PFLUGSK |
| 73 | PFLUGSKO |
| 74 | PFLUGS |
| 75 | PFLUBAXO |
| 76 | PFLUBAX |
| 78 | PFLUGENO |
| 84 | FLUSOHP |
| 85 | FLUSPHARMA |
| 89 | FLUSIN |
| 97 | FLUSINOHP |
| 100 | FLUSIMOHP |

# HZ and PHN codes

The code lists are based on the code lists in Forbes, H. J. *Understanding risk factors for herpes zoster and postherpetic neuralgia in UK primary care: investigations to inform vaccine policy*. Diss. London School of Hygiene & Tropical Medicine, 2016.

## HZ

### HZ Medcodes

| **Medcode** | **Description** |
| --- | --- |
| 390 | Herpes zoster |
| 516 | Shingles |
| 7331 | Ramsay - Hunt syndrome |
| 8936 | Ophthalmic herpes zoster infection |
| 14718 | Herpes zoster with ophthalmic complication |
| 14793 | Herpes zoster otitis externa |
| 18918 | Herpes zoster ophthalmicus |
| 21069 | Herpes zoster with unspecified complication |
| 21471 | Herpes zoster NOS |
| 25320 | Herpes zoster with dermatitis of eyelid |
| 27403 | Geniculate herpes zoster |
| 27546 | Herpes zoster with keratoconjunctivitis |
| 31681 | Herpes zoster - otitis externa |
| 33810 | Herpes zoster with other ophthalmic complication |
| 38531 | Herpes zoster with other specified complication NOS |
| 39692 | Polyneuropathy in herpes zoster |
| 43235 | Herpes zoster with other specified complication |
| 44944 | Herpes zoster with meningitis |
| 47375 | Zoster encephalitis |
| 50537 | Herpes zoster with other CNS complications |
| 51692 | Encephalitis due to herpes zoster |
| 52126 | Herpes zoster with other central nervous system complication |
| 52319 | Disseminated zoster |
| 55940 | Herpes zoster iridocyclitis |
| 57895 | Herpes zoster meningitis |
| 62558 | Infective otitis externa due to herpes zoster |
| 63739 | Herpes zoster with other CNS complication NOS |
| 69405 | Herpes zoster encephalitis |
| 70197 | [X]Zoster without complications |
| 71464 | Meningitis due to herpes zoster virus |
| 105157 | Hutchinson's sign - herpes zoster involving nose tip |

### HZ ICD-10 codes

| **ICD-10** | **Description** |
| --- | --- |
| B02 | Zoster |
| B02.0 | Zoster encephalitis |
| B02.1 | Zoster meningitis |
| B02.3 | Zoster ocular disease |
| B02.7 | Disseminated zoster |
| B02.8 | Otitis externa due to herpes zoster |
| B02.9 | Zoster with other complications |

## PHN

### PHN Medcodes

| **Medcode** | **Description** |
| --- | --- |
| 1598 | Post-herpetic neuralgia |
| 7584 | Post-herpetic trigeminal neuralgia |
| 10223 | Postherpetic neuralgia |
| 11498 | Postherpetic trigeminal neuralgia |
| 17180 | Postzoster neuralgia |
| 31709 | Postherpetic polyneuropathy |

### PHN ICD-10 codes

| **ICD-10** | **Description** |
| --- | --- |
| B02.2 | Zoster with other nervous system involvement |
| B02.21 | Postherpetic geniculate ganglionitis |
| B02.22 | Postherpetic trigeminal neuralgia |
| B02.23 | Postherpetic polyneuropathy |
| B02.24 | Postherpetic myelitis |
| B02.29 | Other postherpetic nervous system involvement |

# Demographics

## Ethnicity

The code list is based on Mathur, Rohini, et al. "Completeness and usability of ethnicity data in UK-based primary care and hospital databases." *Journal of public health* 36.4 (2013): 684-692.

If HES APC ethnicity information is available, this was used to classify the Ethnicity class in the analyses. If HES APC ethnicity information is not available, the informative ethnicity value from CPRD Clinical available for the latest date will be used to classify the ethnicity class in the analyses. If in CPRD Clinical file contradictory information is reported at the same date or no information is recorded ethnicity will be considered as “Not stated” for the subject.

### Ethnicity Medcodes

The table below includes all the medcodes used in CPRD to identify the ethnicity.

| **medcode** | **Description** | **Ethnicity** | **Caucasian** |
| --- | --- | --- | --- |
| 32778 | Cypriot (part not stated) - ethnic category 2001 census | White | Caucasian |
| 10196 | Ethnic groups (census) | Not stated | Non caucasian |
| 46063 | Jewish - ethnic category 2001 census | Other ethnic groups | Non caucasian |
| 32136 | Other black ethnic group | Black | Non caucasian |
| 110696 | Mixed/multiple ethnic grps: any- Scot ethnic cat 2011 census | Mixed | Non caucasian |
| 39696 | Indian sub-continent (NMO) | Asian or Asian British | Non caucasian |
| 24837 | White Irish | White | Caucasian |
| 12350 | African - ethnic category 2001 census | Black | Non caucasian |
| 32420 | Other ethnic, other mixed orig | Mixed | Non caucasian |
| 12769 | Greek Cypriot - ethnic category 2001 census | White | Caucasian |
| 47005 | Asian and Chinese - ethnic category 2001 census | Mixed | Non caucasian |
| 111059 | African: African/African Scot/African Brit - Scotland 2011 | Black | Non caucasian |
| 110779 | Black/Afri/Carib/Black Brit: Caribbean- NI eth cat 2011 cens | Black | Non caucasian |
| 12437 | White and Black African - ethnic category 2001 census | Mixed | Non caucasian |
| 46059 | Arab - ethnic category 2001 census | Other ethnic groups | Non caucasian |
| 110436 | Black/African/Caribbn/Black Brit: Caribbean - Eng+Wales 2011 | Black | Non caucasian |
| 28935 | Other Asian or Asian unspecified ethnic category 2001 census | Asian or Asian British | Non caucasian |
| 25422 | Albanian - ethnic category 2001 census | White | Caucasian |
| 32132 | O/E - Asian origin | Asian or Asian British | Non caucasian |
| 12591 | Other White or White unspecified ethnic category 2001 census | White | Caucasian |
| 12355 | Greek - ethnic category 2001 census | White | Caucasian |
| 32401 | Other ethnic, Asian/White orig | Mixed | Non caucasian |
| 12443 | Somali - ethnic category 2001 census | Black | Non caucasian |
| 110422 | Asian or Asian British: Indian - NI ethnic cat 2011 census | Asian or Asian British | Non caucasian |
| 12742 | White and Black Caribbean - ethnic category 2001 census | Mixed | Non caucasian |
| 110661 | Mixed: White and Black Caribbean - NI ethnic cat 2011 census | Mixed | Non caucasian |
| 41214 | Other ethnic NEC (NMO) | Other ethnic groups | Non caucasian |
| 110687 | White: Irish - Scotland ethnic category 2011 census | White | Caucasian |
| 32066 | Turkish/Turkish Cypriot (NMO) | Other ethnic groups | Non caucasian |
| 46056 | Mixed Asian - ethnic category 2001 census | Asian or Asian British | Non caucasian |
| 110651 | Mixed: White and Black African - NI ethnic cat 2011 census | Mixed | Non caucasian |
| 66536 | O/E - Mongoloid origin | Not stated | Non caucasian |
| 32069 | Turkish Cypriot (NMO) | Other ethnic groups | Non caucasian |
| 110855 | Asian: other Asian group - Scotland ethnic cat 2011 census | Asian or Asian British | Non caucasian |
| 12730 | Malaysian - ethnic category 2001 census | Other ethnic groups | Non caucasian |
| 47969 | Other African countries (NMO) | Black | Non caucasian |
| 56127 | Hindu - ethnic category 2001 census | Asian or Asian British | Non caucasian |
| 110477 | Asian/Asian Brit: Indian - Eng+Wales ethnic cat 2011 census | Asian or Asian British | Non caucasian |
| 12468 | Chinese - ethnic category 2001 census | Other ethnic groups | Non caucasian |
| 47077 | East African Asian - ethnic category 2001 census | Mixed | Non caucasian |
| 28866 | Croatian - ethnic category 2001 census | White | Caucasian |
| 110590 | Asian/Asian Brit: Bangladeshi- Eng+Wales eth cat 2011 census | Asian or Asian British | Non caucasian |
| 93749 | Patient ethnicity unknown | Not stated | Non caucasian |
| 47401 | Other ethnic, Black/White orig | Mixed | Non caucasian |
| 32126 | Turkish (NMO) | Other ethnic groups | Non caucasian |
| 12352 | English - ethnic category 2001 census | White | Caucasian |
| 110780 | Other ethnic group: Arab - NI ethnic category 2011 census | Other ethnic groups | Non caucasian |
| 30280 | Other ethnic non-mixed (NMO) | Other ethnic groups | Non caucasian |
| 110630 | Black/Afri/Carib/Black Brit: African- NI eth cat 2011 census | Black | Non caucasian |
| 55113 | Traveller - ethnic category 2001 census | White | Caucasian |
| 85505 | Other European in New Zealand | White | Caucasian |
| 12887 | Sinhalese - ethnic category 2001 census | Asian or Asian British | Non caucasian |
| 42290 | Gypsy/Romany - ethnic category 2001 census | White | Caucasian |
| 28936 | Other republics former Yugoslavia - ethnic categ 2001 census | White | Caucasian |
| 49658 | Sikh - ethnic category 2001 census | Asian or Asian British | Non caucasian |
| 25937 | Iranian - ethnic category 2001 census | Other ethnic groups | Non caucasian |
| 110421 | Mixed: White+Black African - Eng+Wales eth cat 2011 census | Mixed | Non caucasian |
| 12608 | Sri Lankan - ethnic category 2001 census | Asian or Asian British | Non caucasian |
| 71425 | New Zealand ethnic group NOS | Other ethnic groups | Non caucasian |
| 32382 | Mauritian/Seychellois/Maldivian/St Helena eth cat 2001census | Other ethnic groups | Non caucasian |
| 110694 | White: other British - Scotland ethnic category 2011 census | White | Caucasian |
| 96789 | Other New Zealand ethnic group | Not stated | Non caucasian |
| 46818 | East African Asian (NMO) | Mixed | Non caucasian |
| 35459 | Other ethnic, mixed white orig | Mixed | Non caucasian |
| 12429 | Ethnic group not given - patient refused | Not stated | Non caucasian |
| 110471 | Mixed: White and Asian - NI ethnic category 2011 census | Mixed | Non caucasian |
| 12432 | Caribbean - ethnic category 2001 census | Black | Non caucasian |
| 40110 | Black and White - ethnic category 2001 census | Mixed | Non caucasian |
| 12420 | Filipino - ethnic category 2001 census | Other ethnic groups | Non caucasian |
| 12467 | Polish - ethnic category 2001 census | White | Caucasian |
| 32399 | Caribbean Asian - ethnic category 2001 census | Asian or Asian British | Non caucasian |
| 26310 | Other white British ethnic group | White | Caucasian |
| 12760 | Tamil - ethnic category 2001 census | Asian or Asian British | Non caucasian |
| 38097 | E Afric Asian/Indo-Carib (NMO) | Asian or Asian British | Non caucasian |
| 12414 | Indian or British Indian - ethnic category 2001 census | Asian or Asian British | Non caucasian |
| 12459 | Ethnic category not stated - 2001 census | Not stated | Non caucasian |
| 12412 | Italian - ethnic category 2001 census | White | Caucasian |
| 12756 | South and Central American - ethnic category 2001 census | Other ethnic groups | Non caucasian |
| 110742 | Other ethnic: any other grp - Eng+Wales eth cat 2011 census | Other ethnic groups | Non caucasian |
| 12873 | Other Mixed background - ethnic category 2001 census | Mixed | Non caucasian |
| 32389 | Other Black background - ethnic category 2001 census | Black | Non caucasian |
| 110417 | Ethnic category - 2011 census England and Wales | Not stated | Non caucasian |
| 32443 | Black African and White | Mixed | Non caucasian |
| 110646 | Other ethnic group: any other grp- NI ethnic cat 2011 census | Other ethnic groups | Non caucasian |
| 12436 | Scottish - ethnic category 2001 census | White | Caucasian |
| 12351 | British or mixed British - ethnic category 2001 census | White | Caucasian |
| 12653 | British Asian - ethnic category 2001 census | Asian or Asian British | Non caucasian |
| 110460 | Asian: Pakistani/Pakistani Scot/Pakistani Brit- Scot 2011 | Asian or Asian British | Non caucasian |
| 110922 | Asian/Asian Brit: Chinese - Eng+Wales ethnic cat 2011 census | Asian or Asian British | Non caucasian |
| 12668 | Other Asian ethnic group | Asian or Asian British | Non caucasian |
| 12746 | Turkish - ethnic category 2001 census | White | Caucasian |
| 22467 | White | White | Caucasian |
| 28900 | Other mixed White - ethnic category 2001 census | White | Caucasian |
| 12706 | Chinese and White - ethnic category 2001 census | Mixed | Non caucasian |
| 32408 | Other Mixed or Mixed unspecified ethnic category 2001 census | Mixed | Non caucasian |
| 24340 | Ethnic group not recorded | Not stated | Non caucasian |
| 25969 | O/E - ethnic group | Not stated | Non caucasian |
| 12460 | Pakistani or British Pakistani - ethnic category 2001 census | Asian or Asian British | Non caucasian |
| 47285 | North African Arab (NMO) | Other ethnic groups | Non caucasian |
| 26246 | Latin American - ethnic category 2001 census | Other ethnic groups | Non caucasian |
| 110556 | White: Irish - England and Wales ethnic category 2011 census | White | Caucasian |
| 98213 | White Irish - ethnic category 2001 census | White | Caucasian |
| 32396 | Other Asian | Asian or Asian British | Non caucasian |
| 49940 | Black and Chinese - ethnic category 2001 census | Mixed | Non caucasian |
| 57764 | Brit. ethnic minor. unsp (NMO) | Not stated | Non caucasian |
| 110432 | White: Scottish - Scotland ethnic category 2011 census | White | Caucasian |
| 32425 | Black Caribbean and White | Mixed | Non caucasian |
| 12532 | Irish - ethnic category 2001 census | White | Caucasian |
| 110720 | Asian/Asian British: Bangladeshi - NI ethnic cat 2011 census | Asian or Asian British | Non caucasian |
| 47091 | Muslim - ethnic category 2001 census | Other ethnic groups | Non caucasian |
| 63872 | Buddhist - ethnic category 2001 census | Other ethnic groups | Non caucasian |
| 24962 | N African Arab/Iranian (NMO) | Other ethnic groups | Non caucasian |
| 45199 | Ethnic groups (census) NOS | Not stated | Non caucasian |
| 40097 | Black British - ethnic category 2001 census | Black | Non caucasian |
| 12795 | Black and Asian - ethnic category 2001 census | Mixed | Non caucasian |
| 28973 | Commonwealth (Russian) Indep States - ethn categ 2001 census | White | Caucasian |
| 32886 | Nigerian - ethnic category 2001 census | Black | Non caucasian |
| 26455 | Any other group - ethnic category 2001 census | Other ethnic groups | Non caucasian |
| 46752 | Other Pacific ethnic group | Other ethnic groups | Non caucasian |
| 40102 | Ulster Scots - ethnic category 2001 census | Not stated | Non caucasian |
| 28887 | Cornish - ethnic category 2001 census | Not stated | Non caucasian |
| 12638 | White and Asian - ethnic category 2001 census | Mixed | Non caucasian |
| 26392 | Punjabi - ethnic category 2001 census | Asian or Asian British | Non caucasian |
| 110540 | Black/Afr/Carib/Black Brit: other Black- Eng+Wales 2011 cens | Black | Non caucasian |
| 110655 | African: any other African - Scotland ethnic cat 2011 census | Black | Non caucasian |
| 110425 | Asian/Asian British: other Asian - NI ethnic cat 2011 census | Asian or Asian British | Non caucasian |
| 24270 | Irish (NMO) | White | Caucasian |
| 45008 | New Zealand ethnic groups | Other ethnic groups | Non caucasian |
| 110654 | Mixed: other Mixed/multiple backgrd - Eng+Wales 2011 census | Mixed | Non caucasian |
| 25920 | Indian | Asian or Asian British | Non caucasian |
| 26391 | Mixed Irish and other White - ethnic category 2001 census | White | Caucasian |
| 110407 | White: other White backgrd- Eng+Wales ethnic cat 2011 census | White | Caucasian |
| 32781 | Traveller - gypsy | Other ethnic groups | Non caucasian |
| 60284 | O/E - ethnic group NOS | Not stated | Non caucasian |
| 46956 | Bosnian - ethnic category 2001 census | White | Caucasian |
| 110652 | Mixed: White+Asian - Eng+Wales ethnic category 2011 census | Mixed | Non caucasian |
| 28909 | Mid East (excl Israeli, Iranian & Arab) - eth cat 2001 cens | Other ethnic groups | Non caucasian |
| 12332 | O/E - ethnic origin | Not stated | Non caucasian |
| 64133 | Kashmiri - ethnic category 2001 census | Asian or Asian British | Non caucasian |
| 26467 | White Scottish | White | Caucasian |
| 12444 | Other white ethnic group | White | Caucasian |
| 47074 | Serbian - ethnic category 2001 census | White | Caucasian |
| 12433 | Baltic Estonian/Latvian/Lithuanian - ethn categ 2001 census | White | Caucasian |
| 24404 | O/E - Negroid | Not stated | Non caucasian |
| 32110 | Brit. ethnic minor. spec.(NMO) | Not stated | Non caucasian |
| 85509 | Pakeha | White | Caucasian |
| 110695 | White: other White ethnic grp- Scotland ethnic cat 2011 cens | White | Caucasian |
| 46047 | Other Black or Black unspecified ethnic category 2001 census | Black | Non caucasian |
| 110464 | Asian/Asian British:Pakistani- Eng+Wales eth cat 2011 census | Asian or Asian British | Non caucasian |
| 46964 | Israeli - ethnic category 2001 census | Other ethnic groups | Non caucasian |
| 110536 | Mixed: other Mixed/multiple ethnic backgrd - NI 2011 census | Mixed | Non caucasian |
| 111064 | Asian: Chinese - Scotland ethnic category 2011 census | Mixed | Non caucasian |
| 54593 | Caribbean I./W.I./Guyana (NMO) | Black | Non caucasian |
| 110555 | Other ethnic group: Arab - Eng+Wales ethnic cat 2011 census | Other ethnic groups | Non caucasian |
| 98111 | White British - ethnic category 2001 census | White | Caucasian |
| 57286 | New Zealand European | Other ethnic groups | Non caucasian |
| 12681 | Welsh - ethnic category 2001 census | White | Caucasian |
| 110538 | Asian/Asian British: Pakistani - NI ethnic cat 2011 census | Asian or Asian British | Non caucasian |
| 26341 | Kosovan - ethnic category 2001 census | White | Caucasian |
| 12446 | White British | White | Caucasian |
| 64609 | Fijian | Other ethnic groups | Non caucasian |
| 12757 | Other ethnic group | Other ethnic groups | Non caucasian |
| 25451 | Moroccan - ethnic category 2001 census | Other ethnic groups | Non caucasian |
| 32413 | Turkish Cypriot - ethnic category 2001 census | White | Caucasian |
| 12402 | Oth White European/European unsp/Mixed European 2001 census | White | Caucasian |
| 12434 | Other - ethnic category 2001 census | Other ethnic groups | Non caucasian |
| 12696 | Other ethnic, mixed origin | Mixed | Non caucasian |
| 45964 | Kurdish - ethnic category 2001 census | Other ethnic groups | Non caucasian |
| 42294 | Northern Irish - ethnic category 2001 census | Not stated | Non caucasian |
| 12513 | Other Asian background - ethnic category 2001 census | Asian or Asian British | Non caucasian |
| 12473 | Japanese - ethnic category 2001 census | Other ethnic groups | Non caucasian |
| 90712 | O/E - Australoid | Asian or Asian British | Non caucasian |
| 45947 | Greek/Greek Cypriot (NMO) | Other ethnic groups | Non caucasian |
| 12633 | Other European (NMO) | Other ethnic groups | Non caucasian |
| 110465 | White: Polish - Scotland ethnic category 2011 census | White | Caucasian |
| 26379 | Other Asian (NMO) | Asian or Asian British | Non caucasian |
| 110420 | White:Eng/Welsh/Scot/NI/Brit - England and Wales 2011 census | White | Caucasian |
| 99316 | Indo-Caribbean (NMO) | Mixed | Non caucasian |
| 12719 | Vietnamese - ethnic category 2001 census | Other ethnic groups | Non caucasian |
| 47949 | Greek Cypriot (NMO) | Other ethnic groups | Non caucasian |
| 55223 | Irish Traveller - ethnic category 2001 census | White | Caucasian |
| 110445 | Mixed: White+Black Caribbean - Eng+Wales eth cat 2011 census | Mixed | Non caucasian |
| 47028 | North African - ethnic category 2001 census | Other ethnic groups | Non caucasian |
| 12421 | Other White background - ethnic category 2001 census | White | Caucasian |
| 40096 | Mixed Black - ethnic category 2001 census | Black | Non caucasian |
| 110437 | Black/African/Carib/Black Brit: African- Eng+Wales 2011 cens | Black | Non caucasian |

# Chronic conditions

## Asthma

The code list for Medcodes is based on the code list in Forbes, H. J. *Understanding risk factors for herpes zoster and postherpetic neuralgia in UK primary care: investigations to inform vaccine policy*. Diss. London School of Hygiene & Tropical Medicine, 2016.

### Asthma Medcodes

| **Medcode** | **Description** |
| --- | --- |
| 78 | Asthma |
| 81 | Asthma monitoring |
| 185 | Acute exacerbation of asthma |
| 232 | Asthma attack |
| 233 | Severe asthma attack |
| 719 | H/O: asthma |
| 1208 | Childhood asthma |
| 1555 | Bronchial asthma |
| 2290 | Allergic asthma |
| 3018 | Mild asthma |
| 3366 | Severe asthma |
| 3458 | Occasional asthma |
| 3665 | Late onset asthma |
| 4442 | Asthma unspecified |
| 4606 | Exercise induced asthma |
| 4836 | Nocturnal cough / wheeze |
| 4892 | Status asthmaticus NOS |
| 5267 | Intrinsic asthma |
| 5627 | Hay fever with asthma |
| 5867 | Exercise induced asthma |
| 6707 | Extrinsic asthma with asthma attack |
| 7058 | Emergency admission, asthma |
| 7146 | Extrinsic (atopic) asthma |
| 7191 | Asthma limiting activities |
| 7229 | Asthma prophylactic medication used |
| 7378 | Asthma management plan given |
| 7416 | Asthma disturbing sleep |
| 7731 | Pollen asthma |
| 8335 | Asthma attack NOS |
| 8355 | Asthma monitored |
| 9018 | Number of asthma exacerbations in past year |
| 9552 | Change in asthma management plan |
| 9663 | Step up change in asthma management plan |
| 10043 | Asthma annual review |
| 10274 | Asthma medication review |
| 10487 | Asthma - currently active |
| 11022 | Asthma trigger |
| 11370 | Asthma confirmed |
| 12987 | Late-onset asthma |
| 13064 | Asthma severity |
| 13065 | Moderate asthma |
| 13173 | Asthma not disturbing sleep |
| 13174 | Asthma not limiting activities |
| 13175 | Asthma disturbs sleep frequently |
| 13176 | Asthma follow-up |
| 14777 | Extrinsic asthma without status asthmaticus |
| 15248 | Hay fever with asthma |
| 16070 | Asthma NOS |
| 16667 | Asthma control step 2 |
| 16785 | Asthma control step 1 |
| 18141 | Asthma monitoring due |
| 18223 | Step down change in asthma management plan |
| 18224 | Asthma control step 3 |
| 18323 | Intrinsic asthma with asthma attack |
| 19167 | Asthma monitoring by nurse |
| 19519 | Asthma treatment compliance unsatisfactory |
| 19520 | Asthma treatment compliance satisfactory |
| 19539 | Asthma monitoring check done |
| 20860 | Asthma control step 5 |
| 20886 | Asthma control step 4 |
| 21232 | Allergic asthma NEC |
| 22752 | Occupational asthma |
| 24479 | Emergency asthma admission since last appointment |
| 24506 | Further asthma - drug prevent. |
| 24884 | Asthma causes daytime symptoms 1 to 2 times per week |
| 25181 | Asthma restricts exercise |
| 25791 | Asthma clinical management plan |
| 25796 | Mixed asthma |
| 26501 | Asthma never causes daytime symptoms |
| 26503 | Asthma causes daytime symptoms most days |
| 26504 | Asthma never restricts exercise |
| 26506 | Asthma severely restricts exercise |
| 26861 | Asthma sometimes restricts exercise |
| 27926 | Extrinsic asthma with status asthmaticus |
| 29325 | Intrinsic asthma without status asthmaticus |
| 29645 | Asthma control step 0 |
| 30458 | Asthma monitoring by doctor |
| 30815 | Asthma causing night waking |
| 31167 | Asthma night-time symptoms |
| 31225 | Asthma causes daytime symptoms 1 to 2 times per month |
| 38143 | Asthma never disturbs sleep |
| 38144 | Asthma limits walking up hills or stairs |
| 38145 | Asthma limits walking on the flat |
| 38146 | Asthma disturbs sleep weekly |
| 39478 | Wood asthma |
| 39570 | Asthma causes night symptoms 1 to 2 times per month |
| 40823 | Brittle asthma |
| 40864 | [X] Adverse reaction to theophylline - asthma |
| 41017 | Aspirin induced asthma |
| 41020 | Absent from work or school due to asthma |
| 42824 | Asthma daytime symptoms |
| 45073 | Intrinsic asthma NOS |
| 45782 | Extrinsic asthma NOS |
| 46529 | Attends asthma monitoring |
| 47337 | Asthma accident and emergency attendance since last visit |
| 47684 | Detergent asthma |
| 48591 | Adverse reaction to theophylline (asthma) |
| 58196 | Intrinsic asthma with status asthmaticus |
| 73522 | Work aggravated asthma |
| 98185 | Asthma control test |
| 99793 | Patient has a written asthma personal action plan |
| 100397 | Asthma control questionnaire |
| 100509 | Under care of asthma specialist nurse |
| 100740 | Health education - structured asthma discussion |
| 102170 | Asthma review using Roy Colleg of Physicians three questions |
| 102209 | Mini asthma quality of life questionnaire |
| 102301 | Asthma trigger - seasonal |
| 102341 | Asthma trigger - pollen |
| 102395 | Asthma causes symptoms most nights |
| 102395 | Asthma causes symptoms most nights |
| 102395 | Asthma causes symptoms most nights |
| 102395 | Asthma causes symptoms most nights |
| 102400 | Asthma causes night time symptoms 1 to 2 times per week |
| 102449 | Asthma trigger - respiratory infection |
| 102713 | Asthma limits activities 1 to 2 times per month |
| 102871 | Asthma trigger - exercise |
| 102888 | Asthma limits activities 1 to 2 times per week |
| 102952 | Asthma trigger - warm air |
| 103318 | Health education - structured patient focused asthma discuss |
| 103321 | Asthma trigger - animals |

### Asthma ICD-10 codes

| **ICD-10** | **Description** |
| --- | --- |
| J45.0 | Predominantly allergic asthma |
| J45.1 | Nonallergic asthma |
| J45.8 | Mixed asthma |
| J45.9 | Asthma, unspecified |

## Diabetes type 2

The algorithm to classify subjects into type 1 and type 2, adapted from Taylor et al. [14] is given below. Only type 2 diabetes is included as a risk factor in the analysis.

| **Stage** | **If** | **Then** | **Else** |
| --- | --- | --- | --- |
| 0 | Any diagnosis, treatment or abnormal test with missing event date OR (exactly 1 treatment AND no Read or abnormal test) | No Type 1/2 | Go to stage 1 |
| 1 | Any diagnosis, treatment or abnormal test | Go to stage 2 | No Type1/2 |
| 2 | Type 1 Read and no other Read except Unspecified | Type 1 | Go to stage 3 |
| 3 | Type 2 Read and no Type 1 Read | Type 2 | Go to stage 4 |
| 4 | Both Type 1 and Type 2 Read OR no Read except Unspecified | Go to stage 5 | No Type 1/2 |
| 5 | Never on insulin (insulin: TreatmentProgression=5) | Go to stage 7 | Go to stage 6 |
| 6 | Insulin concurrent with other antidiabetic except metformin: prescriptions for other antidiabetic separated by no more than 180 days between diabetes onset and end of follow-up in database (other antidiabetic except metformin: TreatmentProgression=2) | Go to stage 7 | No Type 1/2 |
| 7 | >= 25 years old at DM onset date | Type 2 | No Type 1/2 |

### Diabetes type 2 Medcodes

| **Medcode** | **Description** | **ClassType** |
| --- | --- | --- |
| 506 | Non-insulin dependent diabetes mellitus | Type 2 |
| 711 | Diabetes mellitus | NOS |
| 758 | Type 2 diabetes mellitus | Type 2 |
| 1038 | Insulin dependent diabetes mellitus | Type 1 |
| 1323 | Diabetic retinopathy | NOS |
| 1407 | Insulin treated Type 2 diabetes mellitus | Type 2 |
| 1549 | Type 1 diabetes mellitus | Type 1 |
| 1647 | Insulin dependent diabetes mellitus | Type 1 |
| 1682 | Diabetes mellitus with ketoacidosis | NOS |
| 2340 | Diabetic amyotrophy | NOS |
| 2342 | Diabetic neuropathy | NOS |
| 2471 | Nephrotic syndrome in diabetes mellitus | NOS |
| 2475 | Diabetic nephropathy | NOS |
| 2664 | Gestational diabetes mellitus | Gestational DM |
| 2986 | Preproliferative diabetic retinopathy | NOS |
| 3286 | Proliferative diabetic retinopathy | NOS |
| 3837 | Diabetic maculopathy | NOS |
| 4513 | Non-insulin dependent diabetes mellitus | Type 2 |
| 5002 | Diabetic polyneuropathy | NOS |
| 5884 | NIDDM - Non-insulin dependent diabetes mellitus | Type 2 |
| 6509 | Insulin dependent diabetes mellitus with retinopathy | Type 1 |
| 7069 | Background diabetic retinopathy | NOS |
| 7795 | Diabetes mellitus with neuropathy | NOS |
| 8403 | Non-insulin dependent diabetes mellitus - poor control | Type 2 |
| 8446 | Gestational diabetes mellitus | Gestational DM |
| 9835 | O/E - diabetic maculopathy present both eyes | NOS |
| 10099 | Advanced diabetic maculopathy | NOS |
| 10278 | Diabetes mellitus arising in pregnancy | Gestational DM |
| 10418 | Type 1 diabetes mellitus with nephropathy | Type 1 |
| 10659 | Diabetic cataract | NOS |
| 10692 | Type 1 diabetes mellitus with ketoacidosis | Type 1 |
| 10755 | Non proliferative diabetic retinopathy | NOS |
| 11129 | O/E - left eye background diabetic retinopathy | NOS |
| 11359 | Diabetes mellitus during pregnancy/childbirth/puerperium | Gestational DM |
| 11433 | O/E - right eye background diabetic retinopathy | NOS |
| 11551 | Diabetes mellitus induced by steroids | NOS |
| 11551 | Diabetes mellitus induced by steroids | Other transient |
| 11626 | Diabetic retinopathy NOS | NOS |
| 12640 | Type 2 diabetes mellitus with nephropathy | Type 2 |
| 13099 | O/E - right eye preproliferative diabetic retinopathy | NOS |
| 13101 | O/E - left eye proliferative diabetic retinopathy | NOS |
| 13103 | O/E - left eye preproliferative diabetic retinopathy | NOS |
| 13279 | Other specified diabetes mellitus with renal complications | NOS |
| 14803 | Diabetes mellitus, adult onset, no mention of complication | NOS |
| 14889 | Maturity onset diabetes | NOS |
| 16230 | Diabetes mellitus with neurological manifestation | NOS |
| 16491 | Diabetes mellitus with polyneuropathy | NOS |
| 16502 | Diabetes mellitus with renal manifestation | NOS |
| 17067 | Autonomic neuropathy due to diabetes | NOS |
| 17247 | Diabetic mononeuritis NOS | NOS |
| 17262 | Non-insulin-dependent diabetes mellitus with retinopathy | Type 2 |
| 17313 | Diabetic iritis | NOS |
| 17545 | Type I diabetes mellitus with diabetic cataract | Type 1 |
| 17858 | Type 1 diabetes mellitus | Type 1 |
| 17859 | Type 2 diabetes mellitus | Type 2 |
| 18209 | Type 2 diabetes mellitus with renal complications | Type 2 |
| 18219 | Type II diabetes mellitus | Type 2 |
| 18230 | Type 1 diabetes mellitus with neuropathic arthropathy | Type 1 |
| 18278 | Insulin treated Type 2 diabetes mellitus | Type 2 |
| 18387 | Type 1 diabetes mellitus with retinopathy | Type 1 |
| 18390 | Type 2 diabetes mellitus with persistent microalbuminuria | Type 2 |
| 18425 | Type 2 diabetes mellitus with polyneuropathy | Type 2 |
| 18496 | Type 2 diabetes mellitus with retinopathy | Type 2 |
| 18505 | IDDM-Insulin dependent diabetes mellitus | Type 1 |
| 18777 | Type 2 diabetes mellitus with renal complications | Type 2 |
| 21482 | Diabetes mellitus with hyperosmolar coma | NOS |
| 21983 | Type 1 diabetes mellitus with renal complications | Type 1 |
| 22487 | Secondary diabetes mellitus | NOS |
| 22573 | Diabetes mellitus NOS with neurological manifestation | NOS |
| 22884 | Type II diabetes mellitus | Type 2 |
| 24423 | Type I diabetes mellitus | Type 1 |
| 24490 | Diabetes mellitus, juvenile type, no mention of complication | NOS |
| 24571 | Asymptomatic diabetic neuropathy | NOS |
| 24694 | Insulin dependent diabetes mellitus with mononeuropathy | Type 1 |
| 24836 | Type 2 diabetes mellitus with nephropathy | Type 2 |
| 25591 | Type 2 diabetes mellitus with exudative maculopathy | Type 2 |
| 25627 | Type 2 diabetes mellitus - poor control | Type 2 |
| 26054 | Type 2 diabetes mellitus with persistent proteinuria | Type 2 |
| 26108 | Steroid induced diabetes mellitus without complication | NOS |
| 29979 | Non-insulin-dependent diabetes mellitus without complication | Type 2 |
| 30294 | Type 1 diabetes mellitus with persistent microalbuminuria | Type 1 |
| 30323 | Type 1 diabetes mellitus with persistent proteinuria | Type 1 |
| 30477 | High risk proliferative diabetic retinopathy | NOS |
| 31310 | Insulin dependent diabetes maturity onset | Type 1 |
| 31790 | Polyneuropathy in diabetes | NOS |
| 32193 | Steroid induced diabetes | Other transient |
| 33254 | Diabetes mellitus with ophthalmic manifestation | NOS |
| 33343 | Diabetes mellitus with other specified manifestation | NOS |
| 33807 | Diabetes mellitus, adult with gangrene | NOS |
| 33969 | Malnutrition-related diabetes mellitus with ketoacidosis | NOS |
| 34268 | Type 2 diabetes mellitus with neurological complications | Type 2 |
| 34283 | Diabetes mellitus NOS with ophthalmic manifestation | NOS |
| 34450 | Hyperosmolar non-ketotic state in type 2 diabetes mellitus | Type 2 |
| 34639 | Diabetes mellitus during pregnancy - baby delivered | Gestational DM |
| 34912 | Non-insulin dependent diabetes mellitus with ulcer | Type 2 |
| 35107 | Diabetes mellitus with nephropathy NOS | NOS |
| 35385 | Type 2 diabetes mellitus with neuropathic arthropathy | Type 2 |
| 35399 | Diabetes mellitus with peripheral circulatory disorder | NOS |
| 35785 | Chronic painful diabetic neuropathy | NOS |
| 36633 | Hyperosmolar non-ketotic state in type 2 diabetes mellitus | Type 2 |
| 36695 | Diabetes mellitus autosomal dominant type 2 | Type 2 |
| 37315 | Diabetic mononeuropathy | NOS |
| 37806 | Type 2 diabetes mellitus with peripheral angiopathy | Type 2 |
| 38161 | Type I diabetes mellitus with retinopathy | Type 1 |
| 38986 | Diabetes mellitus with no mention of complication | NOS |
| 39317 | Diabetes mellitus, adult onset, + neurological manifestation | Type 2 |
| 39420 | Myasthenic syndrome due to diabetic amyotrophy | NOS |
| 40682 | Type 1 diabetes mellitus maturity onset | Type 1 |
| 40837 | Type 1 diabetes mellitus with ketoacidotic coma | Type 1 |
| 40962 | Non-insulin dependent d m with neuropathic arthropathy | Type 2 |
| 41049 | Type 1 diabetes mellitus with retinopathy | Type 1 |
| 41389 | Diabetes mellitus, adult onset, + ophthalmic manifestation | NOS |
| 41716 | Insulin dependent diabetes mellitus with polyneuropathy | Type 1 |
| 42505 | Diabetes mellitus NOS with ketoacidosis | NOS |
| 42762 | Type 2 diabetes mellitus with retinopathy | Type 2 |
| 42831 | Type 1 diabetes mellitus with neurological complications | Type 1 |
| 43139 | Diabetes mellitus, adult onset, with hyperosmolar coma | NOS |
| 43227 | Type II diabetes mellitus with multiple complications | Type 2 |
| 43453 | Diabetes mellitus autosomal dominant | NOS |
| 44260 | Insulin dependent diabetes mellitus with diabetic cataract | Type 1 |
| 44440 | Insulin dependent diabetes mellitus with hypoglycaemic coma | Type 1 |
| 44779 | Type 2 diabetes mellitus with diabetic cataract | Type 2 |
| 44982 | Type 2 diabetes mellitus with diabetic cataract | Type 2 |
| 45467 | Non-insulin dependent diabetes mellitus with polyneuropathy | Type 2 |
| 45491 | Diabetes mellitus with unspecified complication | NOS |
| 45499 | Kimmelstiel - Wilson disease | NOS |
| 45919 | Type 2 diabetes mellitus with neurological complications | Type 2 |
| 46301 | Type 1 diabetes mellitus with polyneuropathy | Type 1 |
| 46624 | Maturity onset diabetes in youth | NOS |
| 46963 | Insulin-dependent diabetes mellitus with renal complications | Type 1 |
| 47315 | Type II diabetes mellitus - poor control | Type 2 |
| 47321 | Type 2 diabetes mellitus with ophthalmic complications | Type 2 |
| 47328 | O/E - right eye stable treated prolif diabetic retinopathy | NOS |
| 47377 | Other specified diabetes mellitus with ophthalmic complicatn | NOS |
| 47409 | Type II diabetes mellitus with polyneuropathy | Type 2 |
| 47582 | Type 1 diabetes mellitus with renal complications | Type 1 |
| 47649 | Type 1 diabetes mellitus with ophthalmic complications | Type 1 |
| 47816 | Type II diabetes mellitus with neuropathic arthropathy | Type 2 |
| 47954 | Type 2 diabetes mellitus without complication | Type 2 |
| 48078 | Acute painful diabetic neuropathy | NOS |
| 48192 | Type II diabetes mellitus with diabetic cataract | Type 2 |
| 49074 | Type 2 diabetes mellitus with ulcer | Type 2 |
| 49146 | Type I diabetes mellitus with neurological complications | Type 1 |
| 49276 | Insulin-dependent diabetes mellitus with ophthalmic comps | Type 1 |
| 49554 | Type 1 diabetes mellitus with diabetic cataract | Type 1 |
| 49559 | Diabetes mellitus during pregnancy - baby not yet delivered | Gestational DM |
| 49655 | Type II diabetes mellitus with retinopathy | Type 2 |
| 50225 | Type II diabetes mellitus with renal complications | Type 2 |
| 50429 | Non-insulin-dependent diabetes mellitus with ophthalm comps | Type 2 |
| 50527 | Type II diabetes mellitus with polyneuropathy | Type 2 |
| 50813 | Type II diabetes mellitus with mononeuropathy | Type 2 |
| 50972 | Diabetes mellitus NOS with no mention of complication | NOS |
| 51261 | Insulin dependent diabetes mellitus | Type 1 |
| 51697 | Secondary pancreatic diabetes mellitus | NOS |
| 52041 | O/E - left eye stable treated prolif diabetic retinopathy | NOS |
| 52236 | Malnutrition-related diabetes mellitus | NOS |
| 52283 | Insulin-dependent diabetes mellitus with neurological comps | Type 1 |
| 52303 | Non-insulin-dependent diabetes mellitus with renal comps | Type 2 |
| 53392 | Type II diabetes mellitus without complication | Type 2 |
| 54212 | Non-insulin-dependent d m with peripheral angiopath | Type 2 |
| 55239 | Type 1 diabetes mellitus with gastroparesis | Type 1 |
| 55842 | Non-insulin-dependent diabetes mellitus with neuro comps | Type 2 |
| 56803 | NIDDM with peripheral circulatory disorder | NOS |
| 57278 | Type II diabetes mellitus with renal complications | Type 2 |
| 57621 | Insulin dependent diabetes mellitus with nephropathy | Type 1 |
| 58604 | Type II diabetes mellitus with retinopathy | Type 2 |
| 59365 | Non-insulin dependent diabetes mellitus with nephropathy | Type 2 |
| 59725 | Type II diabetes mellitus with ophthalmic complications | Type 2 |
| 59903 | Diabetic amyotrophy | NOS |
| 59991 | Maturity onset diabetes in youth type 2 | Type 2 |
| 60796 | Type II diabetes mellitus with persistent proteinuria | Type 2 |
| 61122 | Diabetes mellitus induced by non-steroid drugs | Other transient |
| 61344 | Type I diabetes mellitus with renal complications | Type 1 |
| 61523 | Other specified diabetes mellitus with neurological comps | NOS |
| 61829 | Type 1 diabetes mellitus with neurological complications | Type 1 |
| 62674 | Type 2 diabetes mellitus with mononeuropathy | Type 2 |
| 63357 | Diabetes mellitus, adult, + peripheral circulatory disorder | NOS |
| 63555 | Polyneuropathy in disease NOS | NOS |
| 63690 | Type 2 diabetes mellitus with gastroparesis | Type 2 |
| 64384 | Diabetes mellitus in pregnancy/childbirth/puerperium NOS | Gestational DM |
| 64571 | Type II diabetes mellitus with nephropathy | Type 2 |
| 65025 | Diabetes mellitus NOS with peripheral circulatory disorder | NOS |
| 65267 | Type 2 diabetes mellitus with multiple complications | Type 2 |
| 65463 | High risk non proliferative diabetic retinopathy | NOS |
| 65616 | Insulin dependent diabetes mellitus with arthropathy | Type 1 |
| 66872 | Type I diabetes mellitus with nephropathy | Type 1 |
| 66965 | Type 2 diabetes mellitus with neuropathic arthropathy | Type 2 |
| 67853 | Diabetes mellitus, juvenile, + neurological manifestation | NOS |
| 67905 | Type II diabetes mellitus with neurological complications | Type 2 |
| 68105 | Type 1 diabetes mellitus with mononeuropathy | Type 1 |
| 69278 | Non-insulin depend diabetes mellitus with diabetic cataract | Type 2 |
| 69748 | Diabetes mellitus, juvenile type, ophthalmic manifestation | NOS |
| 70316 | Type 2 diabetes mellitus with ophthalmic complications | Type 2 |
| 72320 | Non-insulin dependent diabetes mellitus with mononeuropathy | Type 2 |
| 74638 | NEUROPATHY DIABETIC | NOS |
| 74790 | DIABETIC NEPHROPATHY | NOS |
| 81669 | DIABETIC GLOMERULOSCLEROSIS | NOS |
| 87626 | CHARCOT'S DIABETIC ARTHROPATHY | NOS |
| 93922 | Diabetes mellitus, juvenile type, with renal manifestation | NOS |
| 94383 | Secondary diabetes mellitus without complication | NOS |
| 94777 | [V]Personal history of gestational diabetes mellitus | Gestational DM |
| 95343 | Type I diabetes mellitus with retinopathy | Type 1 |
| 95351 | Type II diabetes mellitus with mononeuropathy | Type 2 |
| 95636 | Latent autoimmune diabetes mellitus in adult | NOS |
| 96823 | Diabetes mellitus in pueperium - baby previously delivered | Gestational DM |
| 98392 | Maturity onset diabetes in youth type 1 | Type 1 |
| 99231 | Type I diabetes mellitus with mononeuropathy | Type 1 |
| 102201 | Type II diabetes mellitus with nephropathy | Type 2 |
| 104588 | Gestational diabetes mellitus annual review | Gestational DM |
| 108013 | Dietary advice for gestational diabetes | Gestational DM |

### Diabetes type 2 Product codes

| **Prodcode** | **Description** | **TreatmentProgression** |
| --- | --- | --- |
| 23 | Metformin 500mg tablets | 1 |
| 32 | Gliclazide 80mg tablets | 2 |
| 93 | Metformin 850mg tablets | 1 |
| 322 | Humalog 100units/ml solution for injection 1.5ml cartridges (Eli Lilly and Company Ltd) | 5 |
| 469 | Rosiglitazone 4mg tablets | 2 |
| 479 | Acarbose 50mg tablets | 2 |
| 547 | Glipizide 2.5mg tablets | 2 |
| 548 | Pioglitazone 15mg tablets | 2 |
| 735 | Metformin 100mg/ml Oral solution | 1 |
| 804 | Glucagon 1mg powder and solvent for solution for injection vials | 0 |
| 1194 | MediSense G2 testing strips (Abbott Laboratories Ltd) | 0 |
| 1253 | Chlorpropamide 100mg tablets | 2 |
| 1254 | Glibenclamide 5mg tablets | 2 |
| 1274 | Glucotrend colorimetric strips Colorimetric strips (Roche Diagnostics Ltd) | 0 |
| 1587 | Monotard 100units/ml suspension for injection 10ml vials (Novo Nordisk Ltd) | 5 |
| 1588 | Actrapid 100iu/ml Injection (Novo Nordisk Ltd) | 5 |
| 1592 | Actrapid penfill 100 100iu/ml Penfill (Novo Nordisk Ltd) | 5 |
| 1593 | Insulatard penfill 100 100iu/ml Penfill (Novo Nordisk Ltd) | 5 |
| 1594 | Actrapid NovoLet 100units/ml solution for injection (Novo Nordisk Ltd) | 5 |
| 1595 | Insulatard NovoLet 100units/ml suspension for injection (Novo Nordisk Ltd) | 5 |
| 1649 | Human actraphane 100iu/ml Injection (Novo Nordisk Ltd) | 5 |
| 1805 | Mixtard 30/70 100unit/ml Injection (Novo Nordisk Ltd) | 5 |
| 1806 | Penmix 30/70 100iu/ml Penfill (Novo Nordisk Ltd) | 5 |
| 1840 | Humulin s 100unit/ml Injection (Eli Lilly and Company Ltd) | 5 |
| 1842 | Pork velosulin 100unit/ml Injection (Novo Nordisk Ltd) | 5 |
| 1843 | Pork Insulatard 100units/ml suspension for injection 10ml vials (Novo Nordisk Ltd) | 5 |
| 1844 | Ultratard 100units/ml suspension for injection 10ml vials (Novo Nordisk Ltd) | 5 |
| 1847 | Chlorpropamide 250mg tablets | 2 |
| 1886 | Insulatard 100iu/ml GE injection (Novo Nordisk Ltd) | 5 |
| 1964 | Diamicron 80mg tablets (Servier Laboratories Ltd) | 2 |
| 1965 | Tolbutamide 500mg tablets | 2 |
| 2219 | Glibenclamide 2.5mg tablets | 2 |
| 2220 | Penmix 20/80 Pen (Novo Nordisk Ltd) | 5 |
| 2221 | Mixtard 30 NovoLet 100units/ml suspension for injection (Novo Nordisk Ltd) | 5 |
| 2374 | OneTouch testing strips (LifeScan) | 0 |
| 2454 | Mixtard 30 penfill 100 100iu/ml Penfill (Novo Nordisk Ltd) | 5 |
| 2455 | Mixtard 20 NovoLet 100units/ml suspension for injection (Novo Nordisk Ltd) | 5 |
| 2456 | Mixtard 10 NovoLet 100units/ml suspension for injection (Novo Nordisk Ltd) | 5 |
| 2459 | Pork Mixtard 30 100units/ml suspension for injection 10ml vials (Novo Nordisk Ltd) | 5 |
| 2812 | Mixtard 40 NovoLet 100units/ml suspension for injection (Novo Nordisk Ltd) | 5 |
| 2929 | Mixtard 30 100iu/ml GE injection (Novo Nordisk Ltd) | 5 |
| 3396 | Penmix 10/90 Penfill (Novo Nordisk Ltd) | 5 |
| 3439 | Penmix 10/90 Pen (Novo Nordisk Ltd) | 5 |
| 3550 | Mixtard 40 penfill 100 100iu/ml Penfill (Novo Nordisk Ltd) | 5 |
| 3551 | Mixtard 20 penfill 100 100iu/ml Penfill (Novo Nordisk Ltd) | 5 |
| 3740 | Guar gum 90% granules | 2 |
| 4093 | Humulin M2 100units/ml suspension for injection 3ml cartridges (Eli Lilly and Company Ltd) | 5 |
| 4129 | Insulin soluble porcine 100units/ml solution for injection 1.5ml cartridges | 5 |
| 4156 | Hypostop Gel (Bio-Diagnostics Ltd) | 0 |
| 4163 | Rapitard MC 100unit/ml Injection (Novo Nordisk Ltd) | 5 |
| 4198 | Humulin m3 100unit/ml M3 injection (Eli Lilly and Company Ltd) | 5 |
| 4199 | Humulin m1 100unit/ml M1 injection (Eli Lilly and Company Ltd) | 5 |
| 4247 | Insulin isophane porcine 100units/ml suspension for injection 1.5ml cartridges | 5 |
| 4307 | Guarina Sachets (Norgine Pharmaceuticals Ltd) | 2 |
| 4362 | Glucagon novo 1mg Injection (Novo Nordisk Ltd) | 0 |
| 4706 | Velosulin 100units/ml solution for injection 10ml vials (Novo Nordisk Ltd) | 5 |
| 4715 | Humalog mix 25 25/75 100units/ml Injection (Eli Lilly and Company Ltd) | 5 |
| 4760 | Humulin i 100unit/ml Injection (Eli Lilly and Company Ltd) | 5 |
| 4784 | Lentard mc 100unit/ml Injection (Novo Nordisk Ltd) | 5 |
| 4790 | Mixtard 50 penfill 100 100iu/ml Penfill (Novo Nordisk Ltd) | 5 |
| 4813 | GlucaGen Hypokit 1mg powder and solvent for solution for injection (Novo Nordisk Ltd) | 0 |
| 4862 | Diabetamide 2.5mg tablets (Ashbourne Pharmaceuticals Ltd) | 2 |
| 5021 | NovoRapid Penfill 100units/ml solution for injection 3ml cartridges (Novo Nordisk Ltd) | 5 |
| 5174 | Acarbose 100mg tablets | 2 |
| 5214 | Insulin lispro 100units/ml solution for injection 1.5ml cartridges | 5 |
| 5227 | Rosiglitazone 8mg tablets | 2 |
| 5250 | Insulin biphasic lispro human prb 25:75; 100 units/ml Injection | 5 |
| 5255 | Mixtard 10 penfill 100 100iu/ml Penfill (Novo Nordisk Ltd) | 5 |
| 5276 | Glimepiride 1mg tablets | 2 |
| 5316 | Glimepiride 4mg tablets | 2 |
| 5353 | Glimepiride 2mg tablets | 2 |
| 5392 | GlucoTip Fine lancets 0.45mm/26gauge (A Menarini Diagnostics Ltd) | 0 |
| 5501 | Insuman basal 100iu/ml Injection (Aventis Pharma) | 5 |
| 5621 | Glucobay 50mg tablets (Bayer Plc) | 2 |
| 5627 | Gliclazide 30mg modified-release tablets | 2 |
| 5636 | Glipizide 5mg tablets | 2 |
| 5678 | Nateglinide 120mg tablets | 2 |
| 5691 | Multistix GP Reagent strips (Bayer Diagnostics Manufacturing Ltd) | 0 |
| 5845 | Mixtard 30 InnoLet 100units/ml suspension for injection 3ml pre-filled pen (Novo Nordisk Ltd) | 5 |
| 5891 | Insulatard FlexPen 100units/ml suspension for injection (Novo Nordisk Ltd) | 5 |
| 5892 | NovoRapid FlexPen 100units/ml solution for injection 3ml pre-filled pen (Novo Nordisk Ltd) | 5 |
| 5933 | Mixtard 50 NovoLet 100units/ml suspension for injection (Novo Nordisk Ltd) | 5 |
| 5953 | Insulin glargine 100iu/ml Injection | 5 |
| 5989 | Nateglinide 180mg tablets | 2 |
| 6005 | Glucose 40% oral gel | 0 |
| 6057 | Lantus 100iu/ml Injection (Aventis Pharma) | 5 |
| 6061 | Novomix 30 30/70 100units/ml Injection (Novo Nordisk Ltd) | 5 |
| 6209 | NovoRapid 100units/ml solution for injection 10ml vials (Novo Nordisk Ltd) | 5 |
| 6265 | Diazoxide 50mg tablets | 0 |
| 6337 | Glimepiride 3mg tablets | 2 |
| 6447 | Insulin aspart human pyr 100 iu/ml Injection | 5 |
| 6855 | Avandamet 2mg/500mg tablets (GlaxoSmithKline UK Ltd) | 2 |
| 6958 | Levemir FlexPen 100units/ml solution for injection 3ml pre-filled pen (Novo Nordisk Ltd) | 5 |
| 6965 | Levemir Penfill 100units/ml solution for injection 3ml cartridges (Novo Nordisk Ltd) | 5 |
| 7048 | Metformin 500mg modified-release tablets | 1 |
| 7166 | Glucophage 500mg tablets (Merck Serono Ltd) | 1 |
| 7179 | Ascensia Autodisc testing discs (Bayer Diagnostics Manufacturing Ltd) | 0 |
| 7220 | GlucoGel 40% gel original (BBI Healthcare Ltd) | 0 |
| 7228 | NovoMix 30 FlexPen 100units/ml suspension for injection 3ml pre-filled pen (Novo Nordisk Ltd) | 5 |
| 7231 | Mixtard 30 Penfill 100units/ml suspension for injection 3ml cartridges (Novo Nordisk Ltd) | 5 |
| 7237 | Lantus 100units/ml solution for injection 3ml pre-filled OptiSet pen (Sanofi) | 5 |
| 7266 | Lantus 100units/ml solution for injection 3ml cartridges (Sanofi) | 5 |
| 7267 | NovoMix 30 Penfill 100units/ml suspension for injection 3ml cartridges (Novo Nordisk Ltd) | 5 |
| 7284 | Amaryl 2mg tablets (Zentiva) | 2 |
| 7300 | Mixtard 30 100units/ml suspension for injection 10ml vials (Novo Nordisk Ltd) | 5 |
| 7318 | Humalog 100units/ml solution for injection 3ml cartridges (Eli Lilly and Company Ltd) | 5 |
| 7319 | Mixtard 20 Penfill 100units/ml suspension for injection 3ml cartridges (Novo Nordisk Ltd) | 5 |
| 7325 | Avandamet 4mg/1000mg tablets (GlaxoSmithKline UK Ltd) | 2 |
| 7332 | Amaryl 1mg tablets (Zentiva) | 2 |
| 7349 | Actrapid 100units/ml solution for injection 10ml vials (Novo Nordisk Ltd) | 5 |
| 7350 | Insulin isophane porcine 100units/ml suspension for injection 10ml vials | 5 |
| 7375 | Rosiglitazone 4mg / Metformin 1g tablets | 2 |
| 7393 | Insulin glargine 100units/ml solution for injection 3ml cartridges | 5 |
| 7400 | Insulin glargine 100units/ml solution for injection 3ml pre-filled disposable devices | 5 |
| 7402 | Lantus 100units/ml solution for injection 10ml vials (Sanofi) | 5 |
| 7409 | Amaryl 3mg tablets (Zentiva) | 2 |
| 7537 | Humulin Zn 100units/ml suspension for injection 10ml vials (Eli Lilly and Company Ltd) | 5 |
| 7610 | Glucophage 850mg tablets (Merck Serono Ltd) | 1 |
| 7695 | Guarem Sachets (Shire Pharmaceuticals Ltd) | 2 |
| 7744 | Daonil 5mg tablets (Sanofi) | 2 |
| 7771 | Human protaphane penfill 100 100unit/ml Penfill (Novo Nordisk Ltd) | 5 |
| 7772 | Human protaphane 100unit/ml Injection (Novo Nordisk Ltd) | 5 |
| 7793 | HumaJect M3 Pen 100units/ml suspension for injection (Eli Lilly and Company Ltd) | 5 |
| 7912 | Semi-Daonil 2.5mg tablets (Sanofi) | 2 |
| 8034 | Diabinese 100mg Tablet (Pfizer Ltd) | 2 |
| 8118 | Humaject i 100iu/ml Pen (Eli Lilly and Company Ltd) | 5 |
| 8168 | Diabinese 250mg Tablet (Pfizer Ltd) | 2 |
| 8203 | Penmix 50/50 100iu/ml Penfill (Novo Nordisk Ltd) | 5 |
| 8322 | Insulin zinc suspension mixed human pyr 100unit/ml Injection | 5 |
| 8390 | Gliquidone 30mg tablets | 2 |
| 8841 | Humulin M5 100units/ml suspension for injection 10ml vials (Eli Lilly and Company Ltd) | 5 |
| 8895 | Initard 50/50 100unit/ml Injection (Novo Nordisk Ltd) | 5 |
| 8976 | Euglucon 2.5mg tablets (Aventis Pharma) | 2 |
| 9105 | Glucobay 100mg tablets (Bayer Plc) | 2 |
| 9341 | Insulin biphasic isophane human prb 30:70; 100 units/ml Injection | 5 |
| 9376 | Insulin zinc suspension crystalline human pyr 100unit/ml long acting Injection | 5 |
| 9503 | Hypurin Bovine Protamine Zinc 100units/ml suspension for injection 10ml vials (Wockhardt UK Ltd) | 5 |
| 9521 | Pork Actrapid 100units/ml solution for injection 10ml vials (Novo Nordisk Ltd) | 5 |
| 9565 | HumaJect S Pen 100units/ml solution for injection (Eli Lilly and Company Ltd) | 5 |
| 9618 | Hypurin Porcine 30/70 Mix 100units/ml suspension for injection 1.5ml cartridges (C P Pharmaceuticals Ltd) | 5 |
| 9662 | Avandia 4mg tablets (GlaxoSmithKline UK Ltd) | 2 |
| 9699 | Pioglitazone 30mg tablets | 2 |
| 9707 | Repaglinide 1mg tablets | 2 |
| 9737 | Insulatard innolet 100iu/ml Injection (Novo Nordisk Ltd) | 5 |
| 9748 | Repaglinide 2mg tablets | 2 |
| 9865 | Repaglinide 500microgram tablets | 2 |
| 10001 | Humalog Mix50 Pen 100units/ml suspension for injection 3ml pre-filled pen (Eli Lilly and Company Ltd) | 5 |
| 10051 | Pioglitazone 45mg tablets | 2 |
| 10067 | Insulin biphasic aspart human pyr 30:70; 100 units/ml Injection | 5 |
| 10175 | Insulin isophane human 100units/ml suspension for injection 1.5ml cartridges | 5 |
| 10184 | Insulin detemir 100 iu/ml Solution for injection | 5 |
| 10207 | Insulin isophane human 100units/ml suspension for injection 3ml cartridges | 5 |
| 10208 | Insulatard InnoLet 100units/ml suspension for injection 3ml pre-filled pen (Novo Nordisk Ltd) | 5 |
| 10225 | Lantus 100units/ml solution for injection 3ml OptiClik cartridges (Sanofi) | 5 |
| 10229 | Humulin I Pen 100units/ml suspension for injection 3ml pre-filled pen (Eli Lilly and Company Ltd) | 5 |
| 10243 | Humalog Mix25 100units/ml suspension for injection 3ml cartridges (Eli Lilly and Company Ltd) | 5 |
| 10244 | Mixtard 40 Penfill 100units/ml suspension for injection 3ml cartridges (Novo Nordisk Ltd) | 5 |
| 10245 | Mixtard 10 Penfill 100units/ml suspension for injection 3ml cartridges (Novo Nordisk Ltd) | 5 |
| 10259 | Insulin glargine 100units/ml solution for injection 10ml vials | 5 |
| 10264 | Humalog Pen 100units/ml solution for injection 3ml pre-filled pen (Eli Lilly and Company Ltd) | 5 |
| 10277 | Humulin M3 100units/ml suspension for injection 3ml cartridges (Eli Lilly and Company Ltd) | 5 |
| 10427 | Tolazamide 250mg Tablet | 2 |
| 10484 | Penmix 20/80 Penfill (Novo Nordisk Ltd) | 5 |
| 10547 | Humulin Lente 100units/ml suspension for injection 10ml vials (Eli Lilly and Company Ltd) | 5 |
| 10572 | Insulin soluble bovine 100unit/ml Injection | 5 |
| 10887 | Penmix 40/60 100iu/ml Penfill (Novo Nordisk Ltd) | 5 |
| 10910 | Humaject m2 100iu/ml M2 pen (Eli Lilly and Company Ltd) | 5 |
| 10915 | Humaject m1 100iu/ml M1 pen (Eli Lilly and Company Ltd) | 5 |
| 11055 | Insulin biphasic isophane human pyr 20:80; 100 units/ml Injection | 5 |
| 11056 | Insulin biphasic isophane human pyr 30:70; 100 units/ml Injection | 5 |
| 11080 | Insulin isophane human prb 100iu/ml Injection | 5 |
| 11107 | Humulin m4 100unit/ml M4 injection (Eli Lilly and Company Ltd) | 5 |
| 11284 | Amaryl 4mg tablets (Zentiva) | 2 |
| 11316 | NovoNorm 500microgram tablets (Novo Nordisk Ltd) | 2 |
| 11321 | NovoNorm 1mg tablets (Novo Nordisk Ltd) | 2 |
| 11337 | NovoRapid Novolet 100units/ml solution for injection (Novo Nordisk Ltd) | 5 |
| 11366 | NovoNorm 2mg tablets (Novo Nordisk Ltd) | 2 |
| 11483 | Nateglinide 60mg tablets | 2 |
| 11601 | Rosiglitazone 2mg / Metformin 500mg tablets | 2 |
| 11604 | Rosiglitazone 1mg / Metformin 500mg tablets | 2 |
| 11609 | Metformin with rosiglitazone 500mg + 1mg Tablet | 2 |
| 11610 | Metformin with rosiglitazone 500mg + 2mg Tablet | 2 |
| 11695 | Diamicron 30mg MR tablets (Servier Laboratories Ltd) | 2 |
| 11717 | Rosiglitazone 2mg / Metformin 1g tablets | 2 |
| 11737 | Metformin with rosiglitazone 1000mg + 4mg Tablet | 2 |
| 11760 | Metformin with rosiglitazone 1000mg + 2mg Tablet | 2 |
| 11946 | Tolbutamide 50mg/ml Injection | 2 |
| 11990 | Metformin 500mg/5ml oral solution sugar free | 1 |
| 12035 | Insulin zinc mixed bovine 100units/ml suspension for injection 10ml vials | 5 |
| 12245 | Glutril 25mg Tablet (Roche Products Ltd) | 2 |
| 12259 | Glibornuride 25mg Tablet | 2 |
| 12297 | Hypurin bovine neutral 100unit/ml Injection (C P Pharmaceuticals Ltd) | 5 |
| 12299 | Semitard mc 100unit/ml Injection (Novo Nordisk Ltd) | 5 |
| 12455 | Rastinon 500mg Tablet (Hoechst Marion Roussel) | 2 |
| 12513 | Glibenese 5mg tablets (Pfizer Ltd) | 2 |
| 12638 | Insulin soluble human pyr 100unit/ml Injection | 5 |
| 12654 | Insulin soluble human prb 100unit/ml Injection | 5 |
| 12818 | Human Mixtard 50 100units/ml suspension for injection 10ml vials (Novo Nordisk Ltd) | 5 |
| 12897 | Guar gum 5g granules sachets sugar free | 0 |
| 13277 | Mixtard 50 Penfill 100units/ml suspension for injection 3ml cartridges (Novo Nordisk Ltd) | 5 |
| 13331 | Euglucon 5mg tablets (Sanofi) | 2 |
| 13416 | Insulin biphasic 100 units/ml Injection | 5 |
| 13516 | Hypurin bovine isophane 100unit/ml Injection (C P Pharmaceuticals Ltd) | 5 |
| 13622 | Hypurin porcine neutral 100unit/ml Injection (C P Pharmaceuticals Ltd) | 5 |
| 13628 | Romozin 400mg Tablet (Glaxo Wellcome UK Ltd) | 2 |
| 13729 | Insulin isophane human emp 100unit/ml Injection | 5 |
| 13819 | Hypurin Porcine Isophane 100units/ml suspension for injection 1.5ml cartridges (C P Pharmaceuticals Ltd) | 5 |
| 13837 | Insulin biphasic isophane human prb 10:90; 100 units/ml Injection | 5 |
| 14164 | Avandamet 2mg/1000mg tablets (GlaxoSmithKline UK Ltd) | 2 |
| 14270 | Humalog Mix25 Pen 100units/ml suspension for injection 3ml pre-filled pen (Eli Lilly and Company Ltd) | 5 |
| 14290 | Insulatard Penfill 100units/ml suspension for injection 3ml cartridges (Novo Nordisk Ltd) | 5 |
| 14299 | Insulin glulisine 100units/ml solution for injection 3ml cartridges | 5 |
| 14301 | Insulin detemir 100units/ml solution for injection 3ml cartridges | 5 |
| 14313 | Insulin lispro 100units/ml solution for injection 3ml cartridges | 5 |
| 14330 | Insulin detemir 100units/ml solution for injection 3ml pre-filled disposable devices | 5 |
| 14339 | Hypurin Bovine Neutral 100units/ml solution for injection 10ml vials (Wockhardt UK Ltd) | 5 |
| 14340 | Hypurin Bovine Isophane 100units/ml suspension for injection 10ml vials (Wockhardt UK Ltd) | 5 |
| 14345 | Apidra 100units/ml solution for injection 3ml cartridges (Sanofi) | 5 |
| 14357 | Humulin I 100units/ml suspension for injection 3ml cartridges (Eli Lilly and Company Ltd) | 5 |
| 14362 | Insulin lispro 100units/ml solution for injection 3ml pre-filled disposable devices | 5 |
| 14505 | Insulin protamine zinc bovine 100units/ml suspension for injection 10ml vials | 5 |
| 14619 | Insulin isophane biphasic porcine 30/70 100units/ml suspension for injection 1.5ml cartridges | 5 |
| 14644 | Insulin biphasic isophane human prb 20:80; 100 units/ml Injection | 5 |
| 14649 | Insulin biphasic isophane human pyr 10:90; 100 units/ml Injection | 5 |
| 14918 | Humulin I 100units/ml suspension for injection 10ml vials (Eli Lilly and Company Ltd) | 5 |
| 14925 | Insulin isophane human vial 100unit/ml Sterile suspension injection | 5 |
| 14928 | Insulatard 100units/ml suspension for injection 10ml vials (Novo Nordisk Ltd) | 5 |
| 14930 | Hypurin Porcine Neutral 100units/ml solution for injection 3ml cartridges (Wockhardt UK Ltd) | 5 |
| 14933 | Hypurin Porcine Isophane 100units/ml suspension for injection 3ml cartridges (Wockhardt UK Ltd) | 5 |
| 14938 | Insulin soluble bovine cartridge 100unit/ml Solution for injection | 5 |
| 14944 | Humulin S 100units/ml solution for injection 3ml cartridges (Eli Lilly and Company Ltd) | 5 |
| 15199 | Insuman comb 25 100iu/ml Injection (Aventis Pharma) | 5 |
| 15232 | Avandia 8mg tablets (GlaxoSmithKline UK Ltd) | 2 |
| 15374 | Gliclazide 40mg/5ml oral suspension | 2 |
| 15484 | Insulin isophane bovine 100units/ml suspension for injection 1.5ml cartridges | 5 |
| 15710 | Insulin soluble human emp 100unit/ml Injection | 5 |
| 15955 | Starlix 120mg tablets (Novartis Pharmaceuticals UK Ltd) | 2 |
| 15961 | Insulin isophane human crb 100iu/ml Injection | 5 |
| 16044 | Glucophage SR 500mg tablets (Merck Serono Ltd) | 1 |
| 16129 | Insulin soluble human 100units/ml solution for injection 3ml cartridges | 5 |
| 16142 | Insulin aspart 100units/ml solution for injection 3ml cartridges | 5 |
| 16152 | Insulin isophane biphasic human 30/70 100units/ml suspension for injection 3ml cartridges | 5 |
| 16160 | Humulin M3 Pen 100units/ml suspension for injection 3ml pre-filled pen (Eli Lilly and Company Ltd) | 5 |
| 16602 | Calabren 2.5mg Tablet (Berk Pharmaceuticals Ltd) | 2 |
| 16682 | Tempulin 100unit/ml Injection (Knoll Ltd) | 5 |
| 16700 | Insulin zinc mixed bovine vial 100unit/ml Sterile suspension injection | 5 |
| 17336 | Novopen 100unit/ml Injection device (Novo Nordisk Ltd) | 5 |
| 17343 | Gliclazide 80mg tablets (A A H Pharmaceuticals Ltd) | 2 |
| 17580 | Avandamet 1mg/500mg tablets (GlaxoSmithKline UK Ltd) | 2 |
| 17698 | Minodiab 5mg tablets (Pfizer Ltd) | 2 |
| 17706 | Minodiab 2.5mg tablets (Pfizer Ltd) | 2 |
| 17712 | Hypurin Bovine Lente 100units/ml suspension for injection 10ml vials (Wockhardt UK Ltd) | 5 |
| 17731 | Penmix 50/50 100iu/ml Injection (Novo Nordisk Ltd) | 5 |
| 17770 | Glucagon novo 10mg Injection (Novo Nordisk Ltd) | 0 |
| 17809 | Humaject m4 100iu/ml M4 pen (Eli Lilly and Company Ltd) | 5 |
| 18220 | Pioglitazone 15mg / Metformin 850mg tablets | 2 |
| 18224 | Humalog 100units/ml solution for injection 10ml vials (Eli Lilly and Company Ltd) | 5 |
| 18461 | Insulin zinc mixed human 100units/ml suspension for injection 10ml vials | 5 |
| 18590 | Insulin isophane bovine 100units/ml suspension for injection 10ml vials | 5 |
| 18592 | Insulin soluble bovine 100units/ml solution for injection 10ml vials | 5 |
| 18593 | Humalog Mix50 100units/ml suspension for injection 3ml cartridges (Eli Lilly and Company Ltd) | 5 |
| 18931 | Insulin zinc crystalline human 100units/ml suspension for injection 10ml vials | 5 |
| 19336 | Tolazamide 100mg Tablet | 2 |
| 19425 | Glucagon 10mg Injection | 0 |
| 19472 | Actos 45mg tablets (Takeda UK Ltd) | 2 |
| 19491 | Apidra 100units/ml solution for injection 10ml vials (Sanofi) | 5 |
| 19513 | Humulin M3 100units/ml suspension for injection 10ml vials (Eli Lilly and Company Ltd) | 5 |
| 19658 | Glurenorm 30mg tablets (Sanofi) | 2 |
| 19877 | Insulin aspart 100units/ml solution for injection 3ml pre-filled disposable devices | 5 |
| 19878 | Insulin isophane biphasic human 30/70 100units/ml suspension for injection 3ml pre-filled disposable devices | 5 |
| 20287 | Actos 15mg tablets (Takeda UK Ltd) | 2 |
| 20422 | Insuman comb 15 100iu/ml Injection (Aventis Pharma) | 5 |
| 20889 | Actos 30mg tablets (Takeda UK Ltd) | 2 |
| 20995 | Hypurin Porcine 30/70 Mix 100units/ml suspension for injection 3ml cartridges (Wockhardt UK Ltd) | 5 |
| 21110 | Insulin biphasic isophane human prb 50:50; 100 units/ml Injection | 5 |
| 21232 | Insulin isophane biphasic human 30/70 100units/ml suspension for injection 10ml vials | 5 |
| 21235 | Humulin S 100units/ml solution for injection 10ml vials (Eli Lilly and Company Ltd) | 5 |
| 21347 | Penmix 40/60 100iu/ml Injection (Novo Nordisk Ltd) | 5 |
| 21374 | Insulin biphasic isophane human prb 40:60; 100 units/ml Injection | 5 |
| 21395 | Insulin biphasic isophane human pyr 40:60; 100 units/ml Injection | 5 |
| 21422 | Insulin isophane biphasic human 40/60 100units/ml suspension for injection 3ml cartridges | 5 |
| 21424 | Glibenclamide 5mg/5ml oral suspension | 2 |
| 21489 | Tolanase 250mg Tablet (Pharmacia Ltd) | 2 |
| 21554 | Insuman comb 50 100iu/ml Injection (Aventis Pharma) | 5 |
| 21564 | Gliclazide 80mg tablets (Wockhardt UK Ltd) | 2 |
| 21583 | Apidra 100units/ml solution for injection 3ml pre-filled OptiSet pen (Sanofi) | 5 |
| 21590 | Insulin glulisine 100units/ml solution for injection 3ml pre-filled disposable devices | 5 |
| 21832 | Diabetamide 5mg tablets (Ashbourne Pharmaceuticals Ltd) | 2 |
| 21892 | Diaglyk 80mg tablets (Ashbourne Pharmaceuticals Ltd) | 2 |
| 22058 | Pur-in mix 15/85 Injection (C P Pharmaceuticals Ltd) | 5 |
| 22145 | Tolanase 100mg Tablet (Pharmacia Ltd) | 2 |
| 22155 | Humaject m5 100iu/ml M5 pen (Eli Lilly and Company Ltd) | 5 |
| 22239 | Glucagon lilly 1mg Injection (Eli Lilly and Company Ltd) | 0 |
| 22697 | Insulin isophane biphasic human 50/50 100units/ml suspension for injection 1.5ml cartridges | 5 |
| 22858 | Acetohexamide 500mg tablets | 2 |
| 22945 | Insuman rapid 100iu/ml Injection (Aventis Pharma) | 5 |
| 22983 | Insuman Rapid 100units/ml solution for injection 3ml cartridges (Sanofi) | 5 |
| 23099 | Insulin aspart biphasic 30/70 100units/ml suspension for injection 3ml pre-filled disposable devices | 5 |
| 23231 | Hypurin Bovine Neutral 100units/ml solution for injection 3ml cartridges (Wockhardt UK Ltd) | 5 |
| 23945 | Starlix 60mg tablets (Novartis Pharmaceuticals UK Ltd) | 2 |
| 23992 | Insuman Basal 100units/ml suspension for injection 3ml pre-filled OptiSet pen (Sanofi) | 5 |
| 23993 | Insuman Rapid 100units/ml solution for injection 3ml pre-filled OptiSet pen (Sanofi) | 5 |
| 24002 | Insuman Comb 25 100units/ml suspension for injection 5ml vials (Sanofi) | 5 |
| 24593 | Neutral insulin bovine 100unit/ml Injection | 5 |
| 24795 | Insulin aspart biphasic 30/70 100units/ml suspension for injection 3ml cartridges | 5 |
| 24800 | Hypurin Porcine 30/70 Mix 100units/ml suspension for injection 10ml vials (Wockhardt UK Ltd) | 5 |
| 24846 | Pur-in neutral 100unit/ml Injection (C P Pharmaceuticals Ltd) | 5 |
| 24848 | Glymidine sodium 500mg Tablet | 2 |
| 24993 | Insuman Comb 25 100units/ml suspension for injection 3ml cartridges (Sanofi) | 5 |
| 25133 | Insuman Comb 25 100units/ml suspension for injection 3ml pre-filled OptiSet pen (Sanofi) | 5 |
| 25479 | Insulin soluble porcine 100units/ml solution for injection 3ml cartridges | 5 |
| 25636 | Libanil 2.5mg Tablet (Approved Prescription Services Ltd) | 2 |
| 25678 | Glucamet 500mg Tablet (Opus Pharmaceuticals Ltd) | 1 |
| 25735 | Insulin isophane biphasic human 20/80 100units/ml suspension for injection 3ml cartridges | 5 |
| 25736 | Insulin isophane biphasic human 10/90 100units/ml suspension for injection 3ml cartridges | 5 |
| 25812 | Insulin isophane human 100units/ml suspension for injection 3ml pre-filled disposable devices | 5 |
| 25887 | Eudemine 50mg tablets (Focus Pharmaceuticals Ltd) | 0 |
| 26060 | Insulin lispro 100units/ml solution for injection 10ml vials | 5 |
| 26098 | Hypurin Porcine Neutral 100units/ml solution for injection 10ml vials (Wockhardt UK Ltd) | 5 |
| 26118 | Dimelor 500mg Tablet (Eli Lilly and Company Ltd) | 2 |
| 26218 | Calabren 5mg Tablet (Berk Pharmaceuticals Ltd) | 2 |
| 26258 | Glucamet 850mg Tablet (Opus Pharmaceuticals Ltd) | 1 |
| 26403 | Pur-in mix 25/75 Injection (C P Pharmaceuticals Ltd) | 5 |
| 26498 | Insulin zinc suspension mixed bovine and porcine 100unit/ml Injection | 5 |
| 26621 | Insulin soluble human crb 100iu/ml Injection | 5 |
| 27125 | Starlix 180mg tablets (Novartis Pharmaceuticals UK Ltd) | 2 |
| 27177 | Insulin biphasic lispro human prb 50:50; 100 units/ml Injection | 5 |
| 27280 | Insulin isophane biphasic porcine 30/70 100units/ml suspension for injection 10ml vials | 5 |
| 27396 | Insulin soluble porcine 100units/ml solution for injection 10ml vials | 5 |
| 27402 | Insulin soluble human 100units/ml solution for injection 10ml vials | 5 |
| 27461 | Insuman Basal 100units/ml suspension for injection 3ml cartridges (Sanofi) | 5 |
| 27501 | Orabet 500mg Tablet (Lagap) | 1 |
| 27614 | Penmix 30/70 100iu/ml Injection (Novo Nordisk Ltd) | 5 |
| 27969 | Glymese 250mg Tablet (DDSA Pharmaceuticals Ltd) | 2 |
| 28096 | Insulin isophane biphasic human 50/50 100units/ml suspension for injection 3ml cartridges | 5 |
| 28101 | Insulin glulisine 100units/ml solution for injection 10ml vials | 5 |
| 28183 | Hypurin Porcine Isophane 100units/ml suspension for injection 10ml vials (Wockhardt UK Ltd) | 5 |
| 28185 | Insulin lispro biphasic 25/75 100units/ml suspension for injection 3ml cartridges | 5 |
| 28442 | Insulin glulisine 100unit/ml Solution for injection | 5 |
| 28588 | Hypurin Bovine Isophane 100units/ml suspension for injection 3ml cartridges (Wockhardt UK Ltd) | 5 |
| 28708 | Malix 2.5mg Tablet (Lagap) | 2 |
| 29326 | Glipizide 5mg tablets (Mylan Ltd) | 2 |
| 29567 | Insulin aspart 100units/ml solution for injection 10ml vials | 5 |
| 29837 | Insulin biphasic isophane human prb 25:75; 100 units/ml Injection | 5 |
| 29939 | Gliclazide 80mg tablets (Mylan Ltd) | 2 |
| 29953 | Apidra 100units/ml solution for injection 3ml OptiClik cartridges (Sanofi) | 5 |
| 30209 | Actrapid mc 100unit/ml Injection (Arun Products Ltd) | 5 |
| 30236 | Isophane insulin 100iu/ml Injection | 5 |
| 30316 | Metformin with pioglitazone 850mg + 15mg Tablet | 2 |
| 30460 | Malix 5mg Tablet (Lagap) | 2 |
| 30686 | Insulin isophane porcine 100units/ml suspension for injection 3ml cartridges | 5 |
| 30819 | Insuman Comb 15 100units/ml suspension for injection 3ml pre-filled OptiSet pen (Sanofi) | 5 |
| 31077 | Competact 15mg/850mg tablets (Takeda UK Ltd) | 2 |
| 31146 | Metsol 500mg/5ml oral solution (Kappin Ltd) | 1 |
| 31205 | Insuman Comb 50 100units/ml suspension for injection 3ml pre-filled OptiSet pen (Sanofi) | 5 |
| 31212 | Gliclazide 80mg tablets (Actavis UK Ltd) | 2 |
| 31258 | Insulin lispro biphasic 25/75 100units/ml suspension for injection 3ml pre-filled disposable devices | 5 |
| 31417 | Diazoxide 50mg/ml oral solution | 0 |
| 31465 | Exubera 1mg inhalation powder blisters (Pfizer Ltd) | 5 |
| 31467 | Exubera 3mg inhalation powder blisters (Pfizer Ltd) | 5 |
| 31474 | Libanil 5mg Tablet (Approved Prescription Services Ltd) | 2 |
| 33087 | Metformin 500mg tablets (Actavis UK Ltd) | 1 |
| 33167 | Insulin biphasic isophane human crb 25:75; 100 units/ml Injection | 5 |
| 33232 | Insulin isophane biphasic human 50/50 100units/ml suspension for injection 5ml vials | 5 |
| 33562 | Duclazide 80mg Tablet (Dumex Ltd) | 2 |
| 33673 | Tolbutamide 500mg tablets (Actavis UK Ltd) | 2 |
| 33674 | Metformin 850mg tablets (A A H Pharmaceuticals Ltd) | 1 |
| 33966 | Insulatard 100unit/ml Injection (Novo Nordisk Ltd) | 5 |
| 34004 | Metformin 500mg tablets (IVAX Pharmaceuticals UK Ltd) | 1 |
| 34020 | Metformin 850mg tablets (IVAX Pharmaceuticals UK Ltd) | 1 |
| 34031 | Monotard mc 100unit/ml Injection (Novo Nordisk Ltd) | 5 |
| 34097 | Human initard 50/50 100unit/ml Injection (Novo Nordisk Ltd) | 5 |
| 34135 | Metformin 500mg Tablet (M & A Pharmachem Ltd) | 1 |
| 34323 | Metformin 500mg tablets (A A H Pharmaceuticals Ltd) | 1 |
| 34399 | Gliclazide 80mg tablets (IVAX Pharmaceuticals UK Ltd) | 2 |
| 34504 | Metformin 500mg tablets (Wockhardt UK Ltd) | 1 |
| 34507 | Glibenclamide 2.5mg tablets (Wockhardt UK Ltd) | 2 |
| 34563 | Glibenclamide 5mg tablets (Wockhardt UK Ltd) | 2 |
| 34598 | Metformin 500mg tablets (Mylan Ltd) | 1 |
| 34676 | Glibenclamide 2.5mg tablets (A A H Pharmaceuticals Ltd) | 2 |
| 34697 | Metformin 850mg tablets (Wockhardt UK Ltd) | 1 |
| 34706 | Glibenclamide 2.5mg tablets (IVAX Pharmaceuticals UK Ltd) | 2 |
| 34742 | Metformin 850mg tablets (Teva UK Ltd) | 1 |
| 34802 | Glipizide 5mg tablets (IVAX Pharmaceuticals UK Ltd) | 2 |
| 34836 | Metformin 850mg tablets (Actavis UK Ltd) | 1 |
| 34917 | Metformin 500mg tablets (Teva UK Ltd) | 1 |
| 34932 | Gliclazide 80mg tablets (Genus Pharmaceuticals Ltd) | 2 |
| 34957 | Tolbutamide 500mg tablets (A A H Pharmaceuticals Ltd) | 2 |
| 35022 | Sitagliptin 100mg tablets | 2 |
| 35144 | Byetta 5micrograms/0.02ml solution for injection 1.2ml pre-filled disposable devices (AstraZeneca UK Ltd) | 2 |
| 35149 | Exenatide 10micrograms/0.04ml solution for injection 2.4ml pre-filled disposable devices | 2 |
| 35150 | Byetta 10micrograms/0.04ml solution for injection 2.4ml pre-filled disposable devices (AstraZeneca UK Ltd) | 2 |
| 35251 | Exenatide 5micrograms/0.02ml solution for injection 1.2ml pre-filled disposable devices | 2 |
| 35253 | Insuman Comb 50 100units/ml suspension for injection 3ml cartridges (Sanofi) | 5 |
| 35260 | Levemir InnoLet 100units/ml solution for injection 3ml pre-filled pen (Novo Nordisk Ltd) | 5 |
| 35462 | Januvia 100mg tablets (Merck Sharp & Dohme Ltd) | 2 |
| 35468 | Insuman Basal 100units/ml suspension for injection 5ml vials (Sanofi) | 5 |
| 35561 | Prandin 2mg tablets (Novo Nordisk Ltd) | 2 |
| 35701 | Insulin lispro biphasic 50/50 100units/ml suspension for injection 3ml pre-filled disposable devices | 5 |
| 35960 | Diazoxide 50mg/5ml oral suspension | 0 |
| 36031 | Insulin isophane biphasic porcine 30/70 100units/ml suspension for injection 3ml cartridges | 5 |
| 36066 | Insulin isophane bovine 100units/ml suspension for injection 3ml cartridges | 5 |
| 36146 | Insulin lispro biphasic 50/50 100units/ml suspension for injection 3ml cartridges | 5 |
| 36194 | Insulin isophane biphasic human 25/75 100units/ml suspension for injection 3ml cartridges | 5 |
| 36355 | Insulin human 1mg inhalation powder blisters | 5 |
| 36356 | Insulin human 3mg inhalation powder blisters | 5 |
| 36430 | Insulin soluble human 100units/ml solution for injection 3ml pre-filled disposable devices | 5 |
| 36513 | Velosulin cartridge 100unit/ml Injection (Novo Nordisk Ltd) | 5 |
| 36774 | Prandin 1mg tablets (Novo Nordisk Ltd) | 2 |
| 36853 | Lantus 100units/ml solution for injection 3ml pre-filled SoloStar pen (Sanofi) | 5 |
| 36856 | Gliclazide 80mg tablets (Sandoz Ltd) | 2 |
| 36920 | Apidra 100units/ml solution for injection 3ml pre-filled SoloStar pen (Sanofi) | 5 |
| 36948 | Prandin 0.5mg tablets (Novo Nordisk Ltd) | 2 |
| 37617 | Rosiglitazone 2mg tablet | 2 |
| 37874 | Vildagliptin 50mg / Metformin 850mg tablets | 2 |
| 37875 | Vildagliptin 50mg tablets | 2 |
| 37902 | Vildagliptin 50mg / Metformin 1g tablets | 2 |
| 38355 | Metformin 750mg modified-release tablets | 1 |
| 38400 | Glucophage SR 750mg tablets (Merck Serono Ltd) | 1 |
| 38422 | Isophane 100iu/ml Injection (Celltech Pharma Europe Ltd) | 5 |
| 38542 | Hypo-fit Oral solution (Arctic Medical Ltd) | 0 |
| 38551 | Eucreas 50mg/1000mg tablets (Novartis Pharmaceuticals UK Ltd) | 2 |
| 38941 | TRUEone testing strips (Nipro Diagnostics (UK) Ltd) | 0 |
| 38986 | Humalog KwikPen 100units/ml solution for injection 3ml pre-filled pen (Eli Lilly and Company Ltd) | 5 |
| 39006 | Humalog Mix25 KwikPen 100units/ml suspension for injection 3ml pre-filled pen (Eli Lilly and Company Ltd) | 5 |
| 39086 | Humalog Mix50 KwikPen 100units/ml suspension for injection 3ml pre-filled pen (Eli Lilly and Company Ltd) | 5 |
| 39149 | Galvus 50mg tablets (Novartis Pharmaceuticals UK Ltd) | 2 |
| 39203 | Eucreas 50mg/850mg tablets (Novartis Pharmaceuticals UK Ltd) | 2 |
| 39306 | Sucrose with glucose & fructose oral solution | 0 |
| 39560 | Bolamyn SR 500mg tablets (Teva UK Ltd) | 1 |
| 39598 | Metformin 1g modified-release tablets | 1 |
| 39729 | Glucophage SR 1000mg tablets (Merck Serono Ltd) | 1 |
| 39956 | Diazoxide 250mg/5ml oral suspension | 0 |
| 39988 | Metformin 500mg oral powder sachets sugar free | 1 |
| 40007 | Glucophage 1000mg oral powder sachets (Merck Serono Ltd) | 1 |
| 40110 | Glucophage 500mg oral powder sachets (Merck Serono Ltd) | 1 |
| 40233 | Metformin 1g oral powder sachets sugar free | 1 |
| 40348 | Dextrogel 40% gel (Neoceuticals Ltd) | 0 |
| 40365 | Glimepiride 1mg tablets (Actavis UK Ltd) | 2 |
| 40425 | Nazdol MR 30mg tablets (Teva UK Ltd) | 2 |
| 40642 | Victoza 6mg/ml solution for injection 3ml pre-filled pen (Novo Nordisk Ltd) | 2 |
| 40693 | Liraglutide 6mg/ml solution for injection 3ml pre-filled disposable devices | 2 |
| 41120 | Insulin isophane biphasic human 50/50 100units/ml suspension for injection 3ml pre-filled disposable devices | 5 |
| 41204 | Saxagliptin 5mg tablets | 2 |
| 41431 | Onglyza 5mg tablets (AstraZeneca UK Ltd) | 2 |
| 41558 | Glibenclamide 5mg tablets (Teva UK Ltd) | 2 |
| 41559 | Glibenclamide 5mg tablets (A A H Pharmaceuticals Ltd) | 2 |
| 41593 | Glibenclamide 2.5mg tablets (Teva UK Ltd) | 2 |
| 41834 | Insulin zinc suspension lente 100iu/ml Injection (Celltech Pharma Europe Ltd) | 5 |
| 41838 | Mobile cassette (Roche Diabetes Care Ltd) | 0 |
| 41959 | Penject 100unit/ml Injection device (Hypoguard Ltd) | 5 |
| 42161 | Orabet 500mg Tablet (Sandoz Ltd) | 1 |
| 42395 | Humalog Mix25 100units/ml suspension for injection 10ml vials (Eli Lilly and Company Ltd) | 5 |
| 42677 | Diazoxide 250mg/5ml oral solution | 0 |
| 42790 | Gliclazide 80mg Tablet (Merck Generics (UK) Ltd) | 2 |
| 42954 | Insulin isophane biphasic human 25/75 100units/ml suspension for injection 5ml vials | 5 |
| 43065 | Gliclazide 40mg tablets | 2 |
| 43270 | Metformin 500mg/5ml oral solution sugar free (Rosemont Pharmaceuticals Ltd) | 1 |
| 43465 | Zicron 40mg tablets (Bristol Laboratories Ltd) | 2 |
| 43619 | Metformin 1g / Sitagliptin 50mg tablets | 2 |
| 43684 | Janumet 50mg/1000mg tablets (Merck Sharp & Dohme Ltd) | 2 |
| 43950 | Humulin I KwikPen 100units/ml suspension for injection 3ml pre-filled pen (Eli Lilly and Company Ltd) | 5 |
| 43953 | Insulin lispro biphasic 25/75 100units/ml suspension for injection 10ml vials | 5 |
| 43991 | Humulin M3 KwikPen 100units/ml suspension for injection 3ml pre-filled pen (Eli Lilly and Company Ltd) | 5 |
| 44250 | Metformin 500mg/5ml Oral solution (Hillcross Pharmaceuticals Ltd) | 1 |
| 44251 | Insulin zinc suspension mixed porcine 100unit/ml Injection | 5 |
| 44304 | Glyconon 500mg Tablet (DDSA Pharmaceuticals Ltd) | 2 |
| 44378 | Insulin isophane biphasic human 25/75 100units/ml suspension for injection 3ml pre-filled disposable devices | 5 |
| 44473 | Edicil MR 30mg tablets (Ratiopharm UK Ltd) | 2 |
| 44480 | Insuman Comb 25 100units/ml suspension for injection 3ml pre-filled SoloStar pen (Sanofi) | 5 |
| 44738 | Niddaryl 1mg tablets (Dee Pharmaceuticals Ltd) | 2 |
| 45158 | Insuman Comb 15 100units/ml suspension for injection 3ml cartridges (Sanofi) | 5 |
| 45215 | Gliclazide 80mg Tablet (Neo Laboratories Ltd) | 2 |
| 45581 | Metabet SR 500mg tablets (Morningside Healthcare Ltd) | 1 |
| 45775 | Saxagliptin 2.5mg tablets | 2 |
| 45821 | Onglyza 2.5mg tablets (AstraZeneca UK Ltd) | 2 |
| 45831 | Dacadis MR 30mg tablets (Mylan Ltd) | 2 |
| 46001 | Insuman Basal 100units/ml suspension for injection 3ml pre-filled SoloStar pen (Sanofi) | 5 |
| 46458 | Exenatide 2mg powder and solvent for suspension for injection vials | 2 |
| 46469 | Bydureon 2mg powder and solvent for suspension for prolonged-release injection vials (AstraZeneca UK Ltd) | 2 |
| 46665 | Linagliptin 5mg tablets | 2 |
| 46666 | NovoRapid FlexTouch 100units/ml solution for injection 3ml pre-filled pen (Novo Nordisk Ltd) | 5 |
| 46692 | Dextro Energy tablets blackcurrant (Ceuta Healthcare Ltd) | 0 |
| 46716 | Trajenta 5mg tablets (Boehringer Ingelheim Ltd) | 2 |
| 46720 | Glucose tablets | 0 |
| 46927 | Tolbutamide 500mg tablets (Teva UK Ltd) | 2 |
| 46989 | Metabet SR 1000mg tablets (Morningside Healthcare Ltd) | 1 |
| 47025 | Dextro Energy tablets tropical (Ceuta Healthcare Ltd) | 0 |
| 47048 | Dextro Energy tablets orange (Ceuta Healthcare Ltd) | 0 |
| 47074 | Gliclazide 80mg/5ml oral suspension | 2 |
| 47173 | GSF-Syrup oral gel 18g sachets | 0 |
| 47262 | Dextro Energy tablets original (Ceuta Healthcare Ltd) | 0 |
| 47293 | FineTouch testing tips (Terumo UK Ltd) | 0 |
| 47360 | Neutral insulin 100unit/ml Injection (Celltech Pharma Europe Ltd) | 5 |
| 48239 | Contour Next testing strips (Bayer Diagnostics Manufacturing Ltd) | 0 |
| 50692 | iCare Advanced testing strips (iCare Medical UK Ltd) | 0 |
| 58581 | GlucoRx FinePoint hypodermic insulin needles for pre-filled / reusable pen injectors screw on 5mm/31gauge (GlucoRx Ltd) | 0 |

## COPD

The code list for Medcodes is based on the code list in Forbes, H. J. *Understanding risk factors for herpes zoster and postherpetic neuralgia in UK primary care: investigations to inform vaccine policy*. Diss. London School of Hygiene & Tropical Medicine, 2016.

### COPD Medcodes

| **Medcode** | **Description** |
| --- | --- |
| 794 | emphysema |
| 998 | chronic obstructive airways disease |
| 1001 | chronic obstructive pulmonary disease |
| 1446 | acute exacerbation of chronic obstructive airways disease |
| 3243 | chronic bronchitis |
| 5710 | chronic obstructive airways disease nos |
| 7884 | chron obstruct pulmonary dis wth acute exacerbation, unspec |
| 9520 | chronic obstructive pulmonary disease monitoring |
| 9876 | severe chronic obstructive pulmonary disease |
| 10802 | moderate chronic obstructive pulmonary disease |
| 10863 | mild chronic obstructive pulmonary disease |
| 10980 | centrilobular emphysema |
| 11019 | admit copd emergency |
| 11150 | mucopurulent chronic bronchitis |
| 11287 | chronic obstructive pulmonary disease annual review |
| 12166 | other specified chronic obstructive airways disease |
| 14798 | emphysematous bronchitis |
| 15157 | chronic bronchitis nos |
| 15626 | chronic catarrhal bronchitis |
| 18476 | copd follow-up |
| 18501 | copd self-management plan given |
| 18621 | chronic obstructive pulmonary disease follow-up |
| 18792 | chronic obstructive pulmonary disease monitoring admin |
| 19003 | emergency copd admission since last appointment |
| 19106 | copd accident and emergency attendance since last visit |
| 21061 | chronic obstruct pulmonary dis with acute lower resp infectn |
| 23492 | chronic bullous emphysema nos |
| 24248 | mixed simple and mucopurulent chronic bronchitis |
| 25603 | simple chronic bronchitis |
| 26018 | chronic obstructive pulmonary disease monitoring by nurse |
| 26306 | chronic bullous emphysema |
| 27819 | obstructive chronic bronchitis |
| 28755 | chronic obstructive pulmonary disease monitoring 1st letter |
| 33450 | emphysema nos |
| 34202 | chronic obstructive pulmonary disease monitoring 2nd letter |
| 34215 | chronic obstructive pulmonary disease monitoring 3rd letter |
| 37247 | chronic obstructive pulmonary disease nos |
| 37371 | chronic obstructive pulmonary disease monitoring due |
| 37959 | fetid chronic bronchitis |
| 38074 | chronic obstructive pulmonary disease monitor phone invite |
| 40159 | purulent chronic bronchitis |
| 42258 | chronic obstructive pulmonary disease monitoring verb invite |
| 42624 | coad follow-up |
| 44525 | obstructive chronic bronchitis nos |
| 45770 | chronic obstructive pulmonary disease disturbs sleep |
| 45771 | chronic obstructive pulmonary disease does not disturb sleep |
| 45777 | chronic obstructive pulmonary disease clini management plan |
| 45998 | chronic obstructive pulmonary disease monitoring by doctor |
| 46036 | multiple copd emergency hospital admissions |
| 46578 | panlobular emphysema |
| 56860 | segmental bullous emphysema |
| 60188 | giant bullous emphysema |
| 61118 | simple chronic bronchitis nos |
| 61513 | mucopurulent chronic bronchitis nos |
| 64721 | chronic emphysema due to chemical fumes |
| 65733 | [x]other specified chronic obstructive pulmonary disease |
| 66043 | other chronic bronchitis |
| 67040 | other specified chronic obstructive pulmonary disease |
| 68066 | other chronic bronchitis nos |
| 93568 | very severe chronic obstructive pulmonary disease |
| 96931 | at risk of chronic obstructive pulmonary diseas exacerbation |
| 98283 | copd structured smoking assessment declined - enh serv admin |
| 98284 | refer copd structured smoking assessment - enhanc serv admin |
| 99536 | bullous emphysema with collapse |
| 99948 | copd patient unsuitable for pulmonary rehab - enh serv admin |
| 101042 | issue of chronic obstructive pulmonary disease rescue pack |
| 102685 | chronic obstructive pulmonary disease 3 monthly review |
| 103007 | chronic obstructive pulmonary disease 6 monthly review |
| 103400 | referred for copd structured smoking assessment |

### COPD ICD-10 codes

| **ICD-10** | **Description** |
| --- | --- |
| J44.0 | Chronic obstructive pulmonary disease with acute lower respiratory infection |
| J44.1 | Chronic obstructive pulmonary disease with acute exacerbation, unspecified |
| J44.8 | Other specified chronic obstructive pulmonary disease |
| J44.9 | Chronic obstructive pulmonary disease, unspecified |

# Life style factors

## Smoking

The code list for Medcodes is based on the code list in Forbes, H. J. *Understanding risk factors for herpes zoster and postherpetic neuralgia in UK primary care: investigations to inform vaccine policy*. Diss. London School of Hygiene & Tropical Medicine, 2016.

For tobacco smoking status two sources of information will be used:

- Clinical events identified by Read codes
- Additional events identified by “enttype” = 4 (variable “data1” with lookup table YND and eventdate from Clinical linked by “patid” and “adid” variables)

### Smoking Medcodes

The table below includes all the medcodes used in CPRD to identify the smoking status.

| **Medcode** | **Description** | **Smoking_Status** |
| --- | --- | --- |
| 33 | Never smoked tobacco | Non smoker |
| 54 | Tobacco consumption | Smoker |
| 60 | Current non-smoker | Non smoker |
| 90 | Ex smoker | Ex smoker |
| 93 | Cigarette smoker | Smoker |
| 776 | Stopped smoking | Ex smoker |
| 1822 | Very heavy smoker - 40+cigs/d | Smoker |
| 1823 | Smoker | Smoker |
| 1878 | Moderate smoker - 10-19 cigs/d | Smoker |
| 2111 | Health ed. - smoking | Smoker |
| 3568 | Heavy smoker - 20-39 cigs/day | Smoker |
| 6359 | Nicotine withdrawal | Ex smoker |
| 7130 | Stop smoking monitoring admin. | Ex smoker |
| 7622 | Smoking cessation advice | Smoker |
| 9045 | Advice on smoking | Smoker |
| 9833 | Nicotine replacement therapy | Smoker |
| 10184 | Pregnancy smoking advice | Smoker |
| 10211 | Smoking cessation milestones | Smoker |
| 10558 | Current smoker | Smoker |
| 10742 | Referral to stop-smoking clinic | Smoker |
| 10898 | Smoking free weeks | Smoker |
| 11356 | Seen by smoking cessation advisor | Smoker |
| 11527 | DNA - Did not attend smoking cessation clinic | Smoker |
| 11713 | Pack years | Smoker |
| 11788 | Non-smoker | Non smoker |
| 12240 | Trying to give up smoking | Smoker |
| 12878 | Date ceased smoking | Ex smoker |
| 12941 | Occasional smoker | Smoker |
| 12942 | Smoker - amount smoked | Smoker |
| 12943 | Cigar smoker | Smoker |
| 12944 | Light smoker - 1-9 cigs/day | Smoker |
| 12945 | Rolls own cigarettes | Smoker |
| 12946 | Ex-smoker - amount unknown | Ex smoker |
| 12947 | Pipe smoker | Smoker |
| 12951 | Smoking restarted | Smoker |
| 12952 | Smoking started | Smoker |
| 12953 | Attends stop smoking monitor. | Ex smoker |
| 12954 | [V]Tobacco use | Smoker |
| 12955 | Ex-moderate smoker (10-19/day) | Ex smoker |
| 12956 | Ex-heavy smoker (20-39/day) | Ex smoker |
| 12957 | Ex-light smoker (1-9/day) | Ex smoker |
| 12958 | Trivial smoker - < 1 cig/day | Smoker |
| 12959 | Ex-very heavy smoker (40+/day) | Ex smoker |
| 12960 | Tobacco consumption NOS | Smoker |
| 12961 | Ex-trivial smoker (<1/day) | Ex smoker |
| 12962 | Tobacco consumption unknown | Smoker |
| 12963 | Cigar consumption | Smoker |
| 12964 | Keeps trying to stop smoking | Smoker |
| 12965 | Cigarette consumption | Smoker |
| 12966 | Smoking reduced | Smoker |
| 12967 | Pipe tobacco consumption | Smoker |
| 13351 | Passive smoker | Smoker |
| 16717 | Smokers' cough | Smoker |
| 18573 | Referral to smoking cessation advisor | Smoker |
| 18926 | Lifestyle advice regarding smoking | Smoker |
| 19485 | Stop smoking monitor.chck done | Ex smoker |
| 19488 | Ex cigar smoker | Ex smoker |
| 21637 | Stop smoking monitor admin.NOS | Ex smoker |
| 24529 | Nicotine replacement therapy refused | Smoker |
| 25106 | Nicotine replacement therapy provided free | Smoker |
| 26096 | Smokes drugs | Smoker |
| 26470 | Ex pipe smoker | Ex smoker |
| 28834 | Anti-smoking monitoring admin. | Ex smoker |
| 30423 | Thinking about stopping smoking | Smoker |
| 30762 | Not interested in stopping smoking | Smoker |
| 31114 | Ready to stop smoking | Smoker |
| 32083 | Stop smoking clinic admin. | Ex smoker |
| 32572 | Over the counter nicotine replacement therapy | Smoker |
| 32687 | Tobacco dependence | Smoker |
| 34126 | Negotiated date for cessation of smoking | Smoker |
| 35055 | [V]Tobacco abuse counselling | Smoker |
| 38112 | Smoking cessation programme start date | Smoker |
| 40417 | Stop smoking monitor default | Smoker |
| 40418 | Refuses stop smoking monitor | Smoker |
| 41042 | Smoking cessation advice provided by community pharmacist | Smoker |
| 41979 | Smoking restarted | Smoker |
| 42288 | Pack years | Smoker |
| 42722 | Stop smoking monitor 1st lettr | Smoker |
| 43433 | Toxic effect of tobacco and nicotine | Smoker |
| 46300 | Cigarette pack-years | Smoker |
| 46321 | Reason for restarting smoking | Smoker |
| 47273 | Motives for smoking scale | Smoker |
| 49418 | RFS - Reasons for smoking scale | Smoker |
| 53101 | Stop smoking monitor verb.inv. | Smoker |
| 56144 | [X]Mental and behav dis due to use of tobacco: harmful use | Smoker |
| 57639 | Bupropion refused | Smoker |
| 58597 | Stop smoking monitor phone inv | Smoker |
| 59866 | Reasons for smoking scale | Smoker |
| 60720 | Stop smoking monitor 2nd lettr | Smoker |
| 61905 | [X]Mental and behavioural disorder due to use of tobacco | Smoker |
| 62686 | Minutes from waking to first tobacco consumption | Smoker |
| 63299 | FTND - Fagerstrom test for nicotine dependence | Smoker |
| 63666 | Fagerstrom test for nicotine dependence | Smoker |
| 63717 | Bupropion contraindicated | Smoker |
| 63901 | Stop smoking monitoring delete | Ex smoker |
| 66387 | Stop smoking monitor 3rd lettr | Smoker |
| 66409 | Nicotine replacement therapy contraindicated | Smoker |
| 67178 | Nicotine replacement therapy provided by community pharmacis | Smoker |
| 68658 | Tobacco dependence NOS | Smoker |
| 70746 | Tobacco dependence, continuous | Smoker |
| 72700 | [V]Personal history of tobacco abuse | Ex smoker |
| 72706 | Tobacco dependence in remission | Ex smoker |
| 74907 | Smoking cessation therapy | Smoker |
| 81440 | Nicotine replacement therapy using nicotine patches | Smoker |
| 85247 | Nicotine replacement therapy using nicotine inhalator | Smoker |
| 85975 | Nicotine replacement therapy using nicotine gum | Smoker |
| 89464 | Nicotine replacement therapy using nicotine lozenges | Smoker |
| 90522 | Smoking cessation therapy NOS | Smoker |
| 91513 | Occasions for smoking scale | Smoker |
| 91708 | Other specified smoking cessation therapy | Smoker |
| 94958 | Smoking cessation drug therapy | Smoker |
| 95610 | Tobacco dependence, unspecified | Smoker |
| 96992 | Smoking cessation - enhanced services administration | Ex smoker |
| 97210 | Ex-cigarette smoker | Ex smoker |
| 97973 | Maternal tobacco abuse | Smoker |
| 98137 | Brief intervention for smoking cessation | Smoker |
| 98154 | Referral to NHS stop smoking service | Smoker |
| 98177 | Non-smoker annual review - enhanced services administration | Non smoker |
| 98245 | Stop smoking face to face follow-up | Ex smoker |
| 98283 | COPD structured smoking assessment declined - enh serv admin | Smoker |
| 98284 | Refer COPD structured smoking assessment - enhanc serv admin | Smoker |
| 98347 | Current smoker annual review - enhanced services admin | Smoker |
| 98447 | Ex-smoker annual review - enhanced services administration | Ex smoker |
| 98493 | Smoking cessatn monitor template complet - enhanc serv admin | Ex smoker |
| 99838 | Recently stopped smoking | Ex smoker |
| 100099 | Smoking cessation advice declined | Smoker |
| 100495 | Ex roll-up cigarette smoker | Ex smoker |
| 100963 | Ex-smoker annual review | Ex smoker |
| 101210 | Consent given for smoking cessation data sharing | Ex smoker |
| 101325 | Declin cons follow-up evaluation after smoking cess interven | Ex smoker |
| 101338 | Failed attempt to stop smoking | Smoker |
| 101385 | Consent given for follow-up by smoking cessation team | Ex smoker |
| 101519 | [X]Mental and behav dis due to use tobacco: withdrawal state | Smoker |
| 101634 | Consent given follow-up after smoking cessation intervention | Ex smoker |
| 101764 | Practice based smoking cessation programme start date | Smoker |
| 101851 | Declined consent for follow-up by smoking cessation team | Ex smoker |
| 101854 | Declined consent for smoking cessation data sharing | Ex smoker |
| 101878 | Non-smoker annual review | Non smoker |
| 102361 | Referral for smoking cessation service offered | Smoker |
| 102951 | Lost to smoking cessation follow-up | Smoker |
| 103760 | COPD structured smoking assessment declined | Smoker |
| 103955 | Asthma trigger - tobacco smoke | Smoker |
| 104310 | Current smoker annual review | Smoker |

## BMI

For BMI two sources of information will be used:

- Clinical events identified by Read codes
- Additional events identified by “enttype” = 13 (numeric variable “data3” with eventdate from Clinical linked by “patid” and “adid” variables). The numeric value is further classified using the instructions at the table below.

| **BMI** | **Recorded values (data3)** |
| --- | --- |
| Unknown | Missing |
| Underweight | < 20 |
| Normal weight | >= 20 and < 25 |
| Overweight | >= 25 and < 30 |
| Obese | >= 30 |

### BMI Medcodes

| **Medcode** | **Description** | **BMI_Status** |
| --- | --- | --- |
| 26473 | O/E - weight > 20% below ideal | Underweight |
| 29029 | O/E -weight 10-20% below ideal | Underweight |
| 23376 | O/E - weight within 10% ideal | Normal |
| 16404 | O/E - weight 10-20% over ideal | Overweight |
| 2839 | O/E - overweight | Overweight |
| 32974 | O/E - weight > 20% over ideal | Overweight |
| 7984 | O/E - obese | Obese |
| 126 | O/E - Underweight | Underweight |
| 28946 | Body Mass Index normal K/M2 | Normal |
| 28937 | Body Mass Index high K/M2 | Overweight |
| 32914 | Body Mass Index low K/M2 | Underweight |
| 9015 | Body mass index index 25-29 - overweight | Overweight |
| 13278 | Body mass index 30+ - obesity | Obese |
| 24496 | Body mass index less than 20 | Underweight |
| 22556 | Body mass index 40+ - severely obese | Obese |
| 44291 | Body mass index 20-24 - normal | Normal |
| 8041 | Weight monitoring | Overweight |
| 26355 | Target weight discussed | Overweight |
| 3355 | Weight loss advised | Overweight |
| 26415 | Ideal weight discussed | Overweight |
| 25061 | Ideal weight | Normal |
| 8481 | Wants to lose weight | Overweight |
| 29721 | Difficulty maintaining weight loss | Overweight |
| 40640 | Target weight | Normal |
| 32879 | Weight management programme offered | Overweight |
| 26182 | Weight management plan started | Overweight |
| 35407 | Weight management plan completed | Normal |
| 43472 | Target weight reached | Normal |
| 102150 | Risk health associ overweight and obesity, at increased risk | Overweight |
| 102514 | Risk health associated overweight and obesity, at high risk | Overweight |
| 8964 | Weight reducing diet | Overweight |
| 94788 | Weight gain diet | Underweight |
| 6713 | Patient advised to lose weight | Overweight |
| 11443 | Actions to lose weight | Overweight |
| 11763 | Patient advised about weight-reducing diet | Overweight |
| 8304 | Dietary advice for weight reduction | Overweight |
| 61420 | Dietary advice for weight gain | Underweight |
| 43375 | Dietary advice for weight loss | Overweight |
| 12445 | Patient advised to lose weight | Overweight |

# Contraindications or precautions for zoster vaccination

## Immunocompromised

Patients are considered immunocompromised from their 1^st^ record onwards if they have any record of acute/chronic leukaemia and lymphomas, HIV/AIDS, cellular immune deficiencies or haematological malignancies. Patients are considered immunocompromised for 2 years after each record of haematopoietic stem cell transplant.

### Acute and chronic leukaemias and lymphomas/ Haematological malignancies

The codes used to identify acute and chronic leukemias and lymphomas, and hematological malignancies are in the same list.

#### HM Medcodes

| **Medcode** | **Description** |
| --- | --- |
| 10411 | Waldenstrom's macroglobulinaemia |
| 39244 | Urine paraprotein level |
| 31794 | Unspecified B-cell non-Hodgkin's lymphoma |
| 101465 | Unspec malig neop lymphoid/histiocytic of multiple sites |
| 93384 | Unspec malig neop lymphoid/histiocytic of intrathoracic node |
| 71609 | Unspec malig neop lymphoid/histiocytic nodes inguinal/leg |
| 64427 | Unspec malig neop lymphoid/histiocytic lymph node head/neck |
| 107638 | Unspec malig neop lymphoid/histiocytic lymph node axilla/arm |
| 103353 | Unspec malig neop lymphoid/histiocytic intra-abdominal nodes |
| 105762 | Unifocal Langerhans-cell histiocytosis |
| 90201 | T-zone lymphoma |
| 95630 | True histiocytic lymphoma |
| 22726 | Transfusion of bone marrow |
| 65777 | Thrombocytic leukaemia |
| 107643 | T-cell prolymphocytic leukaemia |
| 105085 | T/NK-cell lymphoma |
| 105925 | Subcutaneous panniculitic T-cell lymphoma |
| 104475 | Subacute myelomonocytic leukaemia |
| 63475 | Subacute myeloid leukaemia |
| 101606 | Subacute monocytic leukaemia |
| 72774 | Subacute lymphoid leukaemia |
| 54793 | Subacute leukaemia NOS |
| 104418 | Solitary plasmacytoma |
| 19028 | Solitary myeloma |
| 104386 | Small cell B-cell lymphoma |
| 100532 | Sezary's disease NOS |
| 35014 | Sezary's disease |
| 13801 | Serum paraprotein level |
| 7654 | Secondary malignant neoplasm of bone and bone marrow |
| 54420 | Second stage peripheral stem cell infusion |
| 105335 | Sarcoma of dendritic cells |
| 99951 | Reticulosarcoma or lymphosarcoma NOS |
| 60242 | Reticulosarcoma of unspecified site |
| 95058 | Reticulosarcoma of spleen |
| 71031 | Reticulosarcoma of lymph nodes of head, face and neck |
| 70374 | Reticulosarcoma of intra-abdominal lymph nodes |
| 99240 | Reticulosarcoma NOS |
| 1481 | Reticulosarcoma |
| 22890 | Refractory anaemia without sideroblasts, so stated |
| 10817 | Refractory anaemia with sideroblasts |
| 44420 | Refractory anaemia with excess of blasts with transformation |
| 23875 | Refractory anaemia with excess of blasts |
| 31586 | Prolymphocytic leukaemia |
| 12265 | Primary thrombocythaemia |
| 36790 | Primary polycythaemia |
| 105286 | Primary cutaneous CD30-positive T-cell proliferations |
| 2481 | Polycythaemia vera |
| 5542 | Polycythaemia rubra vera |
| 102672 | Polycythaemia irradiation |
| 66617 | Polyclonal hypergammaglobulinaemia NOS |
| 17775 | Polyclonal hypergammaglobulinaemia |
| 21329 | Plasmacytoma NOS |
| 38321 | Plasmacytoma NOS |
| 39187 | Plasma cell leukaemia |
| 12464 | Peripheral T-cell lymphoma |
| 28232 | Peripheral blood stem cell graft |
| 12386 | Paraproteinaemia NOS |
| 31576 | Other types of follicular non-Hodgkin's lymphoma |
| 106063 | Other types of follicular lymphoma |
| 99887 | Other specified reticulosarcoma or lymphosarcoma |
| 30632 | Other specified leukaemia NOS |
| 37272 | Other specified leukaemia |
| 3451 | Other paraproteinaemias |
| 66089 | Other myeloid leukaemia NOS |
| 103645 | Other monocytic leukaemia NOS |
| 99015 | Other monocytic leukaemia |
| 104934 | Other mature T/NK-cell lymphoma |
| 33333 | Other malignant neoplasm of lymphoid and histiocytic tissue |
| 38331 | Other lymphoid leukaemia NOS |
| 49725 | Other lymphoid leukaemia |
| 34692 | Other leukaemia of unspecified cell type |
| 64567 | Other immunoproliferative neoplasms |
| 104484 | Other classical Hodgkin lymphoma |
| 99413 | Other and unspecified leukaemia NOS |
| 94174 | Other and unspecified leukaemia |
| 44318 | Oth and unspecif peripheral & cutaneous T-cell lymphomas |
| 60433 | Osteoporosis in multiple myelomatosis |
| 104391 | Non-Hodgkin lymphoma |
| 106867 | Non-follicular lymphoma |
| 106884 | Nonfollicular lymphoma |
| 3604 | Non - Hodgkin's lymphoma |
| 105841 | Nodular sclerosis classical Hodgkin lymphoma |
| 66327 | Nodular lymphoma of unspecified site |
| 58082 | Nodular lymphoma of lymph nodes of multiple sites |
| 94995 | Nodular lymphoma of lymph nodes of inguinal region and leg |
| 45264 | Nodular lymphoma of lymph nodes of head, face and neck |
| 105203 | Nodular lymphoma of intrathoracic lymph nodes |
| 92068 | Nodular lymphoma of intra-abdominal lymph nodes |
| 65701 | Nodular lymphoma NOS |
| 5179 | Nodular lymphoma (Brill - Symmers disease) |
| 104895 | Nodular lymphocyte predominant Hodgkin lymphoma |
| 59663 | Neoplasm of uncertain behaviour of plasma cells |
| 45414 | Neoplasm of uncertain behaviour of histiocytic and mast cell |
| 72551 | Neoplasm of uncertain behaviour of blood |
| 20609 | Neop uncertain behaviour other lymphatic/haematopoietic tiss |
| 20440 | Myelomonocytic leukaemia |
| 15211 | Myelomatosis |
| 43312 | Myeloma - solitary |
| 70724 | Myeloid sarcoma |
| 33344 | Myeloid leukaemia NOS |
| 7176 | Myeloid leukaemia |
| 14927 | Myelodysplasia |
| 95949 | Mycosis fungoides of unspecified site |
| 95012 | Mycosis fungoides of lymph nodes of multiple sites |
| 72714 | Mycosis fungoides of lymph nodes of inguinal region and leg |
| 96379 | Mycosis fungoides of lymph nodes of axilla and upper limb |
| 91674 | Mycosis fungoides of intra-abdominal lymph nodes |
| 38005 | Mycosis fungoides NOS |
| 12006 | Mycosis fungoides |
| 37182 | Multiple myeloma and immunoproliferative neoplasms |
| 4944 | Multiple myeloma |
| 105792 | Multifocal multisystemic dissem Langerhans-cell histiocytosi |
| 95715 | Mucosa-associated lymphoma |
| 93342 | Monocytic leukaemia NOS |
| 35875 | Monocytic leukaemia |
| 15883 | Monoclonal paraproteinaemia |
| 7586 | Monoclonal gammopathy |
| 67700 | Monoblastic leukaemia |
| 57671 | Megakaryocytic leukaemia |
| 105038 | Mediastinal (thymic) large B-cell lymphoma |
| 4661 | Mastocytoma NOS |
| 103900 | Mast cell malignancy of unspecified site |
| 31324 | Mast cell malignancy of lymph nodes of multiple sites |
| 100615 | Mast cell malignancy of lymph nodes inguinal region and leg |
| 65721 | Mast cell leukaemia |
| 104620 | Mantle cell lymphoma |
| 95545 | Maltoma |
| 62437 | Malignant reticulosis |
| 22158 | Malignant plasma cell neoplasm, extramedullary plasmacytoma |
| 65434 | Malignant neoplasms of lymphoid and histiocytic tissue NOS |
| 15036 | Malignant mast cell tumours |
| 89657 | Malignant mast cell tumour NOS |
| 17887 | Malignant lymphoma otherwise specified |
| 57427 | Malignant lymphoma NOS of unspecified site |
| 60092 | Malignant lymphoma NOS of spleen |
| 15504 | Malignant lymphoma NOS of lymph nodes of multiple sites |
| 50696 | Malignant lymphoma NOS of lymph nodes of head, face and neck |
| 34089 | Malignant lymphoma NOS of lymph nodes of axilla and arm |
| 63105 | Malignant lymphoma NOS of lymph node inguinal region and leg |
| 72725 | Malignant lymphoma NOS of intrathoracic lymph nodes |
| 71262 | Malignant lymphoma NOS of intrapelvic lymph nodes |
| 42579 | Malignant lymphoma NOS of intra-abdominal lymph nodes |
| 12335 | Malignant lymphoma NOS |
| 15027 | Malignant lymphoma NOS |
| 58962 | Malignant immunoproliferative small intestinal disease |
| 69497 | Malignant histiocytosis of unspecified site |
| 94415 | Malignant histiocytosis of lymph nodes head, face and neck |
| 65642 | Malignant histiocytosis of intra-abdominal lymph nodes |
| 58871 | Malignant histiocytosis NOS |
| 44267 | Malignant histiocytosis |
| 71625 | Lymphosarcoma of unspecified site |
| 103245 | Lymphosarcoma of spleen |
| 104790 | Lymphosarcoma of lymph nodes of multiple sites |
| 100352 | Lymphosarcoma of lymph nodes of inguinal region and leg |
| 71238 | Lymphosarcoma of lymph nodes of head, face and neck |
| 62380 | Lymphosarcoma of intrathoracic lymph nodes |
| 64670 | Lymphosarcoma of intra-abdominal lymph nodes |
| 63723 | Lymphosarcoma NOS |
| 72197 | Lymphosarcoma cell leukaemia |
| 41369 | Lymphosarcoma and reticulosarcoma |
| 27416 | Lymphosarcoma |
| 26111 | Lymphomatoid papulosis |
| 71672 | Lymphoma stage IV |
| 32240 | Lymphoma stage III |
| 94935 | Lymphoma stage II |
| 60918 | Lymphoma stage I |
| 38914 | Lymphoid leukaemia NOS |
| 19372 | Lymphoid leukaemia |
| 95792 | Lymphoid and histiocytic malignancy NOS |
| 57737 | Lymphoepithelioid lymphoma |
| 106597 | Lymphocyte-rich classical Hodgkin lymphoma |
| 104412 | Lymphoblastic (diffuse) lymphoma |
| 4222 | Lymphatic leukaemia |
| 65122 | Leukaemic reticuloendotheliosis of unspecified sites |
| 73777 | Leukaemic reticuloendotheliosis NOS |
| 5137 | Leukaemic reticuloendotheliosis |
| 27330 | Leukaemic reticuloendotheliosis |
| 65123 | Leukaemic reticuloend of intra-abdominal lymph nodes |
| 25191 | Leukaemia of unspecified cell type |
| 4250 | Leukaemia NOS |
| 102715 | Letterer-Siwe disease of unspecified sites |
| 54083 | Letterer-Siwe disease of lymph nodes of multiple sites |
| 102158 | Letterer-Siwe disease of intrathoracic lymph nodes |
| 47204 | Letterer-Siwe disease NOS |
| 34926 | Letterer-Siwe disease |
| 46042 | Lambda light chain myeloma |
| 43552 | Kahler's disease |
| 105069 | Juvenile myelomonocytic leukaemia |
| 43450 | Immunoproliferative neoplasm or myeloma NOS |
| 70716 | Immunoproliferative neoplasm |
| 17386 | Idiopathic thrombocythaemia |
| 31560 | Idiopathic thrombocythaemia |
| 71142 | Hodgkin's, lymphocytic-histiocytic predominance unspec site |
| 106911 | Hodgkin's, lymphocytic-histiocytic predominance of spleen |
| 29876 | Hodgkin's, lymphocytic-histiocytic predominance NOS |
| 104743 | Hodgkin's, lymphocytic-histiocytic pred of multiple sites |
| 68330 | Hodgkin's, lymphocytic-histiocytic pred of head, face, neck |
| 92245 | Hodgkin's, lymphocytic-histiocytic pred intrathoracic nodes |
| 95338 | Hodgkin's, lymphocytic-histiocytic pred intrapelvic nodes |
| 73532 | Hodgkin's, lymphocytic-histiocytic pred intra-abdominal node |
| 93951 | Hodgkin's, lymphocytic-histiocytic pred inguinal and leg |
| 68039 | Hodgkin's sarcoma of lymph nodes of axilla and upper limb |
| 64036 | Hodgkin's sarcoma |
| 100423 | Hodgkin's paragranuloma of lymph nodes of head, face, neck |
| 98840 | Hodgkin's paragranuloma of intra-abdominal lymph nodes |
| 65489 | Hodgkin's paragranuloma |
| 19140 | Hodgkin's nodular sclerosis of lymph nodes of multiple sites |
| 65483 | Hodgkin's nodular sclerosis of lymph nodes of axilla and arm |
| 67506 | Hodgkin's nodular sclerosis of intrathoracic lymph nodes |
| 61149 | Hodgkin's nodular sclerosis of intra-abdominal lymph nodes |
| 55303 | Hodgkin's nodular sclerosis of head, face and neck |
| 94407 | Hodgkin's mixed cellularity of lymph nodes head, face, neck |
| 58684 | Hodgkin's mixed cellularity of intrathoracic lymph nodes |
| 95049 | Hodgkin's lymphocytic depletion of unspecified site |
| 107032 | Hodgkin's lymphocytic depletion lymph nodes multiple sites |
| 63625 | Hodgkin's lymphocytic depletion lymph nodes axilla and arm |
| 98909 | Hodgkin's granuloma of lymph nodes of head, face and neck |
| 44196 | Hodgkin's granuloma |
| 57225 | Hodgkin's disease, nodular sclerosis of unspecified site |
| 105472 | Hodgkin's disease, nodular sclerosis of spleen |
| 63054 | Idiopathic thrombocythaemia |
| 29178 | Hodgkin's disease, nodular sclerosis |
| 97863 | Hodgkin's disease, mixed cellularity of unspecified site |
| 94005 | Hodgkin's disease, mixed cellularity NOS |
| 49605 | Hodgkin's disease, mixed cellularity |
| 38939 | Hodgkin's disease, lymphocytic-histiocytic predominance |
| 101715 | Hodgkin's disease, lymphocytic depletion of spleen |
| 101530 | Hodgkin's disease, lymphocytic depletion NOS |
| 67703 | Hodgkin's disease, lymphocytic depletion |
| 61662 | Hodgkin's disease NOS, unspecified site |
| 94279 | Hodgkin's disease NOS of spleen |
| 97746 | Hodgkin's disease NOS of lymph nodes of multiple sites |
| 59778 | Hodgkin's disease NOS of lymph nodes of head, face and neck |
| 91900 | Hodgkin's disease NOS of lymph nodes of axilla and arm |
| 99012 | Hodgkin's disease NOS of lymph nodes inguinal region and leg |
| 59755 | Hodgkin's disease NOS of intrathoracic lymph nodes |
| 42461 | Hodgkin's disease NOS |
| 53397 | Hodgkin's disease NOS |
| 2462 | Hodgkin's disease |
| 106349 | Hodgkin lymphoma NOS |
| 104291 | Hodgkin lymphoma |
| 44617 | HIV disease resulting in Burkitt's lymphoma |
| 66367 | HIV dis resulting oth types of non-Hodgkin's lymphoma |
| 51708 | HIV dis reslt/oth mal neopl/lymph,h'matopoetc+reltd tissu |
| 4870 | Histiocytosis X (acute, progressive) |
| 29789 | Histiocytic tumour NOS |
| 105083 | Histiocytic sarcoma |
| 87335 | Hairy cell leukaemia |
| 17177 | H/O: * leukaemia |
| 39629 | Granulocytic sarcoma |
| 100078 | Graft of cord blood to bone marrow |
| 99067 | Gamma heavy chain disease |
| 28639 | Follicular non-Hodgkin's small cleaved cell lymphoma |
| 21549 | Follicular non-Hodgkin's lymphoma |
| 49262 | Follicular non-Hodgkin's large cell lymphoma |
| 70842 | Follicular non-Hodg mixed sml cleavd & lge cell lymphoma |
| 17182 | Follicular lymphoma NOS |
| 105020 | Follicular lymphoma grade 3a |
| 107166 | Follicular lymphoma grade 3 |
| 105095 | Follicular lymphoma grade 2 |
| 105889 | Follicular lymphoma grade 1 |
| 104152 | Follicular lymphoma |
| 63236 | First stage peripheral stem cell infusion |
| 105966 | Extranod marg zone B-cell lymphom mucosa-assoc lymphoid tiss |
| 11950 | Essential (haemorrhagic) thrombocythaemia |
| 105709 | Enteropathy-associated T-cell lymphoma |
| 19647 | Electrophoresis: paraprotein |
| 50668 | Diffuse non-Hodgkin's small cell (diffuse) lymphoma |
| 39798 | Diffuse non-Hodgkin's lymphoma, unspecified |
| 65180 | Diffuse non-Hodgkin's lymphoma undifferentiated (diffuse) |
| 17460 | Diffuse non-Hodgkin's lymphoblastic (diffuse) lymphoma |
| 101114 | Diffuse non-Hodgkin's large cell lymphoma |
| 53551 | Diffuse non-Hodgkin's immunoblastic (diffuse) lymphoma |
| 70509 | Diffuse non-Hodgkin's centroblastic lymphoma |
| 50695 | Diffuse non-Hodgkin mixed sml & lge cell (diffuse) lymphoma |
| 102594 | Diffuse large B-cell lymphoma |
| 106969 | Diffuse follicle centre lymphoma |
| 27340 | Di Guglielmo's disease |
| 46423 | Cystosarcoma phyllodes |
| 104862 | Cutaneous T-cell lymphoma |
| 102783 | Chronic neutrophilic leukaemia |
| 22050 | Chronic myelomonocytic leukaemia |
| 105957 | Chronic myeloid leukaemia, BCR/ABL positive |
| 27520 | Chronic myeloid leukaemia NOS |
| 10726 | Chronic myeloid leukaemia |
| 27458 | Chronic monocytic leukaemia |
| 8625 | Chronic lymphoid leukaemia |
| 107017 | Chronic lymphocytic leukaemia of B-cell type |
| 27790 | Chronic lymphatic leukaemia |
| 16416 | Chronic leukaemia NOS |
| 31701 | Chronic granulocytic leukaemia |
| 100786 | Chronic eosinophilic leukaemia |
| 52327 | Chloroma |
| 92380 | Burkitt's lymphoma of lymph nodes of inguinal region and leg |
| 59115 | Burkitt's lymphoma of lymph nodes of head, face and neck |
| 100006 | Burkitt's lymphoma of intrathoracic lymph nodes |
| 97577 | Burkitt's lymphoma of intra-abdominal lymph nodes |
| 71304 | Burkitt's lymphoma NOS |
| 21402 | Burkitt's lymphoma |
| 52946 | Bone marrow: myeloma cells |
| 73173 | Bone marrow suppres.-irradiat. |
| 105375 | Blastic NK-cell lymphoma |
| 104328 | B-cell chronic lymphocytic leukaemia |
| 104325 | B-cell acute lymphoblastic leukaemia |
| 86063 | Autologous peripheral blood stem cell transplant |
| 107236 | Atypical chronic myeloid leukaemia, BCR/ABL negative |
| 105636 | Angioimmunoblastic T-cell lymphoma |
| 105559 | Anaplastic large cell lymphoma, ALK-positive |
| 105955 | Anaplastic large cell lymphoma, ALK-negative |
| 101350 | Alpha heavy chain disease |
| 93362 | Allograft of cord blood to bone marrow |
| 89920 | Allogeneic peripheral blood stem cell transplant |
| 104939 | Adult T-cell lymphoma/leukaemia (HTLV-1-associated) |
| 37461 | Adult T-cell leukaemia |
| 27664 | Acute promyelocytic leukaemia |
| 50858 | Acute panmyelosis |
| 61500 | Acute myelomonocytic leukaemia |
| 4413 | Acute myeloid leukaemia |
| 104788 | Acute myeloblastic leukaemia |
| 19974 | Acute monocytic leukaemia |
| 4251 | Acute lymphoid leukaemia |
| 4072 | Acute leukaemia NOS |
| 42539 | Acute erythraemia and erythroleukaemia |
| 63375 | [X]Unspecified B-cell non-Hodgkin's lymphoma |
| 67518 | [X]Other types of follicular non-Hodgkin's lymphoma |
| 98596 | [X]Other types of diffuse non-Hodgkin's lymphoma |
| 64336 | [X]Other specified types of non-Hodgkin's lymphoma |
| 89329 | [X]Other specified leukaemias |
| 61693 | [X]Other myeloid leukaemia |
| 89762 | [X]Other monocytic leukaemia |
| 102688 | [X]Other malignant immunoproliferative diseases |
| 67029 | [X]Other lymphoid leukaemia |
| 65165 | [X]Other leukaemia of unspecified cell type |
| 43415 | [X]Other Hodgkin's disease |
| 105025 | [X]Oth spcf mal neoplsm/lymphoid,haematopoietic+rltd tissue |
| 8649 | [X]Non-Hodgkin's lymphoma, unspecified type |
| 7940 | [X]Non-Hodgkin's lymphoma NOS |
| 45143 | [X]Myelodysplastic syndrome, unspecified |
| 40740 | [X]Malignant neoplasms of lymphoid, haematopoietic and rela |
| 72500 | [X]Mal neoplasm/lymphoid,haematopoietic+related tissu,unspcf |
| 69767 | [X]HIV disease resulting in other non-Hodgkin's lymphoma |
| 64515 | [X]Diffuse non-Hodgkin's lymphoma, unspecified |
| 72204 | [V]Personal history other lymphatic/haematopoietic neoplasm |
| 94597 | [V]Personal history of lymphoid leukaemia |
| 36693 | [V]Personal history of leukaemia |
| 40561 | [V]Personal history of Hodgkin's disease |
| 53477 | [V]Follow-up examination after chemotherapy for leukaemia |
| 9172 | [M]Waldenstrom's macroglobulinaemia |
| 57544 | [M]True histiocytic lymphoma |
| 69299 | [M]Thrombocytic leukaemia |
| 106483 | [M]Subacute myeloid leukaemia |
| 72179 | [M]Subacute leukaemia NOS |
| 63570 | [M]Stem cell leukaemia |
| 73135 | [M]Solitary myeloma |
| 97756 | [M]Sezary's disease |
| 49825 | [M]Reticulum cell sarcoma NOS |
| 106137 | [M]Reticulosarcomas |
| 100544 | [M]Reticulosarcoma, nodular |
| 72433 | [M]Reticulosarcoma NOS |
| 72241 | [M]Prolymphocytic lymphosarcoma |
| 46048 | [M]Prolymphocytic leukaemia |
| 39490 | [M]Plasmacytic myeloma |
| 43459 | [M]Plasma cell tumours |
| 99702 | [M]Plasma cell tumour, malignant |
| 64068 | [M]Plasma cell tumour NOS |
| 31671 | [M]Plasma cell myeloma |
| 64618 | [M]Plasma cell leukaemias |
| 62330 | [M]Other myeloid leukaemia NOS |
| 3371 | [M]Non Hodgkins lymphoma |
| 66694 | [M]Naegeli-type monocytic leukaemia |
| 24317 | [M]Myelosis NOS |
| 53647 | [M]Myelomatosis |
| 3672 | [M]Myeloma NOS |
| 96893 | [M]Myeloid sarcoma |
| 35697 | [M]Myeloid leukaemias |
| 71850 | [M]Myeloid leukaemia NOS |
| 7799 | [M]Myelodysplastic syndrome |
| 99695 | [M]Mycosis fungoides NOS |
| 46967 | [M]Mycosis fungoides |
| 95464 | [M]Mycosis fungoides |
| 18744 | [M]Multiple myeloma |
| 102164 | [M]Monostotic myeloma |
| 31749 | [M]Monocytoid B-cell lymphoma |
| 73088 | [M]Monocytic leukaemia NOS |
| 66267 | [M]Miscellaneous reticuloendothelial neoplasms |
| 73066 | [M]Miscellaneous leukaemias |
| 63973 | [M]Microglioma |
| 72222 | [M]Megakaryocytic leukaemia |
| 70740 | [M]Malignant reticulosis |
| 58015 | [M]Malignant lymphomatous polyposis |
| 71117 | [M]Malignant lymphoma, undifferentiated cell type NOS |
| 46931 | [M]Malignant lymphoma, stem cell type |
| 46877 | [M]Malignant lymphoma, small lymphocytic NOS |
| 31726 | [M]Malignant lymphoma, small cleaved cell, diffuse |
| 51680 | [M]Malignant lymphoma, small cell, noncleaved, diffuse |
| 16460 | [M]Malignant lymphoma, non Hodgkin's type |
| 63699 | [M]Malignant lymphoma, nodular NOS |
| 71652 | [M]Malignant lymphoma, mixed small and large cell, diffuse |
| 41754 | [M]Malignant lymphoma, lymphoplasmacytoid type |
| 69980 | [M]Malignant lymphoma, lymphocytic, well differentiated NOS |
| 72196 | [M]Malignant lymphoma, lymphocytic, poorly different NOS |
| 71619 | [M]Malignant lymphoma, large cell, noncleaved, diffuse |
| 33869 | [M]Malignant lymphoma, large cell, diffuse NOS |
| 63994 | [M]Malignant lymphoma, large cell, cleaved, diffuse |
| 48253 | [M]Malignant lymphoma, immunoblastic type |
| 41841 | [M]Malignant lymphoma, follicular centre cell NOS |
| 23711 | [M]Malignant lymphoma, diffuse NOS |
| 69301 | [M]Malignant lymphoma, convoluted cell type NOS |
| 39906 | [M]Malignant lymphoma, centrocytic |
| 98961 | [M]Malignant lymphoma, centroblastic-centrocytic, follicular |
| 68964 | [M]Malignant lymphoma, centroblastic-centrocytic, diffuse |
| 97852 | [M]Malignant lymphoma, centroblastic type, follicular |
| 60275 | [M]Malignant lymphoma, centroblastic type NOS |
| 36114 | [M]Malignant lymphoma NOS |
| 63239 | [M]Malignant histiocytosis |
| 61251 | [M]Malign lymphoma,lymphocytic,intermediate differn, diffuse |
| 106970 | [M]Malig lymphoma, lymphocytic, well differentiated,nodular |
| 51852 | [M]Malig lymphoma, lymphocytic, intermediate different NOS |
| 66603 | [M]Malig lymphoma, follicular centre cell, non-cleaved NOS |
| 58953 | [M]Malig lymp,follicular centre cell,noncleaved,follicular |
| 39883 | [M]Malig lymp, follicular centre cell, cleaved, follicular |
| 99655 | [M]Lymphosarcoma NOS |
| 49131 | [M]Lymphomatous tumour, benign |
| 17178 | [M]Lymphomas, NOS or diffuse |
| 20437 | [M]Lymphomas, nodular or follicular |
| 40513 | [M]Lymphoma, nodular or follicular NOS |
| 51895 | [M]Lymphoma, diffuse or NOS |
| 1483 | [M]Lymphoma NOS |
| 48155 | [M]Lymphoid leukaemias |
| 12146 | [M]Lymphoid leukaemia NOS |
| 101429 | [M]Lymphogranuloma, malignant |
| 60504 | [M]Lymphocytic lymphosarcoma NOS |
| 21463 | [M]Lymphocytic lymphoma NOS |
| 52591 | [M]Lymphoblastoma NOS |
| 67203 | [M]Lymphoblastic lymphosarcoma NOS |
| 34352 | [M]Lymphoblastic lymphoma NOS |
| 20635 | [M]Lymphatic leukaemia |
| 40420 | [M]Leukaemias unspecified |
| 4637 | [M]Leukaemias |
| 59929 | [M]Leukaemia unspecified, NOS |
| 41734 | [M]Leukaemia NOS |
| 42297 | [M]Leukaemia NOS |
| 59593 | [M]Letterer - Siwe disease |
| 89230 | [M]Hodgkin's granuloma |
| 96183 | [M]Hodgkin's disease,lymphocytic depletion,diffuse fibrosis |
| 99200 | [M]Hodgkin's disease, nodular sclerosis, cellular phase |
| 42198 | [M]Hodgkin's disease, nodular sclerosis NOS |
| 51285 | [M]Hodgkin's disease, mixed cellularity |
| 56041 | [M]Hodgkin's disease, lymphocytic predominance |
| 42769 | [M]Hodgkin's disease NOS |
| 61997 | [M]Hodgkin's disease NOS |
| 20710 | [M]Hodgkin's disease |
| 64343 | [M]Hodgkin,s disease, nodular sclerosis, mixed cellularity |
| 40508 | [M]Hodgkin,s disease, nodular sclerosis, lymphocytic predom |
| 31741 | [M]Hodgkin,s disease, nodular sclerosis, lymphocytic deplet |
| 31537 | [M]Hodgkin,s disease, lymphocytic predominance, nodular |
| 65584 | [M]Hodgkin,s disease, lymphocytic predominance, diffuse |
| 47330 | [M]Histiocytic medullary reticulosis |
| 5915 | [M]Hairy cell leukaemia |
| 98009 | [M]Granulocytic sarcoma |
| 37723 | [M]Granulocytic leukaemia NOS |
| 49253 | [M]Giant follicular lymphoma |
| 27562 | [M]Follicular lymphosarcoma NOS |
| 46444 | [M]Erythroleukaemias |
| 100927 | [M]Erythroleukaemia NOS |
| 70935 | [M]Erythroleukaemia |
| 57713 | [M]Eosinophilic leukaemias |
| 71377 | [M]Eosinophilic leukaemia |
| 48049 | [M]Chronic myelomonocytic leukaemia |
| 52942 | [M]Chronic myeloid leukaemia |
| 41500 | [M]Chronic lymphoid leukaemia |
| 31750 | [M]Chronic leukaemia NOS |
| 93944 | [M]Chloroma |
| 50928 | [M]Burkitt's cell leukaemia |
| 64947 | [M]Brill - Symmers' disease |
| 64963 | [M]Blastic leukaemia |
| 22071 | [M]Blast cell leukaemia |
| 106197 | [M]Basophilic leukaemia |
| 27965 | [M]AngiocentricT-cell lymphoma |
| 72310 | [M]Aleukaemic leukaemia NOS |
| 29335 | [M]Adult T-cell leukaemia/lymphoma |
| 3710 | [M]Adenolymphoma |
| 57316 | [M]Acute promyelocytic leukaemia |
| 45768 | [M]Acute progressive histiocytosis X |
| 102764 | [M]Acute panmyelosis |
| 46263 | [M]Acute myelomonocytic leukaemia |
| 54585 | [M]Acute myeloid leukaemia |
| 37487 | [M]Acute myelofibrosis |
| 49327 | [M]Acute megakaryoblastic leukaemia |
| 37410 | [M]Acute lymphoid leukaemia |
| 6316 | [M]Acute leukaemia NOS |
| 40766 | [M] Peripheral T-cell lymphoma NOS |
| 31492 | [M] Monocytoid B-cell lymphoma |
| 10395 | [M] Monoclonal gammopathy |
| 18383 | [M] Large cell lymphoma |
| 52593 | [M] Gamma heavy chain disease |
| 16774 | [M] Cutaneous lymphoma |
| 54190 | [M] Angioimmunoblastic lymphadenopathy |
| 61146 | [M] Angiocentric immunoproliferative lesion |
| 26135 | [M] Alpha heavy chain disease |

#### HM ICD-10 codes

| **ICD-10** | **Description** |
| --- | --- |
| C81.0 | Nodular lymphocyte predominant Hodgkin lymphoma |
| C81.1 | Nodular sclerosis Hodgkin lymphoma |
| C81.2 | Mixed cellularity Hodgkin lymphoma |
| C81.3 | Lymphocyte depleted Hodgkin lymphoma |
| C81.4 | Lymphocyte-rich Hodgkin lymphoma |
| C81.7 | Other Hodgkin lymphoma |
| C81.9 | Hodgkin lymphoma, unspecified |
| C81.9 | Hodgkin lymphoma, unspecified, lymph nodes of multiple sites |
| C81.9 | Hodgkin lymphoma, unspecified, extranodal and solid organ sites |
| C82.0 | Follicular lymphoma grade I |
| C82.1 | Follicular lymphoma grade II |
| C82.2 | Follicular lymphoma grade III, unspecified |
| C82.2 | Follicular lymphoma grade III, unspecified, unspecified site |
| C82.3 | Follicular lymphoma grade IIIa |
| C82.4 | Follicular lymphoma grade IIIb |
| C82.5 | Diffuse follicle center lymphoma |
| C82.6 | Cutaneous follicle center lymphoma |
| C82.9 | Follicular lymphoma, unspecified |
| C83.0 | Small cell B-cell lymphoma |
| C83.1 | Mantle cell lymphoma |
| C83.3 | Diffuse large B-cell lymphoma |
| C83.5 | Lymphoblastic (diffuse) lymphoma |
| C83.7 | Burkitt lymphoma |
| C83.8 | Other non-follicular lymphoma |
| C83.9 | Non-follicular (diffuse) lymphoma, unspecified |
| C84.0 | Mycosis fungoides |
| C84.1 | Sezary disease |
| C84.4 | Peripheral T-cell lymphoma, not classified |
| C84.6 | Anaplastic large cell lymphoma, ALK-positive |
| C84.7 | Anaplastic large cell lymphoma, ALK-negative |
| C84.9 | Mature T/NK-cell lymphomas, unspecified |
| C85.1 | Unspecified B-cell lymphoma |
| C85.2 | Mediastinal (thymic) large B-cell lymphoma |
| C85.9 | Non-Hodgkin lymphoma, unspecified |
| C85.9 | Non-Hodgkin lymphoma, unspecified, extranodal and solid organ sites |
| C86.0 | Extranodal NK/T-cell lymphoma, nasal type |
| C86.1 | Hepatosplenic T-cell lymphoma |
| C86.2 | Enteropathy-type (intestinal) T-cell lymphoma |
| C86.3 | Subcutaneous panniculitis-like T-cell lymphoma |
| C86.4 | Blastic NK-cell lymphoma |
| C86.5 | Angioimmunoblastic T-cell lymphoma |
| C86.6 | Primary cutaneous CD30-positive T-cell proliferations |
| C88.0 | Waldenstrom macroglobulinemia |
| C88.2 | Heavy chain disease |
| C88.3 | Immunoproliferative small intestinal disease |
| C88.4 | Extranodal marginal zone B-cell lymphoma of mucosa-associated lymphoid tissue [MALT-lymphoma] |
| C88.9 | Malignant immunoproliferative disease, unspecified |
| C90.0 | Multiple myeloma |
| C90.1 | Plasma cell leukemia |
| C90.2 | Extramedullary plasmacytoma |
| C90.3 | Solitary plasmacytoma |
| C91.0 | Acute lymphoblastic leukemia [ALL] |
| C91.1 | Chronic lymphocytic leukemia of B-cell type |
| C91.3 | Prolymphocytic leukemia of B-cell type |
| C91.4 | Hairy cell leukemia |
| C91.5 | Adult T-cell lymphoma/leukemia (HTLV-1-associated) |
| C91.6 | Prolymphocytic leukemia of T-cell type |
| C91.9 | Lymphoid leukemia, unspecified |
| C92.0 | Acute myeloblastic leukemia |
| C92.1 | Chronic myeloid leukemia, BCR/ABL-positive |
| C92.2 | Atypical chronic myeloid leukemia, BCR/ABL-negative |
| C92.3 | Myeloid sarcoma |
| C92.4 | Acute promyelocytic leukemia |
| C92.5 | Acute myelomonocytic leukemia |
| C92.6 | Acute myeloid leukemia with 11q23-abnormality |
| C92.9 | Myeloid leukemia, unspecified |
| C93.0 | Acute monoblastic/monocytic leukemia |
| C93.1 | Chronic myelomonocytic leukemia |
| C93.3 | Juvenile myelomonocytic leukemia |
| C93.9 | Monocytic leukemia, unspecified |
| C94.0 | Acute erythroid leukemia |
| C94.2 | Acute megakaryoblastic leukemia |
| C94.3 | Mast cell leukemia |
| C94.4 | Acute panmyelosis with myelofibrosis |
| C94.6 | Myelodysplastic disease, not classified |
| C95.0 | Acute leukemia of unspecified cell type |
| C95.1 | Chronic leukemia of unspecified cell type |
| C95.9 | Leukemia, unspecified |
| C96.0 | Multifocal and multisystemic (disseminated) Langerhans-cell histiocytosis |
| C96.2 | Malignant mast cell tumor |
| C96.4 | Sarcoma of dendritic cells (accessory cells) |
| C96.5 | Multifocal and unisystemic Langerhans-cell histiocytosis |
| C96.6 | Unifocal Langerhans-cell histiocytosis |
| C96.9 | Malignant neoplasm of lymphoid, hematopoietic and related tissue, unspecified |

### HIV/AIDS

The code list for Medcodes is based on the code list in Forbes, H. J. *Understanding risk factors for herpes zoster and postherpetic neuralgia in UK primary care: investigations to inform vaccine policy*. Diss. London School of Hygiene & Tropical Medicine, 2016.

#### HIV/AIDS Medcodes

| **Medcode** | **Description** |
| --- | --- |
| 2835 | HIV positive |
| 8281 | HIV disease resulting in wasting syndrome |
| 9130 | Human immunodeficiency virus infection |
| 23763 | AIDS carrier |
| 23951 | ZERIDAME SR tablets 150mg [ACTAVIS] |
| 24872 | [V]Asymptomatic human immunodeficency virus infection status |
| 27641 | HIV disease resulting in Pneumocystis carinii pneumonia |
| 27853 | HIV disease resulting in Kaposi's sarcoma |
| 33943 | Notification of AIDS |
| 36294 | Acquired human immunodeficiency virus infection syndrome NOS |
| 37006 | HIV disease resulting in mycobacterial infection |
| 41185 | [X]Dementia in human immunodef virus [HIV] disease |
| 43537 | HIV 1 nucleic acid detection |
| 44288 | [D]Laboratory evidence of human immunodeficiency virus [HIV] |
| 44617 | HIV disease resulting in Burkitt's lymphoma |
| 46442 | Retrovirus infection |
| 47632 | Herpes zoster with meningitis |
| 50076 | HIV disease resulting in multiple infections |
| 51708 | HIV dis reslt/oth mal neopl/lymph,h'matopoetc+reltd tissu |
| 53636 | Human immunodeficiency virus with neurological disease |
| 54423 | Retrovirus as cause of diseases classified to other chapters |
| 58857 | Acute human immunodeficiency virus infection |
| 58859 | Asymptomatic human immunodeficiency virus infection |
| 62854 | [X]Human immunodeficiency virus disease |
| 62891 | Human immunodeficiency virus with other clinical findings |
| 65117 | HIV disease resulting in lymphoid interstitial pneumonitis |
| 66367 | HIV dis resulting oth types of non-Hodgkin's lymphoma |
| 66368 | HIV disease resulting in cytomegaloviral disease |
| 67575 | HIV disease resulting in unspecified malignant neoplasm |
| 69766 | HIV infection with persistent generalised lymphadenopathy |
| 69767 | [X]HIV disease resulting in other non-Hodgkin's lymphoma |
| 70528 | Human immunodeficiency virus with secondary infection |
| 70869 | Human immunodeficiency virus with constitutional disease |
| 71450 | HIV disease resulting/unspcf infectious+parasitic disease |
| 72065 | HTLV 2 nucleic acid detection |
| 93642 | Human immunodeficiency virus RNA/DNA ratio |
| 96751 | [X]HIV disease result/haematological+immunologic abnorms,NEC |
| 96902 | Human immunodeficiency virus viral load by log rank |
| 96751 | [X]HIV disease result/haematological+immunologic abnorms,NEC |
| 98966 | Human immunodeficiency virus monitoring |
| 100769 | [X]Unspecified human immunodeficiency virus [HIV] disease |
| 101191 | Human immunodeficiency virus annual review |
| 101836 | Human immunodeficiency virus with secondary cancers |
| 102117 | [X]HIV disease resulting in multiple infections |
| 102252 | [X]HIV disease resulting in other specified conditions |
| 104134 | [X]HIV disease resulting/other infectious+parasitic diseases |
| 104466 | HIV disease complicating pregnancy childbirth puerperium |
| 104717 | HIV disease resulting in Pneumocystis jirovecii pneumonia |
| 105124 | [X]Retrovirus infections, not elsewhere classified |
| 105324 | HIV disease resulting in multiple malignant neoplasms |

#### HIV/AIDS ICD-10 codes

| **ICD-10** | **Description** |
| --- | --- |
| B20.0 | HIV disease resulting in mycobacterial infection |
| B20.1 | HIV disease resulting in other bacterial infections |
| B20.2 | HIV disease resulting in cytomegaloviral disease |
| B20.3 | HIV disease resulting in other viral infections |
| B20.4 | HIV disease resulting in candidiasis |
| B20.5 | HIV disease resulting in other mycoses |
| B20.6 | HIV disease resulting in Pneumocystis jirovecii pneumonia |
| B20.7 | HIV disease resulting in multiple infections |
| B20.8 | HIV disease resulting in other infectious and parasitic diseases |
| B20.9 | HIV disease resulting in unspecified infectious or parasitic disease |

### Cellular immune deficiencies

#### CID Medcodes

| **Medcode** | **Description** |
| --- | --- |
| 938 | Pancytopenia NOS |
| 3129 | Unspecified immunity deficiency |
| 4818 | Agranulocytosis |
| 5823 | Pancytopenia - acquired |
| 8548 | Selective IgA immunodeficiency |
| 10955 | Di George syndrome |
| 12717 | Disorders of the immune mechanism |
| 15137 | Hypogammaglobulinaemia NOS |
| 16295 | Deficiency of humoral immunity NOS |
| 18054 | Idiopathic neutropenia |
| 18700 | Selective IgG immunodeficiency |
| 18701 | Selective IgM immunodeficiency |
| 18781 | Chronic granulomatous disease |
| 21975 | Common variable immunodeficiency |
| 24802 | Disorder of the immune mechanism NOS |
| 30008 | Drug-induced neutropenia |
| 31275 | Pancytopenia with malformation |
| 31491 | Pancytopenia-dysmelia |
| 31541 | Severe combined immunodefiency with reticular dysgenesis |
| 32141 | Cyclical neutropenia |
| 35682 | Agammaglobulinaemia |
| 35719 | Neutropenia - drug induced |
| 36408 | Defects in the complement system |
| 38306 | Polymorphonuclear neutrophil disorder NOS |
| 39800 | Immunodeficiency associated+major defect, unspecified |
| 40310 | Neutropenia due to irradiation |
| 42439 | Thrombocytopenic eczema with immunodeficiency |
| 43653 | Dysgammaglobulinaemia NEC |
| 44147 | Deficiencies of humoral immunity |
| 48035 | T-lymphocyte deficiency |
| 48293 | Severe combined immunodeficiency |
| 48307 | Deficiencies of cell-mediated immunity |
| 49346 | Acquired neutropenia NEC |
| 49393 | Disorder involving the immune mechanism, unspecified |
| 49542 | Severe combined immunodef with low or normal B-cell numbers |
| 50526 | Major histocompatibility complex class I deficiency |
| 50665 | Predominantly T-cell immuno-deficiency NOS |
| 53113 | Congenital neutropenia |
| 53273 | Genetic leukocyte anomalies |
| 54589 | Acquired neutropenia in newborn |
| 54904 | Lymphocyte function antigen-1 defect |
| 56108 | Immunodef follow hereditary defect respon Epstein-Barr vir |
| 57161 | Congenital hypogammaglobulinaemia |
| 57322 | Common variable immunodeficiency |
| 60026 | Antibod def wth nr-norm imunoglob/or wth hyperimunoglobaemia |
| 60758 | Cellular immunity syndrome |
| 60880 | Congenital X-linked agammaglobulinaemia |
| 61326 | Pancytopenia with pancreatitis |
| 62236 | Combined immunity deficiency |
| 62328 | Combined immunity deficiency NOS |
| 62598 | Agammaglobulinaemia NEC |
| 63055 | Agranulocytosis NOS |
| 63204 | Idiopathic agranulocytosis |
| 64053 | Other specified disorders of the immune mechanism |
| 64300 | Other specified agranulocytosis |
| 65603 | Bruton's agammaglobulinaemia |
| 65617 | Immunodeficiency with short-limbed stature |
| 65903 | Acquired agranulocytosis NEC |
| 66049 | Congenital dysphagocytosis |
| 66073 | Severe combined immunodef with low T- and B-cell numbers |
| 66857 | Common variable immunodef wth autoantibod to B- or T-cells |
| 68440 | Other selective immunoglobulin deficiency |
| 69184 | Dysimmunoglobulinaemia NEC |
| 69373 | Immunodeficiency with IgM hypergammaglobulinaemia |
| 69854 | Other specified deficiency of humoral immunity |
| 91911 | Functional disorders of polymorphonuclear neutrophils |
| 92569 | Transient infant hypogammaglobulinaemia |
| 93892 | Com var immunodef with predom abn B-cell numbers and functns |
| 93936 | Purine nucleoside phosphorylase deficiency |
| 94120 | Swiss type agammaglobulinaemia |
| 96288 | Genetic leucocyte anomalies |
| 96380 | Primary splenic neutropenia |
| 103977 | Major histocompatibility complex class II deficiency |
| 104651 | Immune reconstitution syndrome |

#### CID ICD-10 codes

| **ICD-10** | **Description** |
| --- | --- |
| D70 | Neutropenia |
| D71 | Functional disorders of polymorphonuclear neutrophils |
| D72.0 | Genetic anomalies of leukocytes |
| D72.8 | Other specified disorders of white blood cells |
| D72.9 | Disorder of white blood cells, unspecified |
| D76.1 | Hemophagocytic lymphohistiocytosis |
| D80.0 | Hereditary hypogammaglobulinemia |
| D80.1 | Nonfamilial hypogammaglobulinemia |
| D80.2 | Selective deficiency of immunoglobulin A [IgA] |
| D80.3 | Selective deficiency of immunoglobulin G [IgG] subclasses |
| D80.4 | Selective deficiency of immunoglobulin M [IgM] |
| D80.5 | Immunodeficiency with increased immunoglobulin M [IgM] |
| D80.6 | Antibody deficiency with near-normal immunoglobulins or with hyperimmunoglobulinemia |
| D80.7 | Transient hypogammaglobulinemia of infancy |
| D80.8 | Other immunodeficiencies with predominantly antibody defects |
| D80.9 | Immunodeficiency with predominantly antibody defects, unspecified |
| D81.0 | Severe combined immunodeficiency [SCID] with reticular dysgenesis |
| D81.1 | Severe combined immunodeficiency [SCID] with low T- and B-cell numbers |
| D81.2 | Severe combined immunodeficiency [SCID] with low or normal B-cell numbers |
| D81.3 | Adenosine deaminase [ADA] deficiency |
| D81.4 | Nezelof's syndrome |
| D81.5 | Purine nucleoside phosphorylase [PNP] deficiency |
| D81.6 | Major histocompatibility complex class I deficiency |
| D81.8 | Other combined immunodeficiencies |
| D81.9 | Combined immunodeficiency, unspecified |
| D82.0 | Wiskott-Aldrich syndrome |
| D82.1 | Di George's syndrome |
| D82.2 | Immunodeficiency with short-limbed stature |
| D82.3 | Immunodeficiency following hereditary defective response to Epstein-Barr virus |
| D82.4 | Hyperimmunoglobulin E [IgE] syndrome |
| D82.8 | Immunodeficiency associated with other specified major defects |
| D82.9 | Immunodeficiency associated with major defect, unspecified |
| D83.0 | Common variable immunodeficiency with predominant abnormalities of B-cell numbers and function |
| D83.1 | Common variable immunodeficiency with predominant immunoregulatory T-cell disorders |
| D83.2 | Common variable immunodeficiency with autoantibodies to B- or T-cells |
| D83.8 | Other common variable immunodeficiencies |
| D83.9 | Common variable immunodeficiency, unspecified |
| D84.0 | Lymphocyte function antigen-1 [LFA-1] defect |
| D84.1 | Defects in the complement system |
| D84.8 | Other specified immunodeficiencies |
| D84.9 | Immunodeficiency, unspecified |
| D89.3 | Immune reconstitution syndrome |
| D89.8 | Other specified disorders involving the immune mechanism, not elsewhere classified |
| D89.9 | Disorder involving the immune mechanism, unspecified |

### Allogenic or autologous haematopoietic stem cell transplants

#### HSCT Medcodes

| **Medcode** | **Description** |
| --- | --- |
| 1392 | Bone marrow transplant |
| 15406 | Allograft of bone marrow NEC |
| 18628 | Transfusion of stem cells |
| 21021 | Graft of bone marrow |
| 22726 | Transfusion of bone marrow |
| 28232 | Peripheral blood stem cell graft |
| 30628 | Bone marrow depression |
| 34414 | Autograft of bone marrow |
| 52943 | Bone-marrow transplant rejection |
| 54420 | Second stage peripheral stem cell infusion |
| 63236 | First stage peripheral stem cell infusion |
| 70870 | Graft of bone marrow NOS |
| 72436 | Other specified graft of bone marrow |
| 73013 | Inj.bone marrow - local action |
| 85492 | Allograft of bone marrow from sibling donor |
| 86063 | Autologous peripheral blood stem cell transplant |
| 89920 | Allogeneic peripheral blood stem cell transplant |
| 93362 | Allograft of cord blood to bone marrow |
| 95840 | Allograft of bone marrow from matched unrelated donor |
| 98608 | Allograft of bone marrow from unmatched unrelated donor |
| 100078 | Graft of cord blood to bone marrow |
| 100912 | Allograft of bone marrow from haploidentical donor |
| 103828 | Blood and marrow transplantation |

## Immunosuppressive or immunomodulating therapy

Patients are considered immunosuppressed when an immunosuppressive or immunomodulating therapy record is found until 3 months after each record.

### Chemotherapy/Radiotherapy Medcodes

| **Medcode** | **Description** |
| --- | --- |
| 320 | Radiotherapy NEC |
| 783 | Chemotherapy |
| 1482 | External radiotherapy |
| 4247 | TB chemotherapy |
| 5019 | Cancer chemotherapy |
| 5333 | Prophylactic chemotherapy |
| 5527 | [V]Radiotherapy session |
| 6153 | Iodine 131 radiotherapy |
| 6997 | Radioactive iodine uptake |
| 9706 | Radiotherapy completed |
| 10346 | Seen in radiotherapy clinic |
| 10542 | Radioactive drug therapy |
| 10776 | Radiotherapy - internal |
| 10950 | Seen by radiotherapist |
| 14887 | Oral chemotherapy |
| 15386 | Intravenous chemotherapy |
| 16662 | [V]Convalescence after radiotherapy |
| 16935 | Progress of radiotherapy |
| 18675 | Post-operartive chemotherapy |
| 18715 | Stereotactic radiotherapy |
| 18832 | Date chemotherapy completed |
| 19467 | Pre-operative chemotherapy |
| 20336 | Radiotherapy to lesion of retina |
| 20381 | [V]Maintenance chemotherapy |
| 22472 | External radiotherapy NOS |
| 22490 | Combined pre-operative chemotherapy and radiotherapy |
| 26149 | Chemotherapy started |
| 28061 | I131 radiotherapy |
| 28071 | Continuous infusion of chemotherapy |
| 28712 | Iodine seed radiotherapy |
| 28809 | Combined post-operative chemotherapy and radiotherapy |
| 29285 | Radiotherapy-tumour palliation |
| 29301 | Radiotherapy purpose - NOS |
| 29679 | Radiotherapy - post-op.control |
| 30264 | Subcutaneous chemotherapy |
| 30283 | Intravesical install chemotherapeutic agent for malignancy |
| 30942 | Seen in radiotherapy clinic |
| 31489 | [V]Chemotherapy session for neoplasm |
| 31527 | Purpose of radiotherapy |
| 31804 | Radiotherapy procedure with complication, without blame |
| 34155 | Prophylactic chemotherapy NOS |
| 35597 | Radiotherapy for analgesia |
| 35609 | [V]Convalescence after chemotherapy |
| 36981 | Awaiting radiotherapy |
| 37123 | Intramuscular chemotherapy |
| 38466 | Radiotherapy started |
| 38662 | Introduction of radioactive caesium into organ NOC |
| 40490 | Intrathecal chemotherapy |
| 41044 | Radiotherapy treatment groups |
| 42671 | Other radiotherapy misc. |
| 43261 | Radiotherapy - pre-op. control |
| 44831 | Radiotherapy stopped |
| 45087 | Non-urgent radiotherapy admisn |
| 45099 | Radiotherapy progress NOS |
| 45500 | [V]Other prophylactic chemotherapy |
| 46028 | Date chemotherapy stopped |
| 46824 | Admit radiotherapy emergency |
| 51781 | [X]Other chemotherapy |
| 51787 | Ext.beam + chemotherapy |
| 51959 | Ambulatory chemotherapy |
| 52108 | Convalescence after radiother. |
| 52984 | [X]Need for other prophylactic chemotherapy |
| 53180 | Radioth.: temp. pelvic implant |
| 54919 | Other radiotherapy NOS |
| 55261 | Radiotherapy changed |
| 55828 | Radiotherapy -intra-op.control |
| 55832 | Radioth. for lymphat.irradiat. |
| 55836 | Radiotherapy for inflammation |
| 58036 | Radiotherapy to lacrimal gland |
| 59684 | Radiotherapy procedures |
| 59801 | H/O: chemotherapy |
| 60076 | Radioth.for immunosuppression |
| 60674 | Radiotherapy to lesion of peripheral nerve |
| 61330 | Stereotactic radiosurgery on tissue of brain |
| 62864 | Radio-chemo.: oral route |
| 64143 | Combined radiotherapy NOS |
| 64801 | Intern.radioth-permanent seeds |
| 64997 | Delivery of chemotherapy for neoplasm |
| 65739 | Preload radioth.- nose |
| 68344 | Radiotherapy to lesion of cornea |
| 68423 | Radiotherapy delivery |
| 69387 | Ext.beam-surgery+chemotherapy |
| 69877 | Radioth.: permanent seeds NOS |
| 70246 | Internal metabolic radiotherap |
| 70290 | Radiomimetic chemotherapy |
| 70445 | Other specified radiotherapy procedures |
| 70478 | Radioth.: temporary implant |
| 70549 | Radioth. for haemopo. irradiat |
| 71598 | Radioth.: temp. abdom. implant |
| 71599 | Internal metabolic radioth.NOS |
| 72348 | Fetus/neonate affected-plac./breast transfer chemotherapy |
| 72850 | Extern.beam+intern.radiotherap |
| 72978 | Combined radiotherapy |
| 73171 | Afterload radioth.-fem.genital |
| 73172 | Radio-chemotherapy NOS |
| 73692 | Radioth.:temporary implant NOS |
| 74898 | Preparing chemotherapy |
| 86329 | Oral delivery of radiotherapy for thyroid ablation |
| 88596 | Plaque radiotherapy to lesion of retina |
| 88889 | Radioactive seed implantation NOC |
| 89452 | Procurement drugs for chemotherapy for neoplasm in bands 1-5 |
| 90743 | Delivery of oral chemotherapy for neoplasm |
| 91102 | Radiotherapy procedures NOS |
| 91433 | Radioactive seed implantation into prostate |
| 91694 | Delivery of a fraction of external beam radiotherapy NEC |
| 91891 | Radiotherapy delivery NOS |
| 91918 | External beam radiotherapy to lesion of retina |
| 92999 | Radioth.: infuse organ cavity |
| 93607 | Give radiosensitising drug |
| 93669 | Radiotherapy preparation |
| 94305 | Short dis.+contact radiotherap |
| 94431 | Delivery of oral chemotherapy for neoplasm NOS |
| 94478 | Radioth:temp.head/neck implant |
| 94479 | Ext.beam-surg.+post-op.radioth |
| 94617 | Afterload radioth.- upper GIT |
| 95009 | Delivery of a fraction of intracavitary radiotherapy |
| 95098 | Radioth.: seeds into cavity |
| 95126 | Other specified radiotherapy delivery |
| 95424 | Delivery of chemotherapy for neoplasm NOS |
| 95693 | Other specified radiotherapy volume definition |
| 96310 | Other specified delivery of chemotherapy for neoplasm |
| 97106 | Radioth.: infiltrate tissue |
| 97154 | X-ray radiotherapy control |
| 98826 | Preload radioth-female genital |
| 98882 | Radio-chemo.: I-V route |
| 98902 | Superficial or orthovoltage treatment for radiotherapy |
| 99469 | Image: field control:radiother |
| 100382 | Radioth.: infuse - skull/brain |
| 100724 | Intern. unsealed radioth. NOS |
| 100725 | Radioth:infuse-urinary bladder |
| 100901 | Megavoltage treatment for simple radiotherapy |
| 101391 | Selective internal radiotherapy microspheres lesion of liver |
| 101693 | Cancer chemotherapy management plan |
| 102671 | Preload radioth-urinary system |
| 103372 | Intern.radioth-unsealed source |
| 103525 | Delivery of a fraction of interstitial radiotherapy |
| 104099 | Megavoltage treatment for complex radiotherapy |
| 104142 | Neoadjuvant chemotherapy |
| 105128 | Short dist/contact radioth NOS |
| 105129 | Preload radioth.- resp.organs |
| 105323 | Delivery fraction complex radiotherapy megavoltage machine |
| 105336 | Electrochemotherapy |
| 106334 | Radioth.: infuse - head/neck |
| 107069 | Radiotherapy preparation NOS |
| 107130 | Radio-chem.: into cavity |
| 107175 | Preload radioth.- orbit |
| 107418 | Combined internal radiotherapy |
| 107734 | Deliver simple parenteral chemother neoplas first attendance |
| 108138 | Radiotherapy |
| 108140 | Preparation for external beam radiotherapy NOS |
| 108202 | Delivery fraction radiotherapy superficial orthovoltage mach |
| 108561 | Other chemotherapy drugs |
| 111213 | Other specified other chemotherapy drugs |
| 111244 | Vol def radioth imag intensity-modul radia ther (IMRT) dosim |

### Chemotherapeutic drugs/Biological therapy Prodcodes

| **Prodcode** | **Description** |
| --- | --- |
| 823 | Methotrexate 2.5mg tablets |
| 877 | Methotrexate 10mg tablets |
| 13428 | Maxtrex 2.5mg tablets (Pfizer Ltd) |
| 17035 | Methotrexate 2.5mg/5ml oral suspension |
| 18424 | Methotrexate sodium 2.5mg Tablet |
| 20951 | Methotrexate 2.5mg tablets (AMCo) |
| 21753 | Maxtrex 10mg tablets (Pfizer Ltd) |
| 28041 | Methotrexate 12.5mg/5ml oral suspension |
| 30780 | Methotrexate 2.5mg Tablet (Pharmacia Ltd) |
| 32111 | Methotrexate 2.5mg tablets (Pfizer Ltd) |
| 34929 | Methotrexate 10mg tablets (Pfizer Ltd) |
| 35752 | Methotrexate 7.5mg/5ml oral suspension |
| 36800 | Methotrexate 10mg/5ml oral solution |
| 36849 | Methotrexate 10mg/5ml oral suspension |
| 41104 | Methotrexate 2.5mg tablets (Wockhardt UK Ltd) |
| 41585 | Methotrexate sodium 2.5mg Tablet (Wyeth Pharmaceuticals) |
| 49951 | Methotrexate 2.5mg tablets (Sandoz Ltd) |
| 51120 | Methotrexate 2.5mg tablets (Alliance Healthcare (Distribution) Ltd) |
| 52606 | Methotrexate 2.5mg tablets (Sigma Pharmaceuticals Plc) |
| 53385 | Methotrexate 2.5mg tablets (Waymade Healthcare Plc) |
| 56037 | Methotrexate 2.5mg tablets (A A H Pharmaceuticals Ltd) |
| 57174 | Methotrexate 10mg tablets (Waymade Healthcare Plc) |
| 57441 | Methotrexate 10mg tablets (A A H Pharmaceuticals Ltd) |
| 58303 | Methotrexate 2.5mg tablets (Orion Pharma (UK) Ltd) |
| 58885 | Methotrexate 10mg tablets (Sigma Pharmaceuticals Plc) |
| 59538 | Methotrexate 10mg tablets (Teva UK Ltd) |
| 59685 | Methotrexate 2.5mg tablets (Teva UK Ltd) |
| 59723 | Methotrexate 7.5mg/5ml oral solution |
| 60979 | Methotrexate 2.5mg tablets (Morningside Healthcare Ltd) |
| 61085 | Methotrexate 2.5mg tablets (Waymade Healthcare Plc) |
| 62833 | Methotrexate 2.5mg tablets (DE Pharmaceuticals) |
| 65584 | Methotrexate 2mg/ml oral solution sugar free |
| 68955 | Methotrexate 10mg tablets (Sandoz Ltd) |
| 70977 | Methotrexate 12mg/5ml oral suspension |
| 451 | Azathioprine 25mg tablets |
| 571 | Azathioprine 50mg tablets |
| 671 | Imuran 25mg Tablet (Wellcome Medical Division) |
| 770 | Azathioprine capsules |
| 1899 | Imuran 50mg Tablet (Wellcome Medical Division) |
| 12339 | Azamune 50mg Tablet (Penn Pharmaceuticals Ltd) |
| 13320 | Azathioprine 10mg tablets |
| 19072 | Oprisine 50mg Tablet (Opus Pharmaceuticals Ltd) |
| 21899 | Immunoprin 50mg tablets (Ashbourne Pharmaceuticals Ltd) |
| 22982 | Azathioprine 50mg/5ml oral solution |
| 26261 | Berkaprine 50mg Tablet (Rorer Pharmaceuticals Ltd) |
| 29340 | Azathioprine 50mg tablets (IVAX Pharmaceuticals UK Ltd) |
| 30495 | Imuran 10mg Tablet (Wellcome Medical Division) |
| 31215 | Azathioprine 50mg tablets (Kent Pharmaceuticals Ltd) |
| 32101 | Azathioprine 25mg tablets (A A H Pharmaceuticals Ltd) |
| 34451 | Azathioprine 50mg tablets (Mylan) |
| 34687 | Azathioprine 50mg tablets (A A H Pharmaceuticals Ltd) |
| 34816 | Azathioprine 25mg tablets (Mylan) |
| 35518 | Azathioprine 50mg/5ml oral suspension |
| 36792 | Azathioprine 250mg/5ml oral solution |
| 39115 | Azathioprine 10mg capsules |
| 41620 | Azathioprine 50mg tablets (Teva UK Ltd) |
| 41670 | Azathioprine 50mg Tablet (C P Pharmaceuticals Ltd) |
| 42988 | Imuran 50mg tablets (Aspen Pharma Trading Ltd) |
| 43077 | Imuran 25mg tablets (Aspen Pharma Trading Ltd) |
| 43562 | Azathioprine 50mg tablets (Actavis UK Ltd) |
| 51181 | Azathioprine 60mg/5ml oral solution |
| 52921 | Azathioprine 125mg/5ml oral suspension |
| 53797 | Azathioprine 50mg tablets (Arrow Generics Ltd) |
| 53869 | Azathioprine 20mg/5ml oral solution |
| 53956 | Azathioprine 50mg tablets (Almus Pharmaceuticals Ltd) |
| 54982 | Azathioprine 20mg/5ml oral suspension |
| 55773 | Azathioprine 10mg/5ml oral suspension |
| 58654 | Azathioprine 50mg tablets (Sandoz Ltd) |
| 59006 | Azathioprine 25mg tablets (Kent Pharmaceuticals Ltd) |
| 61160 | Azathioprine 50mg tablets (Tillomed Laboratories Ltd) |
| 63121 | Azathioprine 25mg tablets (Alliance Healthcare (Distribution) Ltd) |
| 65339 | Azapress 50mg tablets (Ennogen Pharma Ltd) |
| 66003 | Azathioprine 25mg tablets (Mawdsley-Brooks & Company Ltd) |
| 67421 | Azathioprine 100mg/5ml oral suspension |
| 68643 | Azathioprine 175mg/5ml oral suspension |
| 68977 | Azathioprine 25mg tablets (Sigma Pharmaceuticals Plc) |
| 3450 | Mercaptopurine 50mg tablets |
| 19982 | Mercaptopurine 10mg capsules |
| 29675 | Puri-Nethol 50mg tablets (Aspen Pharma Trading Ltd) |
| 32972 | Mercaptopurine 10mg tablets |
| 47369 | Mercaptopurine Oral solution |
| 52333 | Mercaptopurine 75mg/5ml oral suspension |
| 55772 | Mercaptopurine 25mg/5ml oral suspension |
| 56753 | Mercaptopurine 25mg tablets |
| 57239 | Mercaptopurine 20mg/ml oral suspension |
| 61545 | Mercaptopurine 50mg tablets (Aspen Pharma Trading Ltd) |
| 65629 | Mercaptopurine 30mg capsules |
| 66583 | Mercaptopurine 75mg tablets |
| 67207 | Mercaptopurine 50mg tablets (Alliance Healthcare (Distribution) Ltd) |
| 71002 | Mercaptopurine 50mg tablets (Waymade Healthcare Plc) |
| 6882 | Adalimumab 40mg injection |
| 23850 | Humira 40mg Injection (Abbott Laboratories Ltd) |
| 48660 | Adalimumab 40mg/0.8ml solution for injection pre-filled disposable devices |
| 50121 | Humira 40mg/0.8ml solution for injection pre-filled pen (AbbVie Ltd) |
| 50996 | Adalimumab 40mg/0.8ml solution for injection pre-filled syringes |
| 52833 | Humira 40mg/0.8ml solution for injection pre-filled syringes (AbbVie Ltd) |
| 56593 | Humira 40mg/0.8ml solution for injection vials (AbbVie Ltd) |
| 56972 | Adalimumab 40mg/0.8ml solution for injection vials |
| 67976 | Adalimumab 40mg/0.4ml solution for injection pre-filled disposable devices |
| 68836 | Adalimumab 40mg/0.4ml solution for injection pre-filled syringes |
| 69527 | Humira 40mg/0.4ml solution for injection pre-filled pen (AbbVie Ltd) |
| 14886 | Enbrel 25mg powder and solvent for solution for injection vials (Pfizer Ltd) |
| 15921 | Etanercept 25mg powder and solvent for solution for injection vials |
| 19257 | Enbrel 50mg powder and solvent for solution for injection vials (Wyeth Pharmaceuticals) |
| 26387 | Etanercept 50mg powder and solvent for solution for injection vials |
| 35126 | Etanercept 50mg injection solution |
| 35419 | Enbrel 25mg/0.5ml solution for injection pre-filled syringes (Pfizer Ltd) |
| 36008 | Etanercept 25mg/0.5ml solution for injection pre-filled syringes |
| 36556 | Enbrel 50mg Solution for injection (Pfizer Consumer Healthcare Ltd) |
| 41058 | Enbrel Paediatric 25mg powder and solvent for solution for injection vials (Pfizer Ltd) |
| 47843 | Etanercept 10mg powder and solvent for solution for injection vials |
| 49856 | Enbrel 50mg/1ml solution for injection pre-filled syringes (Pfizer Ltd) |
| 50494 | Etanercept 50mg/1ml solution for injection pre-filled disposable devices |
| 50998 | Etanercept 50mg/1ml solution for injection pre-filled syringes |
| 56580 | Etanercept 10mg powder and solvent for solution for injection vials |
| 61373 | Enbrel 50mg/1ml solution for injection pre-filled MyClic pen (Pfizer Ltd) |
| 66774 | Benepali 50mg/1ml solution for injection pre-filled syringes (Biogen Idec Ltd) |
| 69500 | Benepali 50mg/1ml solution for injection pre-filled pen (Biogen Idec Ltd) |
| 70538 | Benepali 25mg/0.5ml solution for injection pre-filled syringes (Biogen Idec Ltd) |
| 16822 | Infliximab 100mg powder for solution for infusion vials |
| 22392 | Remicade 100mg powder for concentrate for solution for infusion vials (Merck Sharp & Dohme Ltd) |
| 64636 | Inflectra 100mg powder for concentrate for solution for infusion vials (Pfizer Ltd) |
| 67558 | Remsima 100mg powder for concentrate for solution for infusion vials (Napp Pharmaceuticals Ltd) |
| 31598 | MabCampath 30mg/3ml concentrate for solution for infusion ampoules (Schering Health Care Ltd) |
| 42273 | Alemtuzumab 30mg/1ml solution for infusion vials |
| 55815 | MabCampath 30mg/1ml concentrate for solution for infusion vials (Genzyme Therapeutics Ltd) |
| 69589 | Alemtuzumab 12mg/1.2ml solution for infusion vials |
| 38254 | Tysabri 300mg/15ml concentrate for solution for infusion vials (Biogen Idec Ltd) |
| 41191 | Natalizumab 300mg/15ml solution for infusion vials |
| 40626 | Lenalidomide 25mg capsules |
| 41139 | Revlimid 25mg capsules (Celgene Ltd) |
| 42056 | Lenalidomide 5mg capsules |
| 44529 | Lenalidomide 10mg capsules |
| 46205 | Lenalidomide 15mg capsules |
| 56818 | Revlimid 15mg capsules (Celgene Ltd) |
| 58227 | Revlimid 10mg capsules (Celgene Ltd) |
| 60630 | Lenalidomide 2.5mg capsules |
| 67578 | Revlimid 2.5mg capsules (Celgene Ltd) |
| 69801 | Lenalidomide 7.5mg capsules |
| 43703 | Cimzia 200mg/1ml solution for injection pre-filled syringes (UCB Pharma Ltd) |
| 44100 | Certolizumab pegol 200mg/1ml solution for injection pre-filled syringes |
| 68740 | Certolizumab pegol 200mg/1ml solution for injection pre-filled disposable devices |
| 69393 | Cimzia 200mg/1ml solution for injection pre-filled pen (UCB Pharma Ltd) |
| 46370 | Golimumab 50mg/0.5ml solution for injection pre-filled disposable devices |
| 47398 | Golimumab 50mg/0.5ml solution for injection pre-filled syringes |
| 47740 | Simponi 50mg/0.5ml solution for injection pre-filled disposable devices (Merck Sharp & Dohme Ltd) |
| 60284 | Golimumab 100mg/1ml solution for injection pre-filled disposable devices |
| 62618 | Simponi 100mg/1ml solution for injection pre-filled pen (Merck Sharp & Dohme Ltd) |
| 68360 | Simponi 50mg/0.5ml solution for injection pre-filled syringes (Merck Sharp & Dohme Ltd) |
| 6891 | Thalidomide 50mg capsules |
| 50962 | Thalidomide 50mg tablets |
| 55608 | Thalidomide Celgene 50mg capsules (Celgene Ltd) |
| 65643 | Thalidomide 25mg tablets |
| 59048 | Pomalidomide 2mg capsules |
| 71175 | Imnovid 2mg capsules (Celgene Ltd) |
| 60707 | Ofatumumab 100mg/5ml solution for infusion vials |
| 28490 | Rituximab 100mg/10ml solution for infusion vials |
| 36294 | Rituximab 500mg/50ml solution for infusion vials |
| 39111 | Rituximab 10mg/ml concentrated intravenous infusion |
| 47502 | MabThera 100mg/10ml concentrate for solution for infusion vials (Roche Products Ltd) |
| 3873 | Hydroxyurea Capsule |
| 6333 | Hydroxycarbamide 500mg capsules |
| 6884 | Hydrea 500mg capsules (Bristol-Myers Squibb Pharmaceuticals Ltd) |
| 33330 | Hydroxycarbamide 500mg capsules (medac UK) |
| 38319 | Hydroxycarbamide 500mg/5ml oral solution |
| 39548 | Hydroxycarbamide 1g tablets |
| 47127 | Hydroxycarbamide 100mg tablets |
| 50565 | Hydroxycarbamide 300mg capsules |
| 59007 | Hydroxycarbamide 500mg capsules (A A H Pharmaceuticals Ltd) |
| 62157 | Hydroxycarbamide 500mg/5ml oral suspension |
| 65463 | Siklos 1000mg tablets (Nordic Pharma Ltd) |
| 3874 | Busulfan 2mg tablets |
| 22204 | Busulfan 500micrograms tablets |
| 26301 | Myleran 2mg Tablet (Wellcome Medical Division) |
| 32412 | Myleran 500microgram Tablet (Wellcome Medical Division) |
| 3984 | Cyclophosphamide 10mg tablets |
| 3985 | Cyclophosphamide 50mg tablets |
| 10729 | Endoxana 50mg tablets (Baxter Healthcare Ltd) |
| 16105 | Cyclophosphamide 500mg powder for solution for injection vials |
| 26066 | Cyclophosphamide 200mg powder for solution for injection vials |
| 26322 | Cyclophosphamide 100mg injection |
| 29840 | Cyclophosphamide 1g powder for solution for injection vials |
| 31193 | Endoxana 10mg Tablet (Baxter Healthcare Ltd) |
| 34728 | Cyclophosphamide 50mg Tablet (Pharmacia Ltd) |
| 44273 | Endoxana 200mg powder for solution for injection vials (Baxter Healthcare Ltd) |
| 44309 | Endoxana 1g powder for solution for injection vials (Baxter Healthcare Ltd) |
| 47752 | Cyclophosphamide 25mg tablets |
| 4230 | Mycophenolate mofetil 500mg tablets |
| 4438 | Mycophenolate mofetil 250mg capsules |
| 7077 | Mycophenolate mofetil 500mg powder for solution for infusion vials |
| 16879 | Mycophenolate mofetil 1g/5ml oral suspension sugar free |
| 16919 | CellCept 250mg capsules (Roche Products Ltd) |
| 18804 | CellCept 500mg tablets (Roche Products Ltd) |
| 21732 | CellCept 1g/5ml oral suspension (Roche Products Ltd) |
| 26097 | Mycophenolic acid 360mg gastro-resistant tablets |
| 27289 | Myfortic 360mg gastro-resistant tablets (Novartis Pharmaceuticals UK Ltd) |
| 27290 | Myfortic 180mg gastro-resistant tablets (Novartis Pharmaceuticals UK Ltd) |
| 30581 | CellCept 500mg powder for solution for infusion vials (Roche Products Ltd) |
| 35301 | Mycophenolic acid 180mg gastro-resistant tablets |
| 45043 | Myfenax 500mg tablets (Teva UK Ltd) |
| 45393 | Arzip 500mg tablets (Zentiva) |
| 45489 | Myfenax 250mg capsules (Teva UK Ltd) |
| 47746 | Mycophenolate motefil 500mg tablets (Wockhardt UK Ltd) |
| 47789 | Arzip 250mg capsules (Zentiva) |
| 50669 | Mycophenolate motefil 500mg tablets (Sandoz Ltd) |
| 52488 | CellCept 250mg capsules (Lexon (UK) Ltd) |
| 53255 | Mycophenolate mofetil 250mg capsules (Sigma Pharmaceuticals Plc) |
| 54317 | Mycophenolate mofetil 250mg capsules (Sandoz Ltd) |
| 57272 | Mycophenolate mofetil 125mg/5ml oral suspension |
| 57593 | CellCept 500mg tablets (Waymade Healthcare Plc) |
| 58530 | Mycophenolate mofetil 500mg tablets (Sigma Pharmaceuticals Plc) |
| 60231 | Mycophenolate mofetil 250mg capsules (A A H Pharmaceuticals Ltd) |
| 70228 | Mycophenolate mofetil 500mg powder for concentrate for solution for infusion vials (Accord Healthcare Ltd) |
| 71049 | Mycophenolate mofetil 250mg capsules (Cubic Pharmaceuticals Ltd) |
| 4970 | Leflunomide 100mg tablets |
| 4971 | Leflunomide 10mg tablets |
| 6934 | Leflunomide 20mg tablets |
| 16522 | Arava 10mg tablets (Sanofi) |
| 17642 | Arava 20mg tablets (Sanofi) |
| 18460 | Arava 100mg tablets (Sanofi) |
| 48217 | Leflunomide 10mg tablets (medac UK) |
| 62007 | Leflunomide 15mg tablets |
| 62993 | Leflunomide 20mg tablets (Sandoz Ltd) |
| 67739 | Leflunomide 20mg tablets (Teva UK Ltd) |
| 68671 | Leflunomide 20mg tablets (Alliance Healthcare (Distribution) Ltd) |
| 68672 | Leflunomide 10mg tablets (Alliance Healthcare (Distribution) Ltd) |
| 71099 | Leflunomide 10mg tablets (Aspire Pharma Ltd) |
| 5600 | Chlorambucil 2mg tablets |
| 8665 | Chlorambucil 5mg tablets |
| 16838 | Leukeran 2mg Tablet (Wellcome Medical Division) |
| 26315 | Leukeran 5mg tablets (GlaxoSmithKline UK Ltd) |
| 7340 | Capecitabine 150mg tablets |
| 7341 | Capecitabine 500mg tablets |
| 18063 | Xeloda 500mg tablets (Roche Products Ltd) |
| 33127 | Xeloda 150mg tablets (Roche Products Ltd) |
| 65607 | Capecitabine 150mg tablets (A A H Pharmaceuticals Ltd) |
| 8404 | Ccnu 40mg Capsule (Lundbeck Ltd) |
| 12067 | Lomustine 40mg capsules |
| 20721 | CCNU |
| 25848 | Ccnu 10mg Capsule (Lundbeck Ltd) |
| 8756 | Etoposide 50mg capsules |
| 18751 | Etoposide 100mg capsules |
| 29761 | Vepesid 50mg capsules (Bristol-Myers Squibb Pharmaceuticals Ltd) |
| 31115 | Etoposide 20mg/ml Solution for infusion |
| 36263 | Eposin 20mg/ml Concentrate for solution for infusion (medac UK) |
| 37375 | Vepesid 100mg capsules (Bristol-Myers Squibb Pharmaceuticals Ltd) |
| 44387 | Etoposide 100mg powder for solution for injection vials |
| 48177 | Etoposide 100mg/5ml solution for infusion vials |
| 63011 | Etoposide 500mg/25ml solution for infusion vials |
| 10328 | Carboplatin 10mg/ml concentrate solution for infusion |
| 18236 | Carboplatin 50mg/vial injection |
| 24096 | Carboplatin 150mg injection |
| 35855 | Carboplatin 50mg/5ml solution for infusion vials |
| 37542 | Paraplatin 150mg Injection (Bristol-Myers Squibb Pharmaceuticals Ltd) |
| 39450 | Carboplatin 600mg/60ml solution for infusion vials |
| 40781 | Carboplatin 150mg/15ml solution for infusion vials |
| 41963 | Paraplatin 10mg/ml Concentrate for solution for infusion (Bristol-Myers Squibb Pharmaceuticals Ltd) |
| 61065 | Carboplatin 450mg/45ml solution for infusion vials |
| 11003 | Cerubidin 20mg/vial Powder for solution for injection (Rhone-Poulenc Rorer Ltd) |
| 43805 | Daunorubicin 20mg powder for solution for infusion vials |
| 12150 | Melphalan 5mg tablets |
| 16929 | Melphalan 2mg tablets |
| 23270 | Alkeran 5mg tablets (GlaxoSmithKline UK Ltd) |
| 26343 | Alkeran 2mg Tablet (Wellcome Medical Division) |
| 26580 | Melphalan 100mg/vial Injection |
| 37099 | Melphalan 50mg powder and solvent for solution for injection vials |
| 13604 | Estracyt 140mg capsules (Pfizer Ltd) |
| 13735 | Estramustine 140mg capsules |
| 14381 | Paclitaxel 6mg/ml concentrated solution for infusion |
| 16173 | Taxol 6mg/ml Concentrate for solution for infusion (Bristol-Myers Squibb Pharmaceuticals Ltd) |
| 35384 | Taxol 30mg/5ml solution for infusion vials (Bristol-Myers Squibb Pharmaceuticals Ltd) |
| 35854 | Paclitaxel 30mg/5ml solution for infusion vials |
| 39919 | Paclitaxel 100mg powder for suspension for infusion vials |
| 45147 | Paclitaxel 150mg/25ml solution for infusion vials |
| 47754 | Paclitaxel 100mg/16.7ml solution for infusion vials |
| 61366 | Abraxane 100mg powder for suspension for infusion vials (Celgene Ltd) |
| 69037 | Paclitaxel 300mg/50ml solution for infusion vials |
| 15405 | Novantrone 2mg/ml Concentrate for solution for infusion (Wyeth Pharmaceuticals) |
| 33174 | Mitoxantrone 2mg/ml Concentrate for solution for infusion |
| 39366 | Mitoxantrone 10mg/5ml solution for infusion vials |
| 41267 | Mitoxantrone 20mg/10ml solution for infusion vials |
| 17186 | Procarbazine 50mg capsules |
| 28605 | Natulan 50mg Capsule (Cambridge Laboratories Ltd) |
| 4261 | Efudix 5% Cream (Valeant Pharmaceuticals Ltd) |
| 4650 | Fluorouracil 5% cream |
| 18070 | Fluorouracil 250mg capsules |
| 19556 | Fluoro-uracil 250mg Capsule (Cambridge Laboratories Ltd) |
| 20229 | Fluoro-uracil 25mg/ml Injection (Cambridge Laboratories Ltd) |
| 36575 | Fluorouracil 50mg/ml Injection |
| 39388 | Fluorouracil 1g/20ml solution for injection vials |
| 43599 | Efudix 5% cream (Meda Pharmaceuticals Ltd) |
| 46303 | Actikerall 5mg/g / 100mg/g cutaneous solution (Almirall Ltd) |
| 46368 | Fluorouracil 0.5% / Salicylic acid 10% cutaneous solution |
| 51713 | Efudix 5% cream (Sigma Pharmaceuticals Plc) |
| 55091 | Fluorouracil 500mg/10ml solution for injection vials |
| 58240 | Fluorouracil 2.5g/100ml solution for infusion vials |
| 66876 | Efudix 5% cream (Mawdsley-Brooks & Company Ltd) |
| 18238 | Dacarbazine 100mg powder for solution for injection vials |
| 28682 | Dacarbazine 200mg powder for solution for injection vials |
| 32204 | Dtic-dome 100mg/vial Injection (Bayer Plc) |
| 18476 | Fludarabine phosphate 10mg tablets |
| 24681 | Fludarabine phosphate 50mg powder for solution for injection vials |
| 29743 | Fludara 10mg tablets (Sanofi) |
| 19335 | Cytosar 500mg Injection (Pharmacia Ltd) |
| 41266 | Cytarabine 1g/10ml solution for injection vials |
| 47450 | Cytosar 100mg Injection (Pharmacia Ltd) |
| 47522 | Cytarabine 100mg/5ml solution for injection vials |
| 66380 | DepoCyte 50mg/5ml suspension for injection vials (Napp Pharmaceuticals Ltd) |
| 20094 | Tioguanine 40mg tablets |
| 26502 | Lanvis 40mg tablets (Aspen Pharma Trading Ltd) |
| 21249 | Temozolomide 100mg capsules |
| 21250 | Temozolomide 250mg capsules |
| 27922 | Temozolomide 5mg capsules |
| 29700 | Temodal 250mg capsules (Merck Sharp & Dohme Ltd) |
| 32490 | Temozolomide 20mg capsules |
| 33803 | Temodal 5mg capsules (Merck Sharp & Dohme Ltd) |
| 35226 | Temodal 20mg capsules (Merck Sharp & Dohme Ltd) |
| 42372 | Temozolomide 140mg capsules |
| 55077 | Temodal 100mg capsules (Merck Sharp & Dohme Ltd) |
| 21286 | Imatinib 100mg capsules |
| 21295 | Glivec 100mg capsules (Novartis Pharmaceuticals UK Ltd) |
| 21318 | Imatinib 400mg tablets |
| 28800 | Glivec 400mg tablets (Novartis Pharmaceuticals UK Ltd) |
| 29229 | Imatinib 100mg tablets |
| 33823 | Glivec 100mg tablets (Novartis Pharmaceuticals UK Ltd) |
| 12066 | Razoxin 125mg Tablet (Cambridge Laboratories Ltd) |
| 22640 | Razoxane 125mg tablets |
| 23832 | Eloxatin 100mg powder for solution for infusion vials (Sanofi) |
| 27293 | Oxaliplatin 50mg powder for solution for infusion vials |
| 36714 | Oxaliplatin 100mg powder for solution for infusion vials |
| 39553 | Oxaliplatin 50mg/10ml solution for infusion vials |
| 39895 | Oxaliplatin 100mg/20ml solution for infusion vials |
| 50277 | Oxaliplatin 100mg/20ml concentrate for solution for infusion vials (A A H Pharmaceuticals Ltd) |
| 58942 | Oxaliplatin 200mg/40ml concentrate for solution for infusion vials (Accord Healthcare Ltd) |
| 23849 | Taxotere 40mg/ml Concentrate for intravenous infusion (Aventis Pharma) |
| 33560 | Docetaxel 40mg/ml conc IV infusion |
| 36552 | Taxotere 20mg/0.5ml solution for infusion vials and diluent (Sanofi) |
| 36831 | Docetaxel 20mg/0.5ml solution for infusion vials and diluent |
| 38999 | Docetaxel 80mg/2ml solution for infusion vials and diluent |
| 44087 | Taxotere 20mg/1ml concentrate for solution for infusion vials (Sanofi) |
| 44425 | Docetaxel 20mg/1ml solution for infusion vials |
| 47571 | Taxotere 160mg/8ml concentrate for solution for infusion vials (Sanofi) |
| 47755 | Docetaxel 160mg/16ml solution for infusion vials |
| 55155 | Docetaxel 20mg/2ml solution for infusion vials |
| 64944 | Docetaxel 80mg/4ml solution for infusion vials |
| 66480 | Docetaxel 80mg/8ml solution for infusion vials |
| 23871 | Treosulfan 250mg Capsule (Farillon Ltd) |
| 24448 | Treosulfan 250mg capsules |
| 28726 | TREOSULFAN LEO |
| 24997 | Caelyx 2mg/ml Concentrate for solution for infusion (Schering-Plough Ltd) |
| 26680 | Adriamycin 10mg/vial Injection (Pharmacia Ltd) |
| 26947 | Doxorubicin 2mg/ml injection |
| 27469 | Adriamycin 50mg/vial Injection (Pharmacia Ltd) |
| 30836 | Doxorubicin 10mg powder for solution for injection vials |
| 33878 | Myocet 50mg powder and solvent for suspension for infusion vials (Teva UK Ltd) |
| 37784 | Caelyx 20mg/10ml concentrate for solution for infusion vials (Janssen-Cilag Ltd) |
| 37942 | Doxorubicin (liposomal) 50mg powder and solvent for suspension for infusion vials |
| 39307 | Doxorubicin 10mg/5ml solution for injection vials |
| 40250 | Caelyx 50mg/25ml concentrate for solution for infusion vials (Janssen-Cilag Ltd) |
| 45026 | Doxorubicin 200mg/100ml solution for infusion vials |
| 55271 | Doxorubicin encapsulated in liposomes 2mg/ml concentrate solution for infusion |
| 56410 | Caelyx 50mg/25ml concentrate for solution for infusion vials (Janssen-Cilag Ltd) |
| 71084 | Caelyx 20mg/10ml concentrate for solution for infusion vials (Janssen-Cilag Ltd) |
| 25740 | Avastin 100mg/4ml solution for infusion vials (Roche Products Ltd) |
| 38145 | Bevacizumab 100mg/4ml solution for infusion vials |
| 26119 | Chlormethine 10mg/ml Injection (Sovereign Medical Ltd) |
| 28709 | Chlormethine 10mg solution for injection vials |
| 26332 | THIOTEPA 15 MG INJ |
| 33385 | Thiotepa 15mg powder for solution for injection vials |
| 27071 | Dactinomycin 500microgram powder for solution for injection vials |
| 47695 | Actinomycin D 500micrograms/vial sterile powder |
| 58496 | Cosmegen Lyovac 500microgram powder for solution for injection vials (Orphan Europe (UK) Ltd) |
| 28324 | Cisplatin 25mg/vial powder |
| 32824 | Cisplatin 1mg/ml concentrate for solution for infusion |
| 38185 | Cisplatin 50mg powder for solution for infusion vials |
| 38453 | Cisplatin 10mg/10ml solution for infusion vials |
| 44388 | Cisplatin 50mg/50ml solution for infusion vials |
| 48068 | Cisplatin 100mg/100ml solution for infusion vials |
| 68944 | Cisplatin 100mg/100ml concentrate for solution for infusion vials (Accord Healthcare Ltd) |
| 28325 | Epirubicin 50mg powder for solution for injection vials |
| 28889 | Pharmorubicin 2mg/ml Solution for injection (Pharmacia Ltd) |
| 29652 | Epirubicin HCL 2mg/ml injection |
| 31539 | Epirubicin 20mg powder for solution for injection vials |
| 39387 | Epirubicin 100mg/50ml solution for infusion vials |
| 40251 | Epirubicin 200mg/100ml solution for infusion vials |
| 40816 | Epirubicin 50mg/25ml solution for injection vials |
| 43639 | Pharmorubicin Rapid Dissolution 50mg powder for solution for injection vials (Pfizer Ltd) |
| 30014 | Mithracin 2.5mg/vial Injection (Pfizer Ltd) |
| 31494 | Mithramycin 2.5mg/vial Injection |
| 30757 | MITOBRONITOL (NAMED PATIENT ONLY) 125 MG TAB |
| 37395 | MYELOBROMOL (NAMED PATIENT ONLY) 125 MG TAB |
| 37396 | Myelobromol 125mg Tablet (Durbin Plc) |
| 31223 | Vinblastine 10mg powder for solution for injection vials |
| 32604 | Vinblastine 10mg/10ml solution for injection vials |
| 31339 | Idarubicin 10mg capsules |
| 31984 | Idarubicin 5mg capsules |
| 32614 | Simulect 20mg powder and solvent for solution for injection vials (Novartis Pharmaceuticals UK Ltd) |
| 37915 | Basiliximab 10mg powder and solvent for solution for injection vials |
| 40632 | Basiliximab 20mg powder and solvent for solution for injection vials |
| 61351 | Simulect 10mg powder and solvent for solution for injection vials (Novartis Pharmaceuticals UK Ltd) |
| 32774 | Vinorelbine 20mg capsules |
| 33171 | Vinorelbine 10mg/ml injection solution |
| 42684 | Vinorelbine 10mg/1ml solution for infusion vials |
| 46838 | Vinorelbine 80mg capsules |
| 33227 | Hycamtin 4mg powder for concentrate for solution for infusion vials (Novartis Pharmaceuticals UK Ltd) |
| 41960 | Topotecan 1mg powder for solution for infusion vials |
| 47396 | Topotecan 1mg capsules |
| 33418 | Gemcitabine 200mg powder for solution for infusion vials |
| 40780 | Gemcitabine 1g powder for solution for infusion vials |
| 33519 | Uftoral capsules (Merck Serono Ltd) |
| 33520 | Tegafur 100mg / Uracil 224mg capsules |
| 35826 | Mitotane 500mg tablets |
| 44478 | Lysodren 500mg tablets (HRA Pharma UK Ltd) |
| 36062 | Dasatinib 50mg tablets |
| 36957 | Dasatinib 70mg tablets |
| 37238 | Dasatinib 20mg tablets |
| 42390 | Dasatinib 100mg tablets |
| 65280 | Dasatinib 80mg tablets |
| 37272 | Pemetrexed 500mg powder for solution for infusion vials |
| 70261 | Pemetrexed 100mg/4ml solution for infusion vials |
| 38081 | Erwinase 10,000unit powder for solution for injection vials (Jazz Pharmaceuticals UK) |
| 47194 | Asparaginase 5000 unit/vial Powder for solution for injection (Imported) |
| 64633 | Crisantaspase 10,000unit powder for solution for injection vials |
| 68388 | Asparaginase 10,000unit powder for solution for infusion vials |
| 38317 | Nilotinib 200mg capsules |
| 45820 | Nilotinib 150mg capsules |
| 40453 | Torisel 30mg/1.2ml concentrate for solution for infusion vials and diluent (Pfizer Ltd) |
| 40454 | Cladribine 10mg/5ml solution for injection vials |
| 67179 | Cladribine 10mg/10ml solution for infusion vials |
| 40732 | Velcade 3.5mg powder for solution for injection vials (Janssen-Cilag Ltd) |
| 44740 | Bortezomib 3.5mg powder for solution for injection vials |
| 40983 | Irinotecan 40mg/2ml solution for infusion vials |
| 59339 | Irinotecan 40mg/2ml concentrate for solution for infusion vials (Pfizer Ltd) |
| 68267 | Irinotecan 100mg/5ml solution for infusion vials |
| 41281 | Carmustine 7.7mg implant |
| 41502 | Tocilizumab 80mg/4ml solution for infusion vials |
| 46348 | Tocilizumab 200mg/10ml solution for infusion vials |
| 56962 | RoActemra 200mg/10ml concentrate for solution for infusion vials (Roche Products Ltd) |
| 62873 | Tocilizumab 162mg/0.9ml solution for injection pre-filled syringes |
| 62957 | Tocilizumab 400mg/20ml solution for infusion vials |
| 67985 | RoActemra 162mg/0.9ml solution for injection pre-filled syringes (Roche Products Ltd) |
| 42696 | Thymoglobuline 25mg powder and solvent for solution for infusion vials (Sanofi) |
| 42817 | Daclizumab 25mg/5ml solution for infusion vials |
| 70409 | Daclizumab 150mg/1ml solution for injection pre-filled disposable devices |
| 43168 | Ifosfamide 2g powder for solution for injection vials |
| 43781 | Afinitor 10mg tablets (Novartis Pharmaceuticals UK Ltd) |
| 46070 | Everolimus 5mg tablets |
| 55683 | Everolimus 10mg tablets |
| 63638 | Afinitor 2.5mg tablets (Novartis Pharmaceuticals UK Ltd) |
| 65378 | Everolimus 500microgram tablets |
| 70816 | Everolimus 250microgram tablets |
| 43888 | Pentostatin 10mg powder for solution for injection vials |
| 44222 | Raltitrexed 2mg powder for solution for infusion vials |
| 45647 | Canakinumab 150mg powder for solution for injection vials |
| 25851 | Bleo-Kyowa 15,000unit powder for solution for injection vials (Kyowa Kirin Ltd) |
| 32261 | Bleomycin 15,000unit powder for solution for injection vials |
| 18914 | Vincristine sulphate 1mg injection |
| 25589 | Vincristine sulphate 1mg/ml injection |
| 26316 | Vincristine sulphate 2mg injection |
| 27224 | Oncovin 2mg Injection (Eli Lilly and Company Ltd) |
| 40649 | Vincristine 1mg/1ml solution for injection vials |
| 40659 | Vincristine sulphate 5mg injection |
| 55823 | Vincristine 5mg/5ml solution for injection vials |
| 2169 | Calcium folinate 15mg tablets |
| 22811 | Calcium folinate 15mg/vial injection powder |
| 23949 | Calcium leucovorin 15mg Tablet (Wyeth Pharmaceuticals) |
| 26006 | Calcium folinate 10mg/ml injection solution |
| 26320 | Calcium leucovorin 15mg/vial Injection (Wyeth Pharmaceuticals) |
| 26702 | Folinic acid 350mg/35ml Injection |
| 28208 | Refolinon 15mg tablets (Pfizer Ltd) |
| 31524 | Calcium leucovorin 15mg tablets |
| 33261 | Refolinon 3mg/ml Injection (Pharmacia Ltd) |
| 34599 | Calcium folinate 15mg Tablet (Bristol-Myers Squibb Pharmaceuticals Ltd) |
| 38371 | Calcium folinate 15mg/2ml solution for injection ampoules |
| 39201 | Calcium folinate 3mg/1ml solution for injection ampoules |
| 39468 | Calcium folinate 30mg/10ml solution for injection ampoules |
| 39574 | Calcium leucovorin 15mg/2ml injection solution |
| 53763 | Lederfolin 350mg/vial Injection (Wyeth Pharmaceuticals) |
| 55282 | Calcium leucovorin 3mg/ml Injection (Wyeth Pharmaceuticals) |
| 55283 | Calcium folinate 3mg/ml injection solution |
| 61432 | Calcium folinate 15mg/5ml oral solution |
| 113 | Mitomycin 10mg powder for solution for injection vials |
| 19287 | Mitomycin 2mg powder for solution for injection vials |
| 30051 | Mitomycin-C Kyowa 2mg powder for solution for injection vials (Kyowa Kirin Ltd) |
| 32593 | Mitomycin 40mg powder for solution for injection vials |
| 44594 | Mitomycin-C Kyowa 10mg powder for solution for injection vials (Kyowa Kirin Ltd) |
| 48007 | Mitomycin-C Kyowa 40mg powder for solution for injection vials (Kyowa Kirin Ltd) |
| 60004 | Mitomycin 0.04% eye drops preservative free |
| 61458 | Mitomycin 20mg powder for solution for injection vials |
| 59842 | Azacitidine 100mg powder for suspension for injection vials |
| 27292 | Herceptin 150mg powder for solution for infusion vials (Roche Products Ltd) |
| 31076 | Trastuzumab 150mg powder for solution for infusion vials |
| 58819 | Herceptin 600mg/5ml solution for injection vials (Roche Products Ltd) |
| 32712 | Erbitux 100mg/50ml solution for infusion vials (Merck Serono Ltd) |
| 33205 | Cetuximab 100mg/50ml solution for infusion vials |
| 38158 | Cetuximab 100mg/20ml solution for infusion vials |
| 59276 | Erbitux 100mg/20ml solution for infusion vials (Merck Serono Ltd) |
| 66436 | Cetuximab 500mg/100ml solution for infusion vials |
| 809 | Prostap 3 Depot 11.25mg powder and solvent for suspension for injection vials (Takeda UK Ltd) |
| 3900 | Prostap SR 3.75mg powder and solvent for suspension for injection vials (Takeda UK Ltd) |
| 6691 | Leuprorelin 3.75mg Powder for solution for injection |
| 11382 | Leuprorelin 11.25mg Powder for solution for injection |
| 46213 | Prostap 3 DCS 11.25mg powder and solvent for suspension for injection pre-filled syringes (Takeda UK Ltd) |
| 46352 | Prostap SR DCS 3.75mg powder and solvent for suspension for injection pre-filled syringes (Takeda UK Ltd) |
| 48891 | Leuprorelin 3.75mg powder and solvent for suspension for injection pre-filled syringes |
| 48892 | Leuprorelin 11.25mg powder and solvent for suspension for injection pre-filled syringes |
| 51174 | Leuprorelin 3.75mg powder and solvent for suspension for injection vials |
| 52892 | Leuprorelin 11.25mg powder and solvent for suspension for injection vials |
| 55587 | Prostap 3 DCS 11.25mg powder and solvent for suspension for injection pre-filled syringes (DE Pharmaceuticals) |
| 66207 | Lutrate 1 month Depot 3.75mg powder and solvent for suspension for injection vials (AMCo) |
| 66514 | Lutrate 3 month Depot 22.5mg powder and solvent for suspension for injection vials (AMCo) |
| 67282 | Prostap SR DCS 3.75mg powder and solvent for suspension for injection pre-filled syringes (DE Pharmaceuticals) |
| 70865 | Leuprorelin 22.5mg powder and solvent for suspension for injection vials |
| 47139 | Zytiga 250mg tablets (Janssen-Cilag Ltd) |
| 47462 | Abiraterone 250mg tablets |
| 71531 | Abiraterone 500mg tablets |
| 45122 | Panitumumab 100mg/5ml solution for infusion vials |
| 60444 | Cabazitaxel 60mg/1.5ml solution for infusion vials |
| 47767 | Bendamustine 25mg powder for solution for infusion vials |
| 57939 | Bendamustine 100mg powder for solution for infusion vials |
| 44004 | Denosumab 60mg/1ml solution for injection pre-filled syringes |
| 44084 | Prolia 60mg/1ml solution for injection pre-filled syringes (Amgen Ltd) |
| 54058 | Denosumab 120mg/1.7ml solution for injection vials |
| 58018 | Xgeva 120mg/1.7ml solution for injection vials (Amgen Ltd) |
| 56041 | Ipilimumab 50mg/10ml solution for infusion vials |
| 58862 | Ipilimumab 200mg/40ml solution for infusion vials |
| 61908 | Pertuzumab 420mg/14ml solution for infusion vials |
| 61854 | Afatinib 20mg tablets |
| 60484 | Eylea 2mg/50microlitres solution for injection vials (Bayer Plc) |
| 65735 | Aflibercept 2mg/50microlitres solution for injection vials |
| 58025 | Axitinib 1mg tablets |
| 62572 | Axitinib 7mg tablets |
| 68132 | Axitinib 5mg tablets |
| 58758 | Crizotinib 200mg capsules |
| 25262 | Tarceva 150mg tablets (Roche Products Ltd) |
| 33827 | Erlotinib 150mg tablets |
| 37441 | Tarceva 25mg tablets (Roche Products Ltd) |
| 40094 | Erlotinib 100mg tablets |
| 45992 | Erlotinib 25mg tablets |
| 47556 | Tarceva 100mg tablets (Roche Products Ltd) |
| 41077 | Gefitinib 250mg tablets |
| 46563 | Iressa 250mg tablets (AstraZeneca UK Ltd) |
| 46837 | Lapatinib 250mg tablets |
| 46823 | Pazopanib 400mg tablets |
| 47552 | Pazopanib 200mg tablets |
| 56652 | Votrient 200mg tablets (Novartis Pharmaceuticals UK Ltd) |
| 59434 | Ruxolitinib 15mg tablets |
| 60008 | Ruxolitinib 20mg tablets |
| 64275 | Ruxolitinib 5mg tablets |
| 67636 | Jakavi 5mg tablets (Novartis Pharmaceuticals UK Ltd) |
| 69014 | Ruxolitinib 10mg tablets |
| 35685 | Sorafenib 200mg tablets |
| 42362 | Nexavar 200mg tablets (Bayer Plc) |
| 38871 | Sunitinib 12.5mg capsules |
| 39795 | Sutent 12.5mg capsules (Pfizer Ltd) |
| 40580 | Sunitinib 50mg capsules |
| 42993 | Sutent 25mg capsules (Pfizer Ltd) |
| 44063 | Sunitinib 25mg capsules |
| 45450 | Sutent 50mg capsules (Pfizer Ltd) |
| 57172 | Vandetanib 100mg tablets |
| 39794 | Alitretinoin 10mg capsules |
| 41054 | Alitretinoin 30mg capsules |
| 42621 | Toctino 30mg capsules (Stiefel Laboratories (UK) Ltd) |
| 43702 | Toctino 10mg capsules (Stiefel Laboratories (UK) Ltd) |
| 36769 | Bexarotene 75mg capsules |
| 40379 | Targretin 75mg capsules (Eisai Ltd) |
| 26050 | Anagrelide 500microgram capsules |
| 30793 | Xagrid 500microgram capsules (Shire Pharmaceuticals Ltd) |
| 58932 | Vemurafenib 240mg tablets |
| 3986 | Uromitexan 100mg/ml Injection (Baxter Healthcare Ltd) |
| 8926 | Mesna 100mg/ml Injection |
| 20841 | Mesna 400mg tablets |
| 30607 | Uromitexan 400mg tablets (Baxter Healthcare Ltd) |
| 39017 | Mesna 600mg tablets |
| 44843 | Mesna 1g/10ml solution for injection ampoules |
| 60790 | Mesna 400mg/4ml solution for injection ampoules |
| 972 | Neoral 25mg capsules (Novartis Pharmaceuticals UK Ltd) |
| 973 | Neoral 100mg capsules (Novartis Pharmaceuticals UK Ltd) |
| 1626 | Ciclosporin 100mg/ml oral solution sugar free |
| 1905 | Neoral 100mg/ml oral solution (Novartis Pharmaceuticals UK Ltd) |
| 2837 | Ciclosporin 50mg capsules |
| 2838 | Ciclosporin 25mg capsules |
| 3896 | Ciclosporin 100mg capsules |
| 3920 | Sandimmun 25mg capsules (Novartis Pharmaceuticals UK Ltd) |
| 4231 | Neoral 50mg capsules (Novartis Pharmaceuticals UK Ltd) |
| 13494 | Sandimmun 100mg/ml oral solution (Novartis Pharmaceuticals UK Ltd) |
| 13556 | Sandimmun 100mg capsules (Novartis Pharmaceuticals UK Ltd) |
| 15596 | Sandimmun 50mg capsules (Novartis Pharmaceuticals UK Ltd) |
| 16035 | Ciclosporin 10mg capsules |
| 16137 | Neoral 10mg capsules (Novartis Pharmaceuticals UK Ltd) |
| 42448 | Deximune 50mg capsules (Dexcel-Pharma Ltd) |
| 42449 | Deximune 100mg capsules (Dexcel-Pharma Ltd) |
| 42637 | Deximune 25mg capsules (Dexcel-Pharma Ltd) |
| 46395 | Capimune 100mg capsules (Mylan) |
| 46637 | Capimune 25mg capsules (Mylan) |
| 47042 | Ciclosporin 100mg Capsule (Hillcross Pharmaceuticals Ltd) |
| 47047 | Capsorin 50mg capsules (Morningside Healthcare Ltd) |
| 47102 | Capimune 50mg capsules (Mylan) |
| 47192 | Capsorin 100mg capsules (Morningside Healthcare Ltd) |
| 47377 | Ciclosporin 25mg Capsule (Hillcross Pharmaceuticals Ltd) |
| 47471 | Capsorin 25mg capsules (Morningside Healthcare Ltd) |
| 48556 | Ciclosporin 100mg capsules (Phoenix Healthcare Distribution Ltd) |
| 48763 | Ciclosporin 25mg capsules (Phoenix Healthcare Distribution Ltd) |
| 48798 | Ciclosporin 50mg capsules (Phoenix Healthcare Distribution Ltd) |
| 49958 | Neoral 25mg capsules (DE Pharmaceuticals) |
| 52615 | Neoral 100mg capsules (Sigma Pharmaceuticals Plc) |
| 52743 | Ciclosporin 25mg capsules (Sigma Pharmaceuticals Plc) |
| 53175 | Neoral 25mg capsules (Mawdsley-Brooks & Company Ltd) |
| 53176 | Neoral 50mg capsules (DE Pharmaceuticals) |
| 54134 | Ciclosporin 100mg capsules (Sigma Pharmaceuticals Plc) |
| 54867 | Ciclosporin 50mg capsules (Sigma Pharmaceuticals Plc) |
| 54974 | Ciclosporin 50mg capsules (Cubic Pharmaceuticals Ltd) |
| 54975 | Ciclosporin 100mg capsules (Cubic Pharmaceuticals Ltd) |
| 55116 | Ciclosporin 25mg capsules (Cubic Pharmaceuticals Ltd) |
| 59249 | Ciclosporin 50mg capsules (Colorama Pharmaceuticals Ltd) |
| 59250 | Ciclosporin 100mg capsules (Colorama Pharmaceuticals Ltd) |
| 62051 | Ciclosporin 25mg capsules (Niche Pharma Ltd) |
| 63798 | Ciclosporin 100mg capsules (A A H Pharmaceuticals Ltd) |
| 64857 | Vanquoral 100mg capsules (Teva UK Ltd) |
| 64858 | Vanquoral 50mg capsules (Teva UK Ltd) |
| 65639 | Ciclosporin 100mg capsules (J M McGill Ltd) |
| 66473 | Vanquoral 25mg capsules (Teva UK Ltd) |
| 66785 | Vanquoral 10mg capsules (Teva UK Ltd) |
| 3992 | Deflazacort 6mg tablets |
| 9375 | Deflazacort 1mg tablets |
| 17410 | Deflazacort 30mg tablets |
| 20577 | Calcort 6mg Tablet (Shire Pharmaceuticals Ltd) |
| 22555 | Calcort 1mg tablets (Shire Pharmaceuticals Ltd) |
| 29112 | Calcort 30mg tablets (Shire Pharmaceuticals Ltd) |
| 41335 | Calcort 6mg tablets (Sanofi) |
| 6484 | Sirolimus 2mg tablets |
| 6600 | Sirolimus 1mg tablets |
| 20097 | Sirolimus 1mg/ml oral solution sugar free |
| 23289 | Rapamune 1mg tablets (Pfizer Ltd) |
| 28999 | Rapamune 2mg tablets (Pfizer Ltd) |
| 33728 | Rapamune 1mg/ml oral solution (Pfizer Ltd) |
| 44783 | Sirolimus 500microgram tablets |
| 63210 | Rapamune 0.5mg tablets (Pfizer Ltd) |
| 2839 | Tacrolimus 1mg capsules |
| 3683 | Prograf 1mg capsules (Astellas Pharma Ltd) |
| 5089 | Tacrolimus 5mg capsules |
| 5870 | Prograf 500microgram capsules (Astellas Pharma Ltd) |
| 6495 | Tacrolimus 500microgram capsules |
| 13271 | Prograf 5mg capsules (Astellas Pharma Ltd) |
| 37155 | Tacrolimus 1mg/ml suspension |
| 37506 | Advagraf 1mg modified-release capsules (Astellas Pharma Ltd) |
| 37985 | Tacrolimus 1mg modified-release capsules |
| 38113 | Tacrolimus 500microgram modified-release capsules |
| 38919 | Advagraf 5mg modified-release capsules (Astellas Pharma Ltd) |
| 38989 | Tacrolimus 5mg modified-release capsules |
| 39633 | Advagraf 0.5mg modified-release capsules (Astellas Pharma Ltd) |
| 40765 | Advagraf 3mg modified-release capsules (Astellas Pharma Ltd) |
| 40964 | Tacrolimus 3mg modified-release capsules |
| 43081 | Tacrolimus 1mg granules sachets sugar free |
| 43082 | Tacrolimus 200microgram granules sachets sugar free |
| 44640 | Adoport 0.5mg capsules (Sandoz Ltd) |
| 44641 | Adoport 5mg capsules (Sandoz Ltd) |
| 44804 | Adoport 1mg capsules (Sandoz Ltd) |
| 44926 | Tacrolimus 2.5mg/5ml oral suspension |
| 46324 | Modigraf 0.2mg granules sachets (Astellas Pharma Ltd) |
| 46325 | Modigraf 1mg granules sachets (Astellas Pharma Ltd) |
| 47239 | Vivadex 1mg capsules (Dexcel-Pharma Ltd) |
| 47240 | Vivadex 0.5mg capsules (Dexcel-Pharma Ltd) |
| 47276 | Tacni 1mg capsules (Teva UK Ltd) |
| 47416 | Tacni 0.5mg capsules (Teva UK Ltd) |
| 47432 | Tacrolimus 5mg/5ml oral suspension |
| 47506 | Tacni 5mg capsules (Teva UK Ltd) |
| 47512 | Capexion 1mg capsules (Mylan) |
| 47852 | Capexion 0.5mg capsules (Mylan) |
| 47984 | Capexion 5mg capsules (Mylan) |
| 48339 | Tacrolimus 1mg capsules (A A H Pharmaceuticals Ltd) |
| 51184 | Prograf 1mg capsules (Necessity Supplies Ltd) |
| 51185 | Prograf 500microgram capsules (Necessity Supplies Ltd) |
| 51790 | Tacrolimus 500microgram capsules (A A H Pharmaceuticals Ltd) |
| 52993 | Prograf 1mg capsules (DE Pharmaceuticals) |
| 54048 | Tacrolimus 500micrograms/5ml oral suspension |
| 54198 | Prograf 1mg capsules (Lexon (UK) Ltd) |
| 55010 | Vivadex 5mg capsules (Dexcel-Pharma Ltd) |
| 55066 | Tacrolimus 2.5mg/5ml oral solution |
| 63720 | Tacrolimus 750microgram capsules |
| 63866 | Adoport 2mg capsules (Sandoz Ltd) |
| 63924 | Tacrolimus 2mg capsules |
| 64152 | Tacrolimus 1mg modified-release tablets |
| 65704 | Adoport 0.75mg capsules (Sandoz Ltd) |
| 70334 | Envarsus 750microgram modified-release tablets (Chiesi Ltd) |
| 796 | Copaxone 20mg/ml Injection (Teva UK Ltd) |
| 831 | Methyl aminolevulinate 16% cream |
| 10652 | Akrotherm Cream (Manufacturer unknown) |
| 25904 | Arsenic 10mg/10ml solution for infusion ampoules |
| 31179 | Glatiramer acetate 20mg/ml Injection |
| 39791 | Metvix 16% cream (Galderma (UK) Ltd) |
| 43120 | Trisenox 10mg/10ml concentrate for solution for infusion ampoules (Teva UK Ltd) |
| 45035 | Ceplene 0.5mg/0.5ml solution for injection vials (Meda Pharmaceuticals Ltd) |
| 51435 | Copaxone 20mg/1ml solution for injection pre-filled syringes (Teva UK Ltd) |
| 53998 | Fingolimod 500microgram capsules |
| 55586 | Glatiramer acetate 20mg/1ml solution for injection pre-filled syringes |
| 57445 | Histamine dihydrochloride 500micrograms/0.5ml solution for injection vials |
| 57735 | Gilenya 0.5mg capsules (Novartis Pharmaceuticals UK Ltd) |
| 59362 | Trabectedin 1mg powder for solution for infusion vials |
| 60112 | Erivedge 150mg capsules (Roche Products Ltd) |
| 62009 | Trabectedin 250microgram powder for solution for infusion vials |
| 62759 | Eribulin 880micrograms/2ml solution for injection vials |
| 62880 | Teriflunomide 14mg tablets |
| 63202 | Tafinlar 75mg capsules (Novartis Pharmaceuticals UK Ltd) |
| 64388 | Nintedanib 100mg capsules |
| 64427 | Idelalisib 150mg tablets |
| 65085 | Zydelig 100mg tablets (Gilead Sciences International Ltd) |
| 65140 | Vismodegib 150mg capsules |
| 65784 | Dabrafenib 50mg capsules |
| 66728 | Idelalisib 100mg tablets |
| 68805 | Nintedanib 150mg capsules |
| 69900 | Tafinlar 50mg capsules (Novartis Pharmaceuticals UK Ltd) |
| 70068 | Aubagio 14mg tablets (Genzyme Therapeutics Ltd) |

### Solid organ transplant

#### SOM Medcodes

| **Medcode** | **Description** |
| --- | --- |
| 242 | Allotransplantation of heart NEC |
| 250 | Transplantation of heart and lung |
| 2997 | Transplantation of kidney |
| 4405 | Transplantation of liver |
| 4438 | Other transplantation of heart |
| 5504 | Transplantation of kidney NOS |
| 5911 | [V]Kidney transplanted |
| 6631 | H/O: heart valve recipient |
| 6692 | Liver transplant failure and rejection |
| 9026 | [V]Liver transplanted |
| 9384 | [V]Heart transplanted |
| 10394 | [V]Lung transplanted |
| 10461 | Transplantation of lung |
| 11113 | Transplanted organ rejection |
| 11553 | Kidney transplant failure and rejection |
| 11745 | Transplantation of kidney from live donor |
| 18774 | Renal transplant with complication, without blame |
| 18780 | [V]Heart valve transplanted |
| 22120 | Graft complications |
| 22653 | [V]Transplanted organ or tissue |
| 22728 | Acute graft-versus-host disease |
| 23364 | Repair of artery using prosthetic graft NEC |
| 24361 | Transplantation of kidney from cadaver |
| 25695 | Chronic graft-versus-host disease |
| 25896 | Transplanted organ complication NOS |
| 27319 | Transplantation of liver NOS |
| 27679 | Heart-lung transplant failure and rejection |
| 29831 | Transplanted organ failure |
| 30052 | Transplant complications |
| 31556 | Allograft replacement of coronary artery |
| 31997 | Liver transplant with complication, without blame |
| 32025 | Orthotopic transplantation of liver |
| 32651 | Allograft bypass of coronary artery |
| 35368 | Transplantation of pancreas |
| 36060 | H/O: cornea recipient |
| 36960 | [V]Heart and lungs transplant status |
| 37198 | H/O: liver recipient |
| 38011 | Transplantation of lung NOS |
| 39315 | H/O: skin recipient |
| 39763 | Xenograft replacement of aortic valve |
| 41495 | Other transplantation of heart NOS |
| 43778 | Allograft replacement of aortic valve |
| 43923 | Allograft replacement of pulmonary valve |
| 44077 | [V]Pancreas transplanted |
| 44893 | Transplanted organ complication |
| 45370 | Allograft replacement of four or more coronary arteries |
| 45886 | Allograft replacement of three coronary arteries |
| 46830 | Laryngotracheal reconstruction using cartilage graft |
| 47484 | Heart transplant failure and rejection |
| 47495 | H/O:tissue/organ recipient NOS |
| 47861 | Exploration of liver transplant |
| 48121 | Transplant nephrectomy |
| 48767 | Allograft replacement of coronary artery NOS |
| 49028 | H/O: kidney recipient |
| 49156 | Allograft to organ NOC |
| 51658 | Xenograft replacement of mitral valve |
| 52287 | Closure defect of interatrial septum using tissue graft NEC |
| 53626 | Allotransplantation of heart and lung |
| 54990 | Kidney transplant with complication, without blame |
| 55151 | Autotransplant of kidney |
| 56993 | Transplantation of whole pancreas |
| 57241 | Allograft replacement of two coronary arteries |
| 57403 | H/O: tissue/organ recipient |
| 58518 | Graft to organ NOC |
| 58884 | H/O: bone tissue recipient |
| 59394 | H/O: heart recipient |
| 59423 | Other specified allograft replacement of coronary artery |
| 59610 | Transplant immunosuppression |
| 60955 | Transplantation of islets of Langerhans |
| 61073 | Transplantation of heart and lung NOS |
| 64438 | Heart transplant with complication, without blame |
| 65772 | H/O: lung recipient |
| 66456 | [V]Other specified transplanted organ or tissue |
| 66705 | Allotransplantation of kidney from live donor |
| 67483 | Close defect interventricular septum using tissue graft NEC |
| 67499 | Transplantation of pancreas NOS |
| 69189 | Xenograft replacement of valve of heart NEC |
| 69194 | Replacement of previous liver transplant |
| 69314 | Xenograft to organ NOC |
| 69734 | Other specified other transplantation of heart |
| 70105 | Revision of implantation of prosthetic heart |
| 70111 | Allograft replacement of one coronary artery |
| 70353 | Aortic root replacement using homograft |
| 70712 | Det.ren.func.after ren.transpl |
| 70874 | Other specified transplantation of kidney |
| 71422 | Heterotopic transplantation of liver |
| 72004 | Excision of rejected transplanted kidney |
| 72092 | [V]Unspecified transplanted organ or tissue |
| 72761 | Allograft replacement of valve of heart NEC |
| 72939 | Xenotransplantation of heart |
| 73743 | Other specified transplantation of lung |
| 73817 | Closure defect atrioventricular septum using tissue graft |
| 89445 | Auxillary liver transplant |
| 89924 | Allotransplantation of kidney from cadaver, heart-beating |
| 89936 | Xenograft replacement of pulmonary valve |
| 90275 | Reconstruction of trachea using graft |
| 90952 | Pre-transplantation of kidney work-up, recipient |
| 93158 | Allograft replacement of mitral valve |
| 93366 | Interventions associated with transplantation of kidney |
| 93713 | Single lung transplant |
| 93751 | [V]Intestine transplanted |
| 93844 | Revision of transplantation of heart NEC |
| 94964 | Post-transplantation of kidney examination, live donor |
| 95532 | Aortoventriculoplasty with pulmonary valve autograft |
| 96129 | Excision of transplanted pancreas |
| 96133 | Allotransplantation kidney from cadaver, heart non-beating |
| 96423 | [V]Transplanted organ |
| 96578 | Double lung transplant |
| 97012 | Graft to organ NOC NOS |
| 97157 | Orthotopic transplantation of liver NEC |
| 98173 | Transluminal aortic valve implantation |
| 98364 | Allotransplantation of kidney from cadaver |
| 98580 | Allograft replacement of tricuspid valve |
| 98783 | Xenograft replacement of tricuspid valve |
| 99250 | Other specified transplantation of liver |
| 99847 | Post-transplant lymphoproliferative disorder |
| 100073 | Piggy back liver transplant |
| 100482 | Transapical aortic valve implantation |
| 100621 | Transplantation of ileum |
| 101231 | Transplantation of tail of pancreas |
| 102998 | Transplantation of thymus gland |
| 103429 | Post-transplantation of kidney examination, recipient |
| 104049 | Interventions associated with transplantation of kidney NOS |
| 104050 | OS interventions associated with transplantation of kidney |
| 104201 | Acute rejection of renal transplant |
| 104630 | Acute rejection of renal transplant - grade III |
| 104708 | Repair defect of atrioventricular septum using tissue graft |
| 104905 | Acute-on-chronic rejection of renal transplant |
| 104960 | Acute rejection of renal transplant - grade I |
| 105328 | Cadaveric renal transplant |
| 105506 | Orthotopic transplantation of whole liver |
| 105724 | Unexplained episode of renal transplant dysfunction |
| 105787 | Xenograft renal transplant |
| 105811 | Renal transplant rejection |
| 106015 | Transplantation of spleen |
| 106301 | Stenosis of vein of transplanted kidney |
| 106620 | Chronic rejection of renal transplant |
| 106866 | Vascular complication of renal transplant |
| 107000 | Acute rejection of renal transplant - grade II |
| 107416 | Piggyback transplantation of heart |

#### SOM ICD-10 codes

| **ICD-10** | **Description** |
| --- | --- |
| Z94.0 | Kidney transplant status |
| Z94.1 | Heart transplant status |
| Z94.2 | Lung transplant status |
| Z94.3 | Heart and lungs transplant status |
| Z94.4 | Liver transplant status |
| Z94.5 | Skin transplant status |
| Z94.6 | Bone transplant status |
| Z94.7 | Corneal transplant status |
| Z94.9 | Transplanted organ and tissue status, unspecified |

### Oral corticosteroids Prodcodes

| **Prodcode** | **Description** |
| --- | --- |
| 44 | Prednisolone 5mg gastro-resistant tablets |
| 95 | Prednisolone 5mg tablets |
| 186 | Dexamethasone 500micrograms/5ml oral solution |
| 229 | Cortisone 25mg tablets |
| 557 | Prednisolone 2.5mg gastro-resistant tablets |
| 578 | Prednisolone 1mg tablets |
| 955 | Prednisolone 5mg soluble tablets |
| 1063 | Prednesol 5mg Tablet (Sovereign Medical Ltd) |
| 1280 | Dexamethasone 2mg tablets |
| 1380 | Entocort CR 3mg capsules (Tillotts Pharma Ltd) |
| 1709 | HYDROCORTISONE PELLETS 2.5 MG LOZ |
| 1971 | Betnesol 500microgram soluble tablets (Focus Pharmaceuticals Ltd) |
| 2044 | PREDNISONE 2.5 MG TAB |
| 2130 | Methylprednisolone 4mg tablets |
| 2368 | Prednisolone 2.5mg tablet |
| 2390 | PREDNISOLONE E/C 1 MG TAB |
| 2434 | Florinef 100microgram tablets (Aspen Pharma Trading Ltd) |
| 2439 | Fludrocortisone 100microgram tablets |
| 2470 | FLUDROCORTISONE ACETATE 100 MCG TAB |
| 2704 | Prednisolone 25mg tablets |
| 2799 | PREDNISOLONE 10 MG TAB |
| 2949 | Prednisone 5mg tablets |
| 3059 | PREDNISOLONE 50 MG TAB |
| 3418 | Hydrocortisone 10mg tablets |
| 3557 | Prednisone 1mg tablets |
| 3898 | Budesonide 3mg gastro-resistant modified-release capsules |
| 3969 | DEXAMETHASONE 8 MG TAB |
| 4535 | Hydrocortisone 20mg tablets |
| 4779 | Dexamethasone 500microgram tablets |
| 4943 | Dexamethasone 2mg/5ml oral solution sugar free |
| 5157 | Dexamethasone 2mg/5ml oral solution |
| 5490 | Deltacortril 5mg gastro-resistant tablets (Alliance Pharmaceuticals Ltd) |
| 5913 | Deltacortril 2.5mg gastro-resistant tablets (Alliance Pharmaceuticals Ltd) |
| 6095 | Budesonide 3mg gastro-resistant capsules |
| 6098 | Hydrocortone 10mg tablets (Auden McKenzie (Pharma Division) Ltd) |
| 7286 | Betamethasone 500microgram soluble tablets sugar free |
| 7548 | Cortisone 5mg capsules |
| 7584 | PREDNISOLONE 4 MG TAB |
| 7710 | PREDNISOLONE 15 MG TAB |
| 7934 | PREDNISONE 30 MG TAB |
| 8165 | FLUDROCORTISONE 25 MCG TAB |
| 8261 | Medrone 16mg tablets (Pfizer Ltd) |
| 9727 | Prednisolone 50mg tablets |
| 9994 | Decadron 500microgram tablets (Merck Sharp & Dohme Ltd) |
| 10431 | DELTACORTRIL 1 MG TAB |
| 10552 | Methylprednisolone 16mg tablets |
| 10574 | Cortisone acetate 5mg tablets |
| 10683 | Medrone 2mg tablets (Pfizer Ltd) |
| 10684 | Methylprednisolone 2mg tablets |
| 10754 | Hydrocortistab 20mg Tablet (Waymade Healthcare Plc) |
| 10864 | Betamethasone 500microgram tablets |
| 11149 | Betnelan 500microgram tablets (Focus Pharmaceuticals Ltd) |
| 12398 | Cortelan 25mg Tablet (Glaxo Laboratories Ltd) |
| 12400 | Cortisyl 25mg Tablet (Aventis Pharma) |
| 13043 | Hydrocortone 20mg tablets (Auden McKenzie (Pharma Division) Ltd) |
| 13512 | ORADEXON .5 MG TAB |
| 13522 | PREDNISOLONE 2 MG TAB |
| 13615 | PREDNISONE 10 MG TAB |
| 14076 | Hydrocortisone 5mg/5ml Oral solution |
| 14172 | Methylprednisolone 100mg tablets |
| 15471 | HYDROCORTISONE 25 MG TAB |
| 15555 | Medrone 4mg tablets (Pfizer Ltd) |
| 15595 | FLUDROCORTISONE .05 MG SUS |
| 15617 | Ledercort 4mg Tablet (Wyeth Pharmaceuticals) |
| 16525 | Budenofalk 3mg gastro-resistant capsules (Dr. Falk Pharma UK Ltd) |
| 16724 | PREDNISONE 50 MG TAB |
| 17263 | HYDROCORTISONE SODIUM PHOSPHATE 5 MG SOL |
| 18042 | Medrone 100mg tablets (Pfizer Ltd) |
| 18637 | Cortistab 25mg Tablet (Waymade Healthcare Plc) |
| 18955 | HYDROCORTISONE 4.5 MG LOZ |
| 19141 | Prednisolone 5mg soluble tablets (AMCo) |
| 19908 | Triamcinolone 2mg Tablet |
| 20095 | Precortisyl forte 25mg Tablet (Aventis Pharma) |
| 21056 | FLUDROCORTISONE 75 MCG TAB |
| 21077 | FLUDROCORTISONE 20 MCG TAB |
| 21218 | Dexsol 2mg/5ml oral solution (Rosemont Pharmaceuticals Ltd) |
| 21417 | Prednisolone 5mg tablets (A A H Pharmaceuticals Ltd) |
| 21833 | Decortisyl 5mg Tablet (Roussel Laboratories Ltd) |
| 21903 | Oradexon-organon 2mg Tablet (Organon Laboratories Ltd) |
| 22894 | HYDROCORTISONE 4 MG PAS |
| 23111 | Triamcinolone 4mg Tablet |
| 23210 | Cortistab 5mg Tablet (Waymade Healthcare Plc) |
| 23512 | Precortisyl 5mg Tablet (Hoechst Marion Roussel) |
| 24014 | Ledercort 2mg Tablet (Wyeth Pharmaceuticals) |
| 25272 | Precortisyl 1mg Tablet (Hoechst Marion Roussel) |
| 25621 | FLORINEF .2 MG TAB |
| 26806 | DECADRON 2 MG TAB |
| 27962 | Deltastab 1mg Tablet (Waymade Healthcare Plc) |
| 28375 | Prednisolone 2.5mg gastro-resistant tablets (A A H Pharmaceuticals Ltd) |
| 28376 | Prednisolone 2.5mg Gastro-resistant tablet (Biorex Laboratories Ltd) |
| 28615 | METHYLPREDNISOLONE L/A 4 MG CAP |
| 28859 | Deltastab 5mg Tablet (Waymade Healthcare Plc) |
| 29333 | Prednisolone 5mg tablets (Actavis UK Ltd) |
| 30390 | DELTASTAB 2 MG TAB |
| 30971 | DECORTISYL 25 MG TAB |
| 31327 | Prednisolone steaglate 6.65mg tablet |
| 31532 | Prednisolone 5mg gastro-resistant tablets (A A H Pharmaceuticals Ltd) |
| 32664 | BECLAZONE 200 |
| 32803 | Prednisolone 5mg gastro-resistant tablets (Actavis UK Ltd) |
| 32835 | Prednisolone 5mg tablets (Wockhardt UK Ltd) |
| 33691 | Prednisolone 5mg Gastro-resistant tablet (Biorex Laboratories Ltd) |
| 33988 | Prednisolone 5mg Tablet (Co-Pharma Ltd) |
| 33990 | Prednisolone 5mg Tablet (IVAX Pharmaceuticals UK Ltd) |
| 34393 | Prednisolone 5mg gastro-resistant tablets (Teva UK Ltd) |
| 34404 | Prednisolone 1mg tablets (Actavis UK Ltd) |
| 34452 | Prednisolone 1mg tablets (A A H Pharmaceuticals Ltd) |
| 34461 | Prednisolone 2.5mg gastro-resistant tablets (Actavis UK Ltd) |
| 34631 | Prednisolone 1mg Tablet (Co-Pharma Ltd) |
| 34660 | Prednisolone 1mg tablets (Kent Pharmaceuticals Ltd) |
| 34748 | Prednisolone 1mg tablets (Teva UK Ltd) |
| 34781 | Prednisolone 5mg tablets (Kent Pharmaceuticals Ltd) |
| 34801 | Dexamethasone 0.5mg/5ml Oral solution (Rosemont Pharmaceuticals Ltd) |
| 34880 | Dexamethasone 2mg tablets (Aspen Pharma Trading Ltd) |
| 34914 | Prednisolone 1mg Tablet (Celltech Pharma Europe Ltd) |
| 34915 | Dexamethasone 500microgram tablets (Organon Laboratories Ltd) |
| 34978 | Prednisolone 1mg tablets (Wockhardt UK Ltd) |
| 36055 | Dexamethasone 2mg Tablet (Hillcross Pharmaceuticals Ltd) |
| 37203 | Beclometasone 5mg gastro-resistant modified-release tablets |
| 38022 | Hydrocortisone 10mg/5ml oral suspension |
| 38407 | Prednisolone 20mg tablet |
| 39067 | Clipper 5mg gastro-resistant modified-release tablets (Chiesi Ltd) |
| 41515 | Prednisolone 5mg tablets (Teva UK Ltd) |
| 41745 | Prednisolone 25mg tablets (Zentiva) |
| 43544 | Prednisone 5mg Tablet (Knoll Ltd) |
| 44380 | Prednisone 1mg modified-release tablets |
| 44723 | Prednisone 5mg modified-release tablets |
| 44802 | Lodotra 5mg modified-release tablets (Napp Pharmaceuticals Ltd) |
| 44803 | Lodotra 2mg modified-release tablets (Napp Pharmaceuticals Ltd) |
| 45234 | Dexamethasone 100microgram capsules |
| 45302 | Prednisolone 5mg Tablet (Biorex Laboratories Ltd) |
| 46711 | Prednisone 2mg modified-release tablets |
| 47142 | Prednisolone 5mg Soluble tablet (Amdipharm Plc) |
| 47225 | Budesonide 9mg gastro-resistant granules sachets |
| 48088 | Budenofalk 9mg gastro-resistant granules sachets (Dr. Falk Pharma UK Ltd) |
| 50225 | Betnesol 500microgram soluble tablets (Waymade Healthcare Plc) |
| 51722 | Hydrocortisone 5mg/5ml oral suspension |
| 51753 | Prednisolone 1mg tablets (Strides Shasun (UK) Ltd) |
| 51824 | Hydrocortisone 5mg/5ml oral suspension sugar free |
| 51849 | Hydrocortisone 1mg/5ml oral suspension |
| 51871 | Hydrocortisone 2mg capsules |
| 51872 | Hydrocortisone 2.5mg capsules |
| 51997 | Budesonide 9mg gastro-resistant granules sachets |
| 52053 | Hydrocortisone 3mg/5ml oral suspension |
| 52396 | Dexamethasone 1mg/5ml oral solution |
| 52472 | Fludrocortisone 50micrograms/5ml oral suspension |
| 53143 | Cortisone 25mg tablets (A A H Pharmaceuticals Ltd) |
| 53313 | Prednisolone 20mg/5ml oral suspension |
| 53336 | Prednisolone 25mg tablets (A A H Pharmaceuticals Ltd) |
| 53705 | Cortisone acetate 5mg Capsule (Martindale Pharmaceuticals Ltd) |
| 54118 | Prednisolone 25mg/5ml oral suspension |
| 54432 | Lodotra 1mg modified-release tablets (Napp Pharmaceuticals Ltd) |
| 54434 | Prednisolone 2.5mg/5ml oral suspension |
| 54793 | Dexamethasone 2mg/5ml oral suspension |
| 55024 | Prednisolone 5mg/5ml oral solution |
| 55401 | Dexamethasone 500microgram tablets (A A H Pharmaceuticals Ltd) |
| 55480 | Prednisolone 2.5mg gastro-resistant tablets (Alliance Pharmaceuticals Ltd) |
| 56144 | Budenofalk 9mg gastro-resistant granules sachets (Dr. Falk Pharma UK Ltd) |
| 56347 | Dexamethasone 5mg/5ml oral solution |
| 56443 | Dexamethasone 10mg/5ml oral solution |
| 56891 | Prednisolone 1mg tablets (Waymade Healthcare Plc) |
| 57931 | Hydrocortisone 20mg tablets (Teva UK Ltd) |
| 58000 | Prednisolone 5mg tablets (Almus Pharmaceuticals Ltd) |
| 58061 | Prednisone 50mg tablets |
| 58234 | Prednisolone 10mg/5ml oral solution |
| 58369 | Prednisolone 5mg tablets (Boston Healthcare Ltd) |
| 58384 | Prednisolone 1mg tablets (Almus Pharmaceuticals Ltd) |
| 58474 | Dexamethasone 2mg/5ml oral solution sugar free (A A H Pharmaceuticals Ltd) |
| 58987 | Prednisolone 5mg gastro-resistant tablets (Phoenix Healthcare Distribution Ltd) |
| 59229 | Dilacort 5mg gastro-resistant tablets (Auden McKenzie (Pharma Division) Ltd) |
| 59283 | Dilacort 2.5mg gastro-resistant tablets (Auden McKenzie (Pharma Division) Ltd) |
| 59338 | Prednisolone 1mg/5ml oral solution |
| 59912 | Prednisolone 5mg gastro-resistant tablets (Waymade Healthcare Plc) |
| 60064 | Dexamethasone 10mg/5ml oral solution sugar free |
| 60120 | Dexamethasone 2mg tablets (Alliance Healthcare (Distribution) Ltd) |
| 60421 | Prednisolone 5mg tablets (Strides Shasun (UK) Ltd) |
| 60516 | Fludrocortisone 50microgram capsules |
| 60946 | Entocort CR 3mg capsules (Waymade Healthcare Plc) |
| 61132 | Prednisolone 1mg tablets (Boston Healthcare Ltd) |
| 61162 | Prednisolone 5mg tablets (Waymade Healthcare Plc) |
| 61689 | Prednisolone 5mg soluble tablets (A A H Pharmaceuticals Ltd) |
| 62656 | Prednisone 5mg Tablet (Hillcross Pharmaceuticals Ltd) |
| 62909 | Dexamethasone 2mg tablets (A A H Pharmaceuticals Ltd) |
| 63066 | Prednisolone 2.5mg tablets |
| 63082 | Prednisolone 20mg tablets |
| 63172 | Prednisolone 10mg tablets |
| 63214 | Prednisolone 5mg soluble tablets (Alliance Healthcare (Distribution) Ltd) |
| 63549 | Prednisolone 1mg/ml oral solution (Logixx Pharma Solutions Ltd) |
| 63791 | Prednisolone 5mg/5ml oral solution unit dose |
| 63893 | Budesonide 9mg modified-release tablets |
| 64007 | Pevanti 10mg tablets (AMCo) |
| 64008 | Pevanti 2.5mg tablets (AMCo) |
| 64009 | Pevanti 20mg tablets (AMCo) |
| 64050 | Martapan 2mg/5ml oral solution (Martindale Pharmaceuticals Ltd) |
| 64059 | Hydrocortisone 2.5mg/5ml oral suspension |
| 64128 | Pevanti 5mg tablets (AMCo) |
| 64221 | Prednisolone 5mg/5ml oral suspension |
| 64235 | Betamethasone 500microgram soluble tablets sugar free (Alliance Healthcare (Distribution) Ltd) |
| 64416 | Prednisolone 10mg/ml oral solution sugar free |
| 64475 | Fludrocortisone 25micrograms/5ml oral suspension |
| 64557 | Cortiment 9mg modified-release tablets (Ferring Pharmaceuticals Ltd) |
| 64747 | Dexamethasone 2mg/5ml oral solution |
| 64766 | Dexamethasone 20mg/5ml oral solution sugar free |
| 64787 | Hydrocortisone 10mg tablets (Almus Pharmaceuticals Ltd) |
| 64988 | Fludrocortisone 150micrograms/5ml oral suspension |
| 65020 | Prednisolone 25mg/5ml oral solution |
| 65626 | Prednisolone 10mg/5ml oral suspension |
| 65984 | Hydrocortisone 10mg tablets (Actavis UK Ltd) |
| 66015 | Prednisolone Dompe 5mg/5ml oral solution unit dose (Logixx Pharma Solutions Ltd) |
| 66200 | Dexamethasone 2mg soluble tablets sugar free |
| 66287 | Dexamethasone 8mg soluble tablets sugar free |
| 66327 | Hydrocortisone 20mg tablets (Actavis UK Ltd) |
| 66524 | Dexamethasone 4mg soluble tablets sugar free |
| 66550 | Prednisolone 5mg gastro-resistant tablets (Alliance Healthcare (Distribution) Ltd) |
| 66645 | Prednisolone 5mg/5ml oral solution unit dose (Logixx Pharma Solutions Ltd) |
| 66666 | Hydrocortisone 10mg tablets (Teva UK Ltd) |
| 66724 | Dexamethasone 10mg capsules |
| 66914 | Prednisolone 1mg gastro-resistant tablets |
| 67076 | Prednisolone 20mg/5ml oral solution |
| 67107 | Prednisolone 5mg gastro-resistant tablets (Alliance Pharmaceuticals Ltd) |
| 67507 | Prednisolone 30mg tablets |
| 67559 | Prednisolone 5mg/5ml oral solution unit dose (A A H Pharmaceuticals Ltd) |
| 68103 | Dexamethasone 2mg soluble tablets sugar free (A A H Pharmaceuticals Ltd) |
| 68166 | Fludrocortisone 20micrograms/5ml oral suspension |
| 68182 | Dexamethasone 2mg tablets (Teva UK Ltd) |
| 68306 | Betamethasone 500microgram soluble tablets sugar free (Focus Pharmaceuticals Ltd) |
| 68489 | Dexamethasone 4mg tablets |
| 68497 | Prednisolone 2.5mg gastro-resistant tablets (Waymade Healthcare Plc) |
| 68593 | Dexamethasone 5mg/5ml oral suspension |
| 68673 | Fludrocortisone 100microgram tablets (A A H Pharmaceuticals Ltd) |
| 68693 | Fludrocortisone 10micrograms/5ml oral suspension |
| 68860 | Dexamethasone 8mg soluble tablets sugar free (A A H Pharmaceuticals Ltd) |
| 69568 | Dilacort 5mg gastro-resistant tablets (Crescent Pharma Ltd) |
| 69572 | Dexamethasone 4mg/5ml oral suspension |
| 69686 | Pevanti 25mg tablets (AMCo) |
| 69811 | Prednisolone 30mg tablets (Actavis UK Ltd) |
| 70603 | Prednisolone 5mg soluble tablets (Focus Pharmaceuticals Ltd) |
| 70611 | Dexamethasone 3mg/5ml oral solution |
| 70893 | Dexamethasone 40mg tablets |
| 71404 | Dexamethasone 2mg/5ml oral solution sugar free (Waymade Healthcare Plc) |

# Appendix B: Supplementary Tables

**Table 1**: Person years (%) by risk factors and vaccination status

|  | | **Person years (%)** | | |
| --- | --- | --- | --- | --- |
|  | | **All** | **Unvaccinated** | **Vaccinated** |
| **All** | | 255,719(100) | 178,229(70) | 77,490(30) |
| **Age** | | | | |
|  | Routine cohort (70 yrs) | 143,122(56) | 101,001(71) | 42,121(29) |
|  | Catch-up cohort (78-79 yrs) | 112,597 (44) | 77,228(69) | 35,369(31) |
| **Gender** | | | | |
|  | Male | 120,227(47) | 83,494(69) | 36,733(31) |
|  | Female | 135,491(53) | 94,734(70) | 40,757(30) |
| **Ethnicity*** | | | | |
|  | Caucasian | 226,159(88) | 155,707(69) | 70,452(31) |
|  | Non-Caucasian | 9,467(3.7) | 6,993(74) | 2,474(26) |
| **Index of Multiple Deprivation (IMD)** | | | | |
|  | <5^th^ quintile | 227,220(89) | 157,422(69) | 69,798(31) |
|  | 5^th^ quintile | 28,418(11) | 20,744(73) | 7,674(27) |
| **Chronic conditions** | | | | |
|  | Asthma | 36,653(14) | 25,132(69) | 11,520(31) |
|  | Type 2 diabetes | 50,480(20) | 34,460(68) | 16,020(32) |
|  | COPD | 20,092(7.9) | 14,234(71) | 5,858(29) |
| **Smoking*** | | | | |
|  | Non-smoker | 114,190(45) | 77,434(68) | 36,757(32) |
|  | Ex-smoker | 83,665(33) | 57,061(68) | 26,604(32) |
|  | Smoker | 26,352(10) | 20,368(77) | 5,984(23) |
| **Body mass index (BMI)*** | | | | |
|  | Normal weight | 54,371(21) | 37,062(68) | 17,309(32) |
|  | Underweight | 7,449(2.9) | 5,476(74) | 1,973(26) |
|  | Overweight or obese | 140,444(55) | 95,361(68) | 45,083(32) |
| **Contraindications or precautions for zoster vaccination** | | | | |
|  | Immunocompromised | 6,289(2.5) | 4,791(76) | 1,498(24) |
|  | Immunosuppressed | 5,822(2.3) | 4,280(74) | 1,542(26) |
| **Previous exposure to the antigen** | | | | |
|  | History of herpes zoster prior to study inclusion | 28,239(11) | 19,384(69) | 8,855(31) |
| **Co-administration of zoster vaccine** | | | | |
|  | With influenza vaccine | 28,782(11) | - | 28,782(100) |
|  | With pneumococcal vaccine | 923(0.36) | - | 923(100) |
